# BVSim: A benchmarking variation simulator mimicking human variation spectrum

**DOI:** 10.1093/gigascience/giaf095

**Published:** 2025-08-30

**Authors:** Yongyi Luo, Zhen Zhang, Shu Wang, Jiandong Shi, Jingyu Hao, Sheng Lian, Taobo Hu, Toyotaka Ishibashi, Depeng Wang, Weichuan Yu, Xiaodan Fan

**Affiliations:** Department of Statistics, The Chinese University of Hong Kong, Shatin, New Territories, Hong Kong 999077, China; Department of Electronic and Computer Engineering, The Hong Kong University of Science and Technology, Clear Water Bay, Kowloon, Hong Kong 999077, China; Department of Breast Surgery, Peking University People’s Hospital, No. 11, Xizhimen South St, Beijing 100044, China; Department of Electronic and Computer Engineering, The Hong Kong University of Science and Technology, Clear Water Bay, Kowloon, Hong Kong 999077, China; Department of Electronic and Computer Engineering, The Hong Kong University of Science and Technology, Clear Water Bay, Kowloon, Hong Kong 999077, China; Department of Electronic and Computer Engineering, The Hong Kong University of Science and Technology, Clear Water Bay, Kowloon, Hong Kong 999077, China; Department of Breast Surgery, Peking University People’s Hospital, No. 11, Xizhimen South St, Beijing 100044, China; Division of Life Science, Hong Kong University of Science and Technology, Clear Water Bay, Kowloon, Hong Kong 999077, China; GrandOmics Biosciences, No.1, East Nengyuan Road, Beijing 102200, China; Department of Electronic and Computer Engineering, The Hong Kong University of Science and Technology, Clear Water Bay, Kowloon, Hong Kong 999077, China; Department of Statistics, The Chinese University of Hong Kong, Shatin, New Territories, Hong Kong 999077, China

**Keywords:** genomic variations, sequence simulation, benchmarking

## Abstract

**Background:**

Genomic variations, including single-nucleotide polymorphisms, small insertions and deletions, and structural variations, are crucial for understanding evolution and disease. However, comprehensive simulation tools for benchmarking genomic analysis methods are lacking. Existing simulators do not accurately represent the nonuniform distribution and length patterns of structural variations in human genomes, and simulating complex structural variations remains challenging.

**Results:**

We present BVSim, a flexible tool that provides probabilistic simulations of genomic variations, primarily focusing on human patterns while accommodating diverse species. BVSim effectively simulates both simple and complex structural variations and small variants by mimicking real-life variation distributions, which often exhibit higher frequencies near telomeres and within tandem repeat regions. Notably, BVSim allows users to input single or multiple benchmark samples from any reference genome, enabling the tool to summarize and represent the unique distribution patterns of structural variation positions and lengths specific to those species. Its compatibility with standard file formats facilitates seamless integration into various genomic research workflows, making it a very useful resource for benchmarking downstream tools such as variant callers. With numerical experiments, we show that BVSim generated more realistic sequences significantly different from other simulators’ outputs.

**Conclusions:**

BVSim is written in Python and freely available to noncommercial users under the GPL3 license. Source code, application guide, and toy examples are provided on the GitHub page at https://github.com/YongyiLuo98/BVSim. The tool is registered in SciCrunch (RRID:SCR_026926), bio.tools (biotools:BVSim), and WorkflowHub (doi:10.48546/WORKFLOWHUB.WORKFLOW.1361.1).

## Background

Genomic variations, including single-nucleotide polymorphisms (SNPs), small insertions and deletions (indels) under 50 base pairs (bp), and structural variations (SVs), are of vital importance due to their close relation with evolution, disease, and so on [1]. There is a huge amount of research about SNPs and small indels with the development of the next-generation (short-read) sequencing technologies [2–4]. However, due to the short read length limitation, SVs (usually defined as longer than 50 bp) cannot be detected accurately. Thus, third-generation (long-read) sequencing technologies have emerged in the past decade, greatly facilitating the understanding of SVs [5]. Importantly, several studies have reported that SV positions in the human genome may not follow a simple uniform distribution. More specifically, SVs tend to occur at higher rates near telomeres and within tandem repeat (TR) regions [6, 7]. Additionally, the length distributions of these SVs exhibit nonuniform patterns. A large-scale study of Icelandic individuals [7] revealed prominent peaks in the SV length distribution at approximately 300 bp, 2,500 bp, and 6,000 bp. Further analysis of publicly available SV data from comprehensive characterizations using 15 representative samples [6], the benchmark dataset HG002 called by 19 SV callers from the Genome in a Bottle (GIAB) study [8, 9], as well as the Human Genome Structural Variation Consortium (HGSVC) samples [10], demonstrated similar nonuniform patterns for both SV locations and lengths (Supplementary Figs. S1–S6, Supplementary Tables S1–S4).

To carry out further studies on SVs as well as SNPs and small indels, it is important to build a realistic and comprehensive simulator of these variations to benchmark the related methods and tools, such as alignment, variation calling, and consensus inference. Existing genome simulators, while able to simulate SVs as well as SNPs and small indels, have limitations in accurately representing SV features. VarSim [11] was among the first tools with SV simulation function. It is primarily tailored for human cancer genome simulation and samples SV positions from fixed regions, limiting its ability to simulate diverse SV distributions. Simulome [12] introduced random variations with different options, but it was primarily designed for prokaryotic genomes. simuG [13] can simulate some SVs more randomly as compared with VarSim, but its parameter tuning capabilities are limited to adjusting overall characteristics, such as the proportion of insertions and deletions, and it can only generate variations uniformly, rather than allowing direct manipulation of specific variation probabilities or counts. SURVIVOR [14] simulates variants only randomly with limited fine-tuning parameters, although it can also evaluate the SV callers. RSVSim [15] applies a parametric model for the SV length distribution, limiting its flexibility to reflect more complex patterns in real data. Although RSVSim simulates breakpoints near some overlapped points from the empirical datasets for the human genome, it relies on preprocessed repeat annotations from hg19 [16] and cannot incorporate newer empirical SV datasets or adapt to custom genomic annotations. The package enforces fixed mechanistic biases through hardcoded repeat associations [17–20], whereas modern approaches require dynamic learning from diverse input data. While Sim-it [21] can simulate customized SVs and long reads, its utility is limited by both the inability to model small indels and SNPs concurrently and by an opaque telomeric bias mechanism lacking adjustable parameters for comparative studies. VISOR [22] allows users to manually input SV positions, but it is overly complicated to simulate a large number of SVs. Mutation-Simulator (MTS) [23] simulates SVs and small variants with more fine-tuning options but cannot accommodate nonuniform deletions or SV length distributions.

Furthermore, complex SVs (CSVs) have been identified in the human genome, particularly in individuals with autism spectrum disorder and other developmental abnormalities [24]. CSVs exhibit more complex genomic rearrangements beyond simple insertions, deletions, tandem duplications, and inversions. VISOR, used in the SVision study [25], serves as a CSV simulator that employs a multistep curation process to generate simulated data for evaluating the detection of CSVs. In the simulation of CSVs with VISOR, not only the position is fixed but also the steps are complicated, limiting both the types and generality of CSVs, which highlights the need for more comprehensive and automated CSV simulation capabilities.

To address these limitations, we present BVSim, a command-line tool that automatically simulates nonuniform and haplotype-resolved variations with randomness. The patterns of simulated variations are derived from benchmark datasets of the human genome, mimicking the human variation spectrum. This tool highlights the importance of simulating a wide range of SVs, from simple to complex, as well as small variants.

Importantly, BVSim allows users to input browser extensible data (BED) files containing empirical variations derived from benchmark datasets of various species or specific human subpopulations. By summarizing SV patterns from these user-supplied datasets, BVSim generates vectors representing the local SV probabilities, supporting realistic simulations of variations across diverse reference genomes and enhancing its utility for researchers studying a wide range of organisms. Furthermore, BVSim enables users to specify parameters for simulating various scenarios for benchmarking purposes, including configurations that control the rate, length, and distribution patterns of genomic variations.

## Findings

**Figure 1:**
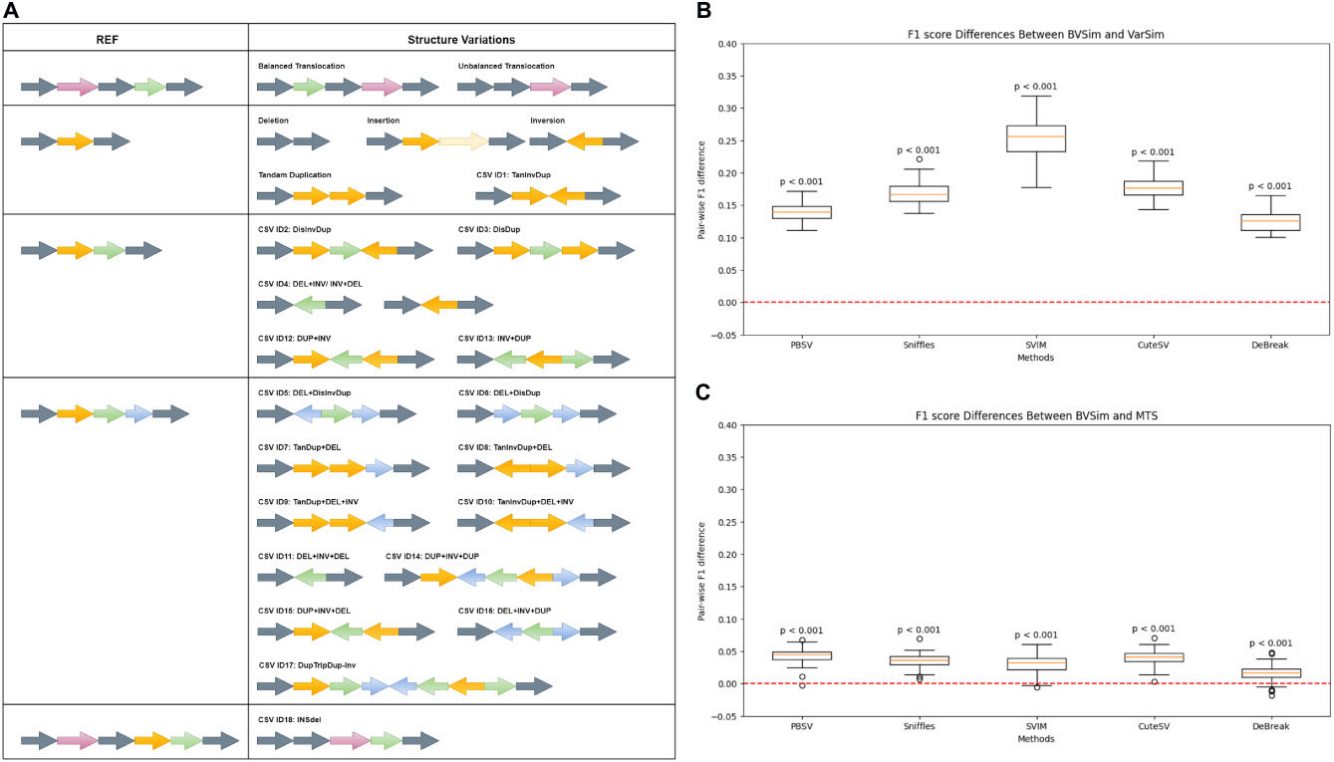
(A) Definitions of the SV types supported by BVSim. (B) A boxplot showing the pairwise differences in F1 scores for variation detection algorithms on 50 datasets, calculated as the F1 scores from BVSim datasets minus those from VarSim. (C) A boxplot illustrating the pairwise differences in F1 scores for variation detection algorithms on 50 datasets, calculated as the F1 scores from BVSim datasets minus those from MTS.

BVSim, a command-line package written in Python, is designed to randomly simulate realistic and comprehensive variations and create human-like genomic sequences by default, with options to learn from input empirical variations of other species. It enables the integration of simple SVs, including deletions, insertions, inversions, tandem duplications, and intrachromosomal translocations, both balanced and unbalanced. In addition, it offers parameters to control rates or numbers of small variations, including small indels and SNPs. Furthermore, it can emulate 18 types of CSVs, illustrated in Fig. 1A [24, 25].

### Methodology

BVSim offers 8 operational modes designed to accommodate diverse simulation requirements. In the default mimic mode, the tool simulates realistic variations by mimicking the variations on the human reference genome hg19 or hg38. At the same time, it maintains the flexibility to accept any reference genome sequence as input. Six other genomic sequence simulation modes (wave mode, wave-region mode, CSV mode, uniform mode, uniform-parallel mode, and exact mode) are provided for diverse application scenarios. The remaining variant call format (VCF) mode provides a preprocessing function when users would like to simulate population-specific empirical SVs.

The tool first learns empirical distributions from the observed data in a nonparametric way, then generates different types of variants sequentially following the order: translocation, inversion, tandem duplications, CSV ID1–ID18 (see Fig. 1A), deletions, insertions, small deletions, small insertions, and SNPs. Later variants will avoid overlapping with previous ones. The sampling method may vary for different variants and different sequence simulation modes.

#### Distribution learning from empirical data

For each variant, if the observation data amount allows, BVSim extracts the variants’ empirical length distribution by counting and spatial distribution along chromosomes by adjustable binning. These empirical distributions will be later used to synthesize such variant by random sampling from them.

Specifically, for a variant type $t$, its length distribution is computed as:


(1) \begin{eqnarray*}
P_t(l) = \frac{n_{t,l}}{\sum _{l^{\prime }} n_{t,l^{\prime }}},
\end{eqnarray*}

where $n_{t,l}$ is the number of observed occurrences of length-$l$ variants for type $t$. The denominator normalizes the distribution with the totals across $N$ individual input samples.

To learn the spatial distribution of a variant type, the genome is partitioned into $M$ bins $\lbrace S_1,...,S_M\rbrace$ of size $L$ (customizable with default value = 500 kbp), with the terminal bin accommodating residual bases:


(2) \begin{eqnarray*}
S_j = \left\lbrace \begin{array}{l{@}{\quad}l}
\left[(j-1)L, jL)\right. & \text{for } j=1,...,M-1 \\
\left[(j-1)L, G\right] & \text{for } j=M
\end{array}\right.,
\end{eqnarray*}

where $G$ is the sequence length and $M$ is the integer part of G/L.

For the case of inputting a single empirical sample (e.g., HG002 [8]), we observe a count $c_j$ of a same variant type in the bin $S_j$. For the case of inputting multiple samples (e.g., the Cell dataset [6] or HGSVC dataset [10]), we observe $c_{j,i}$ for the $j$th bin of the $i$th sample, where $i=1,\cdots , N$ and $j=1,\cdots , M$. Then, we calculate a mean $\mu _j$ and a standard deviation value $\sigma _j^2$ from these $c_{j,i}$s for the $j$th bin.

#### General sampling methods

Different variant types are generated one after the other. For a specific variant type, we first decide the number of variants within each bin, then determine their location within the corresponding bin.

Let $v_{\mathrm{total}}$ be the total number of a specific variant type to be simulated. It is either directly specified by the user (e.g., “-sv_del 100” sets the total of deletions as 100) or determined from the input observed sample(s) if the sum-preserving feature is activated by the “-sum” flag (i.e., $v_{\mathrm{total}}$ is either equal to $\sum \limits _{j=1}^M c_j$ for a single-sample case and $\sum \limits _{j=1}^M \mu _j$ for a multisample case).

The simulated count $k_j$ for the bin $S_j$ ($j=1,\ldots ,M$) satisfies $\sum _{j=1}^M k_j = v_{\mathrm{total}}$. If the user chooses the fixed option to preserve observed distributions, we set $k_j$ as $c_j$ for the single-sample case and the integer part of $\mu _j$ for the multisample case. If the user chooses the random option, $k_j$s will be sampled from the corresponding distribution learned from the empirical data. More specifically, for the multisample case, we sample $c_j$ from a nonnegative discrete normal distribution with mean $\mu _j$ and variance $\sigma _j^2$; for the single-sample case, $c_j$ is observed. Then, for both cases, we sample $(k_1,...,k_M) \sim \text{Multinomial}(v_{\text{total}}, (p_1,...,p_M))$, where $p_j = c_j/\sum _k c_k$.

The start position of each variant within its corresponding bin is sampled uniformly from all available positions within the bin. For the mimic mode and the wave-region mode, we allow users to further specify different variant showing-up probabilities for TR and non-TR regions. A visualization of the wave-region mode is given in Supplementary Fig. S8.

The length of a small indel (1–5 bp) is independently sampled from an empirical distribution calculated from the Database of Genomic Variants (DGV) [26]. More specifically, the probability of a small indel’s length equal to 1, 2, 3, 4, 5 is equal to $\frac{6}{8}, \frac{1}{8}, \frac{1}{16}, \frac{1}{32}, \frac{1}{32}$, respectively.

To simulate a translocation, BVSim samples 2 regions (A and B). A translocation is either balanced (A and B are exchanged) or unbalanced (A is lost and B is inserted into A’s start point) in the sequence. To simulate a duplication, 1 copied region and the inserted position need to be sampled. To simulate an inversion/insertion/deletion, BVSim samples a start point and a length. To simulate a SNP, BVSim samples a location and a replacement base from a learned substitution transition matrix from the dbSNP database [27]. We also ensure that the SVs and small variants will not be simulated from the regions related to other variants. Complex SVs combine these simple variants with spatial proximity. All SVs and CSVs can be generated together with small indels and SNPs.

### Default mode-mimic human

BVSim’s default mode generates variants by mimicking the real human variant spectrum. Specifically, we derive the empirical distributions of variants from individual-level benchmark HG002 (GRCh37/hg19 as reference) [8], the 15 samples published in *Cell* [6], and the 32 *HGSVC* samples (both GRCh38/hg38 as reference) [10]. Calling “-mimic” with “-hg19” or “-hg38” and specifying the chromosome name can activate the procedures to mimic the above built-in empirical distributions. For the hg38 reference, BVSim combines both hg38 datasets (*Cell* and *HGSVC*) by default. Note that their overlapping samples (NA12878, HG02818) are automatically deduplicated during processing. Users can restrict empirical distribution learning to a specific dataset by specifying either the “-cell” or “-hgsvc” flag with the “-hg38” flag.

### Wave mode and wave-region mode

The wave mode triggered by the “-wave” flag utilizes BED file(s) to model variant distributions based on location and length, generating nonuniform profiles that reflect learned distributions. Notably, if multiple benchmark datasets are available, the wave mode allows for the expansion of the population. For instance, it can simulate the genomes of patients with breast cancer.

The wave-region mode triggered by the “-wave_region” flag further enhances this capability by enabling customized variant probabilities for specific genomic regions, such as TR regions, allowing for varied variant densities compared to the overall genomic sequence. Both modes enhance simulation efficiency by parallelizing variant generation through sequence segmentation.

### CSV mode

The CSV mode triggered by the “-csv” flag can generate the 18 CSV types and other SVs defined in Fig. 1A, together with small variants following user-defined quantities or rates. Users can customize CSV length distributions and introduce or avoid other variations.

### Uniform mode and uniform-parallel mode

The uniform mode is triggered by the “-uniform” flag, while the uniform-parallel mode is triggered by adding the “-cores” parameter. Both modes distribute variants uniformly across the input sequence while respecting nonoverlapping constraints and blocked regions. Uniform mode is recommended when no additional information is available about the species or when the simulation scale is small. For long references or when simulating numerous variations, the uniform-parallel mode is recommended. This mode distributes the uniform variation simulation across multiple processes, significantly accelerating the simulation process.

### Exact mode

Calling “-exact” will activate the exact mode, which enables precise simulation of variants at user-specified genomic positions through a structured table format. The input requires 3 key pieces of information for each variant: genomic location, variant type, and length, with additional fields required for specific variant classes. Balanced translocations must include coordinates for both exchanged regions, while tandem duplications require the source and destination positions. Complex variants can be constructed by specifying multiple simple variants that collectively form the composite event.

### VCF mode

The VCF mode serves as a preprocessing step for population-level variant filtering prior to simulation. Activating this mode requires calling “-vcf” and specifying a VCF file path after the “-vcf_file.” BVSim builds this mode to receive population-level SVs files such as gnomAD v4 [28]. Users can input selection criteria to obtain a table recording population-specific variants (e.g., “AF_fin $>$0.001” for Finnish allele frequency larger than 0.001). See Supplementary Tables S5– S7 for a summary of population codes and annotation rules of the gnomAD VCF file. The resulting SV table can be input into the exact mode for sequence generation after excluding overlapping SVs.

### Input validation

To ensure data integrity, BVSim automatically validates all input tables against format requirements before simulation. This validation process checks for proper field completion, coordinate validity, and logical consistency between variant types and their required parameters. Example tables demonstrating the correct format are provided in the GitHub repository, showing both basic and complex variant configurations.

### Output format for sequence simulation modes

BVSim outputs the simulated pseudo-genomic sequence in a standard FASTA format, compatible with read generators like PBSIM2 [29]. It also generates a table detailing variation positions, aiding in distinguishing true variations from sequencing errors. For introduced mutations in the VCF file (version 4.3), BVSim includes CSV-TYPE and CSV-INDEX fields in the INFO column, outlining CSV types and components.

**Figure 2:**
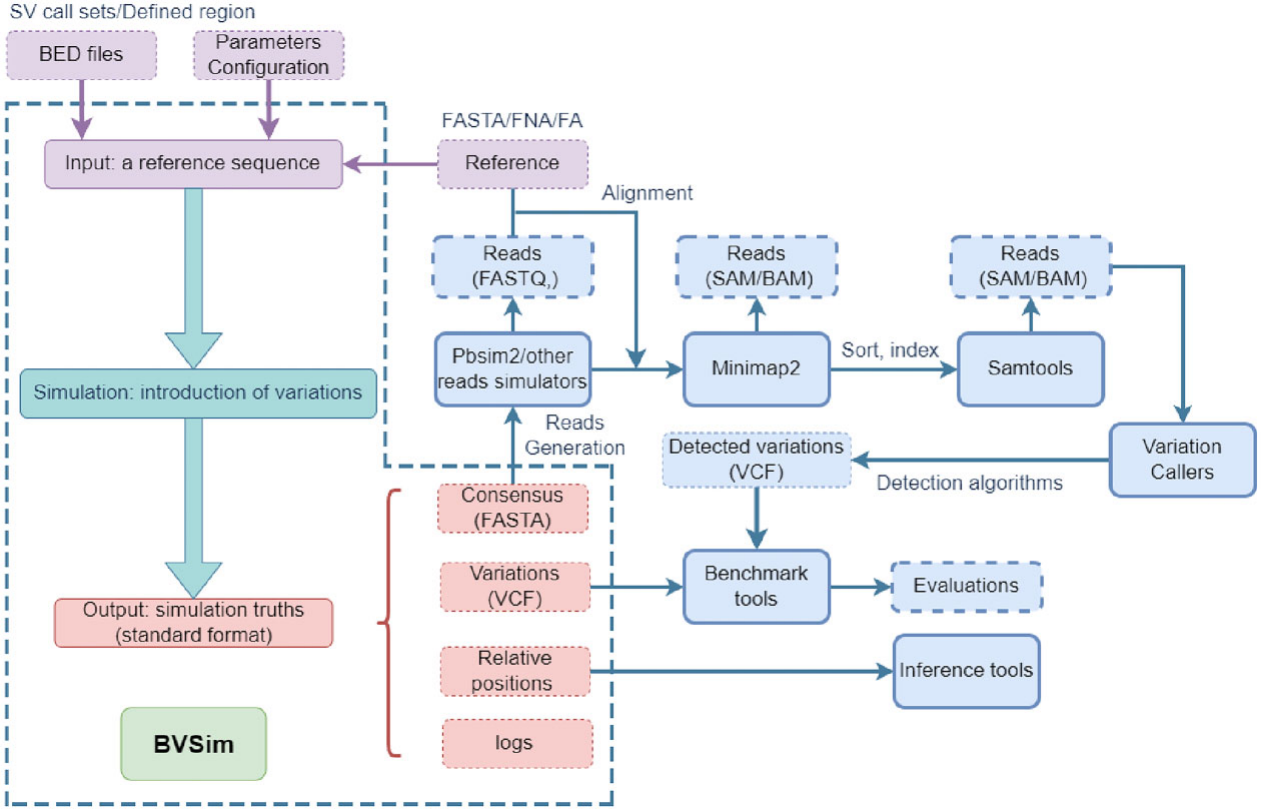
Workflow of BVSim’s sequence simulation and interactions with downstream analysis tools. The dashed box outlines BVSim’s core simulation module, with solid arrows indicating workflow progression. Files in purple boxes are user-provided inputs, while those in red boxes are outputs. Solid blue boxes represent third-party analysis tools, while dashed blue boxes contain their output files.

Figure 2 illustrates the workflow of BVSim, encapsulated within a dashed box, and demonstrates how the output files interact with read simulators, the alignment tool Minimap2 [30], SAMtools and BCFtools [31], and benchmark tools such as Truvari [32], SURVIVOR [14], vcfdist [33].

### Extension to polyploid and nonhuman genomes

BVSim’s framework supports simulations beyond diploid human genomes through flexible parameterization. For haploid references (e.g., microbial or viral genomes), users may directly simulate reads from any input sequence without modification. Polyploid systems (e.g., triploid or tetraploid organisms) can be modeled by processing homologous chromosomes sequentially: users may supply distinct variant profiles for each chromosome set and aggregate the results. The tool also accommodates somatic heterogeneity through user-defined variant tables with specified allele frequencies.

## Comparison with Existing Simulators

The primary goal is to develop an automated realistic variant simulator with controlled randomness to generate diverse synthesis data. Existing genome simulators demonstrate the following primary constraints. First, CSV simulation remains particularly underdeveloped, with current solutions like VISOR requiring laborious manual curation processes. Except for lacking a CSV simulation function, many existing variant simulators either only simulate SVs (e.g., Sim-it) or only simulate small variants, instead of trying to cover all types of genome variants. For those with both SVs and small variant simulation capability, empirical tools such as VarSim and RSVSim sample variants only from predetermined genomic regions. Lastly, parametric tools, including Simulome, simuG, SURVIVOR, and MTS, rely on oversimplified uniform distributions. BVSim overcomes these constraints through its innovative distribution learning framework, yielding a nonuniform sampling space, as shown in Supplementary Fig. S9d, which maintains biological fidelity while permitting users’ adjustments.

To illustrate the influence of different simulation mechanisms on downstream analyses, BVSim was compared against 2 representative simulation paradigms: the purely empirical approach (VarSim) and the purely random approach (MTS). The selection of MTS as the parametric representative was based on its relative flexibility compared to Simulome, simuG, and SURVIVOR, which share the same fundamental uniform sampling methodology but offer fewer user-configurable parameters. Several tools were excluded from quantitative comparison due to fundamental incompatibilities with automated benchmarking requirements. Sim-it’s inability to model small variants renders it unsuitable for comprehensive genomic simulation. VISOR’s manual curation requirements and RSVSim’s outdated annotation dependencies similarly preclude meaningful performance evaluation against BVSim’s automated framework.

We designed 2 benchmarking experiments to evaluate the effect of these methodological differences. The first experiment compared BVSim against VarSim using the hg19 reference genome, focusing on fixed region sampling. The second experiment contrasted BVSim with MTS using the hg38 reference genome to examine differences between realistic and uniform simulation approaches. Both experiments generated consistent variant sets comprising 1,000 simple SVs distributed across 50 chromosome 21 sequence pairs, with carefully matched variant type distributions and length ranges.

For downstream effect evaluation, we employed 5 established SV detection tools: PBSV (version 2.9.0) [34], Sniffles (version 2.2) [35], SVIM (version 2.0.0) [36], CuteSV (version 2.0.3) [37], and DeBreak (version 1.0.2) [38]. The “truvari bench” command from Truvari (version 4.1.0) [32] was used to benchmark the detection results and calculate F1 scores.

As shown in Supplementary Fig. S11 and Supplementary Tables S8–S9, VarSim and MTS exhibit systematic biases in their length distributions: VarSim produces right-skewed distributions with extreme outliers, while MTS generates uniformly distributed variants within fixed size ranges. In contrast, BVSim maintains naturally distributed variant lengths that agree better with biological observations. This systematic bias of VarSim and MTS may mislead performance evaluation for variant callers. This claim is supported by the observed F1 score differences summarized in Fig. 1B, C, which are significantly different from zero. The original F1 scores are shown in Supplementary Fig. S10.

Our controlled experiments reveal 2 key advantages of BVSim’s approach:

- **Precision in length distribution**: While maintaining the same potential length ranges as other simulators, BVSim naturally generates shorter SVs with left-skewed distributions that better match empirical length distributions, as presented in Supplementary Fig. S6.
- **Flexible control**: The framework has a more realistic default setting, but it can also provide simulations according to users’ requests.

These results collectively validate that BVSim’s probabilistic sampling framework produces more realistic variant profiles than existing simulation approaches, while maintaining greater flexibility in parameter control. This property is important for correct downstream method evaluation.

The comparison of downstream results shows that the above distribution difference between VarSim/MTS simulation and the real distribution will bias downstream conclusions. The unrealistically distributed variants generated from VarSim or MTS will lead to misleading variant caller performance, because the variant caller’s performance on the simulated data from VarSim or MTS will be different from its performance on the real human genome, which is successfully mimicked by our BVSim. Thus, we shall choose a simulator that produces more realistic sequences, which is exactly what BVSim is designed for. In support of this, we provide a visualization of 10 realizations of the simulations for benchmark datasets in Supplementary Fig. S12, where BVSim aligns with real genomic complexities, in contrast to the uniform distribution produced by the MTS.

## Discussion

BVSim is a Python-based tool that offers a comprehensive solution for simulating realistic genomic variations, accommodating SNPs, small indels, and simple and complex SVs. It provides flexibility in the distribution of variations, supports parallel processing for efficiency, and allows user-defined parameters, making it a versatile tool for evaluating variation calling tools and facilitating genomic research. The performance characteristics of the tool, including runtime metrics on different genomic scales, are detailed in Supplementary Tables S10– S12.

The tool’s reference genome compatibility extends beyond standard human assemblies to include the complete telomere-to-telomere (T2T) CHM13 genome [39]. While the benchmarking SV calling datasets for T2T CHM13 remain under development, BVSim enables forward-looking research by supporting both random and empirically modeled variation generation on this complete genomic template.

While offering these capabilities, BVSim does have several limitations that guide future development priorities. The current single-sequence processing architecture, while enabling efficient parallel computation and polyploid simulation, prevents modeling of interchromosomal events like translocations. The tool’s variant representation is also constrained by the completeness of input benchmark sets, where reliance on resources like GIAB v0.6 [9] may perpetuate biases against complex or repeat-associated variants. Additionally, while BVSim can model various ploidy levels through manual configuration, its utility for population-scale studies would benefit from specialized optimization.

These limitations directly inform BVSim’s development roadmap. We plan to enhance computational efficiency through optimized C/C++ implementation while expanding biological scope to include family-based simulations and automated species-specific parameterization. Particular attention will be given to agriculturally important polyploids and the integration of emerging community resources like the testHG008curation. By improving variant spectrum coverage through more comprehensive benchmark sets, future versions will address current gaps in SV representation while maintaining the tool’s modularity and cross-species applicability.

## Availability of Source Code and Requirements

- Project name: BVSim version 1.0.0
- Project homepage: https://github.com/YongyiLuo98/BVSim
- License: GPL-3.0
- SciCrunch RRID: SCR_026926
- bio.tools ID: BVSim
- WorkflowHub DOI [40]: https://doi.org/10.48546/WORKFLOWHUB.WORKFLOW.1361.1
- Configuration templates:
  – Default parameters: bvsim_config.yaml
  – User-configurable parameters: custom_config.yaml
  – Preconfigured scenarios: example shell scripts

### System requirements

- Operating system: Linux
- Programming language: Python 3.6 or higher
- Package management: Conda (environment.yml provided)
- Hardware requirements:
  – Minimum 4 GB RAM (8 GB+ recommended for large datasets)
  – Multicore CPU recommended for parallel processing
  – Adequate disk space for reference genomes and output files

## Additional Files

**Supplementary Fig. S1**. SV distribution across chromosomes (HG002) for chromosomes 1 to 22.

**Supplementary Fig. S2**. Mean SV counts and 95% confidence intervals across chromosomes (15 cell samples) for chromosomes 1 to 22.

**Supplementary Fig. S3**. Mean SV counts and 95% confidence intervals across chromosomes (32 HGSVC samples) for chromosomes 1 to 22.

**Supplementary Fig. S4**. Distribution of insertions and deletions in tandem repeat and non–tandem repeat regions in 15 cell samples and HG002 for chromosomes 1 to 22.

**Supplementary Fig. S5**. Distribution of insertions and deletions in tandem repeat and non–tandem repeat regions in 32 HGSVC samples for chromosomes 1 to 22.

**Supplementary Fig. S6**. The empirical length distribution of HG002, 15 cell samples and 32 HGSVC samples.

**Supplementary Fig. S7**. Comparison of deletion distribution generation methods on chromosome 21. The fixed option (a, c) directly uses observed counts from input samples, while the random option (b, d) redistributes variants according to bin-specific probabilities. Single-sample versus multisample comparisons demonstrate method consistency across datasets.

**Supplementary Fig. S8**. Simulated SV distributions in wave-region mode showing TR enrichment (chr21/hg38). Deletions and insertions demonstrate increased density in user-defined tandem repeat regions with a 500-kbp bin size.

**Supplementary Fig. S9**. The illustration of sampling probability spaces for different simulators using chromosome 10 as an example.

**Supplementary Fig. S10**. Boxplots showing the original F1 scores for variation detection algorithms on 50 datasets. (a) the F1 scores from BVSim datasets and those from VarSim (hg19). (b) The F1 scores from BVSim datasets and those from MTS (hg38).

**Supplementary Fig. S11**. Comparison of simulated SV length distributions on chr21. (a) BVSim vs. VarSim (hg19). (b) BVSim vs. MTS (hg38).

**Supplementary Fig. S12**. Comparison of simulated SVs generated by MTS and BVSim against benchmark datasets.

**Supplementary Fig. S13**. Dotplot comparison between the reference sequence (hg19 chr21:25,000,001–25,100,000) and simulated query sequence, annotated with SVs. Red dashed lines indicate SV positions labeled with their types (DEL/INS/DUP/INV).

**Supplementary Table S1**. Counts and proportions of deletions in tandem repeat regions across 15 cell samples.

**Supplementary Table S2**. Counts and proportions of insertions in tandem repeat regions across 15 cell samples.

**Supplementary Table S3**. Counts and proportions of deletions in tandem repeat regions across 32 HGSVC samples (transposed).

**Supplementary Table S4**. Counts and proportions of insertions in tandem repeat regions across 32 HGSVC samples (transposed).

**Supplementary Table S5**. Population codes and descriptions in gnomAD SV v4.1.

**Supplementary Table S6**. Key population-level SV annotation fields in gnomAD v4.1.

**Supplementary Table S7**. Genotype frequency metrics in gnomAD SV v4.1.

**Supplementary Table S8**. BVSim vs. VarSiM: simulated SV length statistics (hg19).

**Supplementary Table S9**. BVSim vs. MTS: simulated SV length statistics (hg38).

**Supplementary Table S10**. System hardware configuration for all replicates.

**Supplementary Table S11**. Runtime and footprint results for BVSim modes with 10 replicates.

**Supplementary Table S12**. Whole-genome runtime performance (hg38 and hg19).

giaf095_Supplemental_File_revised

giaf095_Authors_Response_To_Reviewer_Comments_original_submission

giaf095_Authors_Response_To_Reviewer_Comments_Revision_1

giaf095_GIGA-D-24-00483_original_submission

giaf095_GIGA-D-24-00483_Revision_1

giaf095_GIGA-D-24-00483_Revision_2

giaf095_Reviewer_1_Report_Original_Submission Nicolas Dierckxsens -- 11/29/2024

giaf095_Reviewer_2_Report_Original_Submission Sina Majidian -- 1/22/2025

giaf095_Reviewer_2_Report_Revision_1 Sina Majidian -- 5/18/2025

giaf095_Reviewer_3_Report_Original_Submission National Institute Technology -- 2/14/2025

giaf095_Reviewer_3_Report_Revision_1 National Institute Technology -- 6/4/2025

## Abbreviations

BED: browser extensible data; bp: base pairs; CSV: complex structural variation; DGV: database of genomic variants; GIAB: Genome in a Bottle; HGSVC: Human Genome Structural Variation Consortium; indels: insertions and deletions; MTS: Mutation-Simulator; SNP: single-nucleotide polymorphism; SV: structural variation; TR: tandem repeat; T2T: telomere-to-telomere; VCF: variant call format.

## Data Availability

The GRCh37 (hg19) and GRCh38 (hg38) human reference genomes can be found in the NCBI Assembly database [41] with accession numbers GCA_000001405.1 and GCA_000001405.29, respectively. The T2T-CHM13 v2.0 genome is also available at NCBI (GCA_009914755.4) [39]. The dbSNP dataset is available through the NCBI database [27, 42]. The DGV dataset is available at the DGV homepage. Variant calling results for HG002 are available at HG002_NA24385_son. The HGSVC dataset is available at HGSVC2 v1.0. The official documentation and download access of gnomAD are at gnomAD v4. Additionally, details about the 15 cell samples can be found in Supplementary Table S1 of the accompanying paper [6]. The tandem repeat regions are published by NCBI and can be retrieved from the following files: hg19.simpleRepeat.bed.gz for GRCh37 and hg38.repeats.bed.gz for GRCh38. The community reported SVs and CSVs can be found at the GitHub homepage: testHG008curation. Other data further supporting this work are openly available in the *GigaScience* repository, GigaDB [43].
