# Supplementary material for: BVSim: A benchmarking variation simulator mimicking human variation spectrum
Source: Gigascience. 2025 Aug 30;14:giaf095. doi: 10.1093/gigascience/giaf095 (PMC12398280; doi:10.1093/gigascience/giaf095)
Supplement: giaf095_Supplemental_File_revised [file giaf095_supplemental_file_revised.pdf]

Supplementary Information
BVSim: A Benchmarking Variation Simulator Mimicking Human Variation Spectrum

Yongyi Luo†, Zhen Zhang†, Jiandong Shi, Jingyu Hao, Sheng Lian, Taobo Hu, Toyotaka Ishibashi, Depeng Wang, Shu Wang*, Weichuan Yu* and Xiaodan Fan*

May 17, 2025

Genomic variations, including single nucleotide polymorphisms (SNPs), small insertions and deletions (indels), and structural variations (SVs), are crucial for understanding evolution and disease. However, comprehensive simulation tools for benchmarking genomic analysis methods are lacking. Existing simulators do not accurately represent the non-uniform distribution and length patterns of SVs in human genomes, and simulating complex structural variations (CSVs) remains challenging. To address these issues, we developed BVSim, a probabilistic simulation tool that effectively mimics the distributions of SVs, CSVs, SNPs, and small indels, enhancing benchmarking of analysis methods.

Contents

1 Human SVs **4**
- 1.1 Individual-Level Benchmark Datasets . . . 4
- 1.2 Non-uniform distribution of SVs across chromosomes . . . 4
  - 1.2.1 HG002 . . . 4
  - 1.2.2 15 *Cell* Samples . . . 10
  - 1.2.3 32 *HGSVC* Samples . . . 16
- 1.3 Higher rates of SVs in TR regions . . . 22
  - 1.3.1 15 *Cell* Samples and HG002 . . . 22
  - 1.3.2 32 *HGSVC* Samples . . . 28
- 1.4 Non-uniform distribution in SV lengths . . . 34
- 1.5 Population-Level SV Annotations of gnomAD v4.1 . . . 35

2 Introduction to BVSim **36**
- 2.1 Exact/uniform/uniform-parallel . . . 36
- 2.2 CSV Definitions . . . 36
- 2.3 Mimic/wave/wave-region: empirical vs probability . . . 37
- 2.4 Implementation Details for Sampling . . . 37
  - 2.4.1 Position Sampling . . . 37
  - 2.4.2 Length Sampling . . . 37
  - 2.4.3 Parallel Execution . . . 37
- 2.5 Comparison with VarSim and Mutation-Simulator (MTS) . . . 39
  - 2.5.1 Methodological Differences in SV Simulation Approaches . . . 39
  - 2.5.2 Comparative Analysis of SV Length Distributions Across Simulated Data . . . 40
  - 2.5.3 Visualization of SV Distribution Patterns: BVSim vs MTS . . . 42
- 2.6 Runtime Results Across Different Simulation Modes . . . 43
- 2.7 Dotplot with Annotated SVs . . . 45

References **46**

List of Figures

S1 SV distribution across chromosomes (HG002) for chromosomes 1 and 2. 4
S1 SV distribution across chromosomes (HG002) for chromosomes 3 to 6. 5
S1 SV distribution across chromosomes (HG002) for chromosomes 7 to 10. 6
S1 SV distribution across chromosomes (HG002) for chromosomes 11 to 14. 7
S1 SV distribution across chromosomes (HG002) for chromosomes 15 to 18. 8
S1 SV distribution across chromosomes (HG002) for chromosomes 19 to 22. 9
S2 Mean SV counts and 95% confidence intervals across chromosomes (15 *Cell* Samples) for chromosomes 1 to 3. 10
S2 Mean SV counts and 95% confidence intervals across chromosomes (15 *Cell* Samples) for chromosomes 4 to 7. 11
S2 Mean SV counts and 95% confidence intervals across chromosomes (15 *Cell* Samples) for chromosomes 8 to 11. 12
S2 Mean SV counts and 95% confidence intervals across chromosomes (15 *Cell* Samples) for chromosomes 12 to 15. 13
S2 Mean SV counts and 95% confidence intervals across chromosomes (15 *Cell* Samples) for chromosomes 16 to 19. 14
S2 Mean SV counts and 95% confidence intervals across chromosomes (15 *Cell* Samples) for chromosomes 20 to 22. 15
S3 Mean SV counts and 95% confidence intervals across chromosomes (32 *HGSVC* Samples) for chromosomes 1 to 3. 16
S3 Mean SV counts and 95% confidence intervals across chromosomes (32 *HGSVC* Samples) for chromosomes 4 to 7. 17
S3 Mean SV counts and 95% confidence intervals across chromosomes (32 *HGSVC* Samples) for chromosomes 8 to 11. 18
S3 Mean SV counts and 95% confidence intervals across chromosomes (32 *HGSVC* Samples) for chromosomes 12 to 15. 19
S3 Mean SV counts and 95% confidence intervals across chromosomes (32 *HGSVC* Samples) for chromosomes 16 to 19. 20
S3 Mean SV counts and 95% confidence intervals across chromosomes (32 *HGSVC* Samples) for chromosomes 20 to 22. 21
S4 Distribution of insertions and deletions in tandem repeat and non-tandem repeat regions in 15 *Cell* samples and HG002 for chromosome 1 to 4 22
S4 Distribution of insertions and deletions in tandem repeat and non-tandem repeat regions in 15 *Cell* samples and HG002 for chromosome 5 to 10 23
S4 Distribution of insertions and deletions in tandem repeat and non-tandem repeat regions in 15 *Cell* samples and HG002 for chromosome 11 to 16 24
S4 Distribution of insertions and deletions in tandem repeat and non-tandem repeat regions in 15 *Cell* samples and HG002 for chromosome 17 to 22 25
S5 Distribution of insertions and deletions in tandem repeat and non-tandem repeat regions in 32 *HGSVC* samples for chromosome 1 to 4 28
S5 Distribution of insertions and deletions in tandem repeat and non-tandem repeat regions in 32 *HGSVC* samples for chromosome 5 to 10 29
S5 Distribution of insertions and deletions in tandem repeat and non-tandem repeat regions in 32 *HGSVC* samples for chromosome 11 to 16 30
S5 Distribution of insertions and deletions in tandem repeat and non-tandem repeat regions in 32 *HGSVC* samples for chromosome 17 to 22 31
S6 The empirical length distribution of *HG002*, 15 *Cell* samples and 32 *HGSVC* samples . 34
S7 Comparison of deletion distribution generation methods on chromosome 21. Empirical mode (a,c) directly uses observed counts from input samples, while probability mode (b,d) redistributes variants according to segment-specific probabilities. Single-sample (a,b) versus multi-sample (c,d) comparisons demonstrate method consistency across datasets. 38
S8 Simulated SV distributions in wave-region mode showing TR enrichment (chr21/hg38). (a) Deletions and (b) insertions demonstrate increased density in user-defined tandem repeat regions with 500kbp bin size. 38

S9 The illustration of sampling probability spaces for different simulators using chromosome 10 as an example. 39
S10 Boxplots showing the original F1 scores for variation detection algorithms on 50 datasets.(a) the F1 scores from BVSim datasets and those from VarSim (hg19). (b) the F1 scores from BVSim datasets and those from MTS (hg38). 40
S11 Comparison of simulated SV length distributions on chr21.(a) BVSim vs VarSim (hg19). (b) BVSim vs MTS (hg38). 40
S12 Comparison of simulated SVs generated by MTS and BVSim against benchmark datasets 42
S13 Dotplot comparison between the reference sequence (hg19 chr21:25,000,001-25,100,000) and simulated query sequence, annotated with SVs. Red dashed lines indicate SV positions labeled with their types (DEL/INS/DUP/INV). 45

List of Tables

S1 Counts and proportions of deletions in tandem repeat regions across 15 *Cell* samples . . 26
S2 Counts and proportions of insertions in tandem repeat regions across 15 *Cell* samples . 27
S3 Counts and proportions of deletions in tandem repeat regions across 32 *HGSVC* samples (transposed) 32
S4 Counts and proportions of insertions in tandem repeat regions across 32 *HGSVC* samples (transposed) 33
S5 Population codes and descriptions in gnomAD SV v4.1 35
S6 Key population-level SV annotation fields in gnomAD v4.1 35
S7 Genotype frequency metrics in gnomAD SV v4.1 35
S8 BVSim vs VarSiM: simulated SV Length Statistics (hg19) 41
S9 BVSim vs MTS: simulated SV Length Statistics (hg38) 41
S10 System hardware configuration for all replicates 43
S11 Runtime and footprint results for BVSim modes with 10 replicates 43
S12 Whole-Genome Runtime Performance (hg38 and hg19) 44

1 Human SVs

1.1 Individual-Level Benchmark Datasets

For the GRCh38/hg38 reference genome, we utilize the 15 samples from the *Cell* study(Audano et al., 2019), hereafter referred to as the "15 *Cell* samples" and the 32 samples from the HGSVC dataset (Tan et al., 2024). For the GRCh37/hg19 reference genome, we refer to the HG002 sample (Zook et al., 2016). Our analysis of these datasets reveals three notable characteristics of human insertions and deletions, which align with findings from a large-scale study of Icelandic individuals(Beyter et al., 2021):

- SVs tend to occur non-uniformly across chromosomes, exhibiting higher rates near telomeres.
- SVs are more frequently observed within tandem repeat (TR) regions, despite these regions constituting a small proportion of the overall chromosome.
- The distributions of SV lengths are non-uniform, indicating variability in size.

1.2 Non-uniform distribution of SVs across chromosomes

Figure S1 presents the counts of deletions and insertions across the hg19 chromosomes, using a bin size of 500,000 base pairs (bp) for HG002 intervals, with connected data points illustrating trends in SV distribution. Figure S2/S3 shows data from the 15 *Cell* samples/ the 32 *HGSVC* samples aligned to hg38, depicting the mean SV count (blue line) and 95% confidence interval (green shading). Grey areas represent gaps in the reference genome (N bases), while red areas highlight centromere structures. This visualization effectively demonstrates the non-uniform distribution of SVs across chromosomes.

1.2.1 HG002

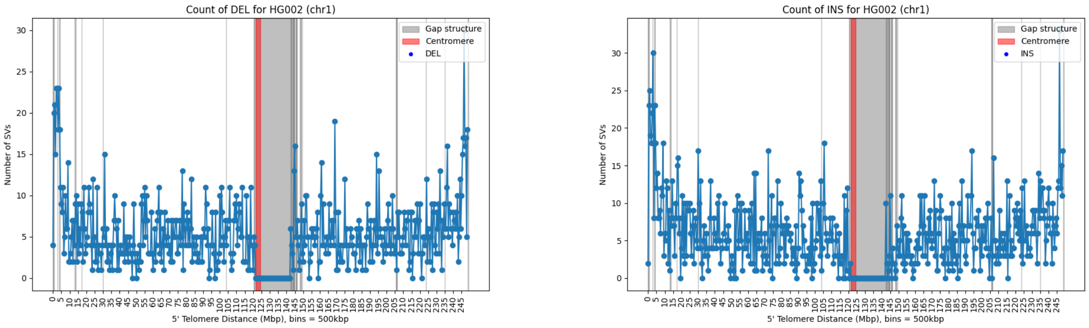

(a) Deletions and insertions for chromosome 1

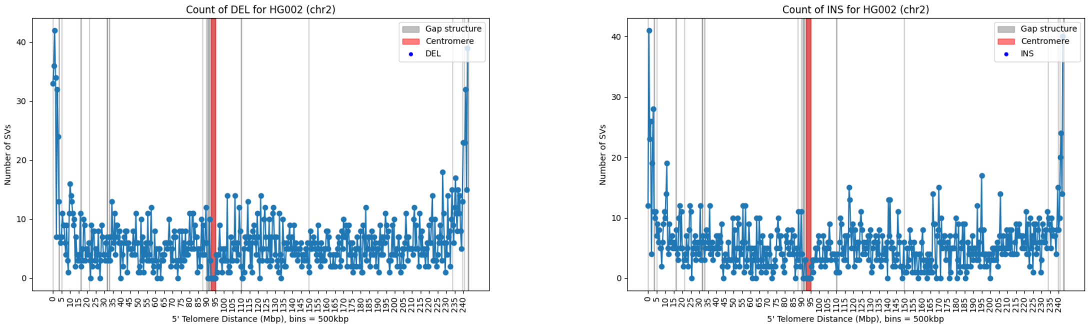

(b) Deletions and insertions for chromosome 2

Figure S1: SV distribution across chromosomes (HG002) for chromosomes 1 and 2.

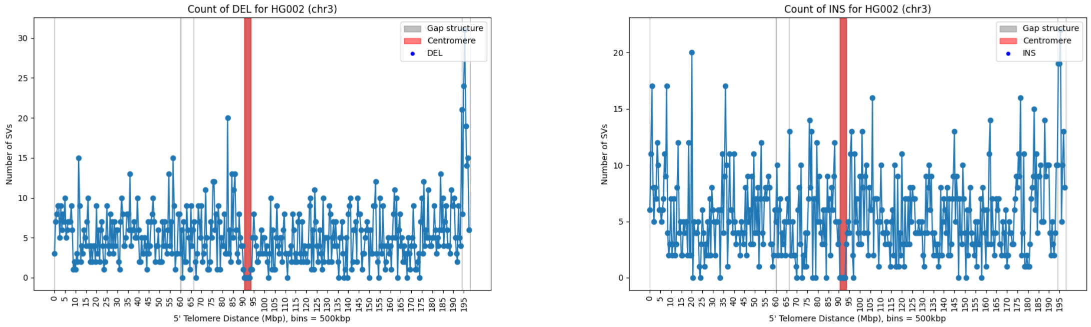

(c) Deletions and insertions for chromosome 3

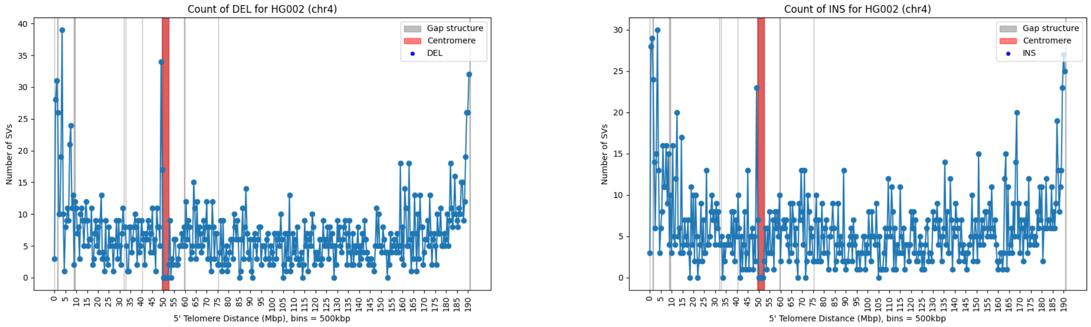

(d) Deletions and insertions for chromosome 4

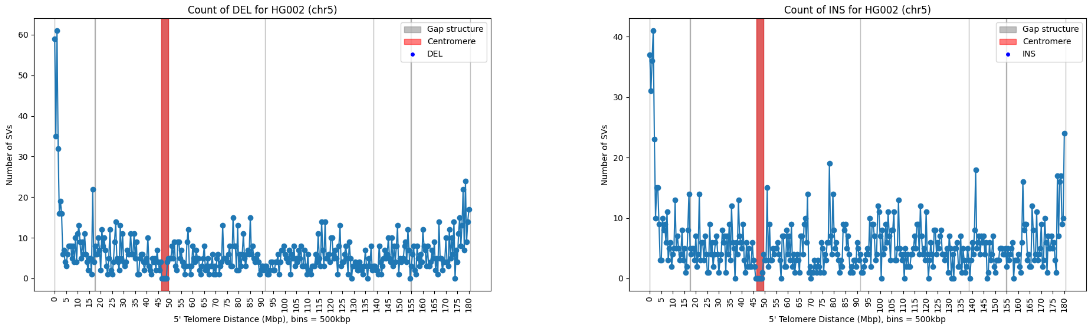

(e) Deletions and insertions for chromosome 5

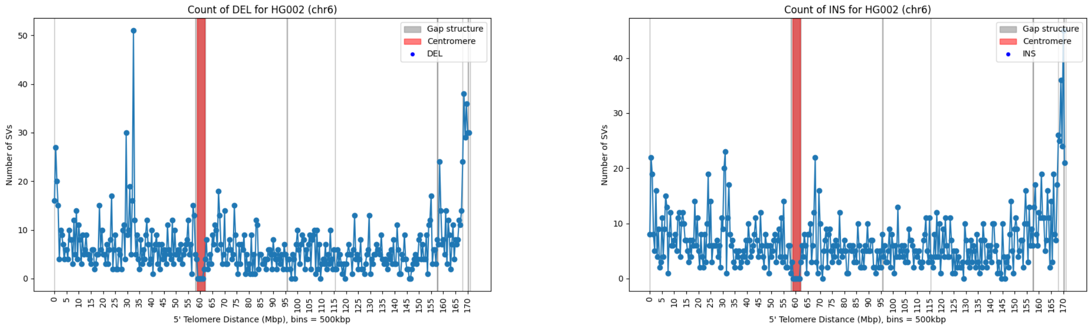

(f) Deletions and insertions for chromosome 6

Figure S1: SV distribution across chromosomes (HG002) for chromosomes 3 to 6.

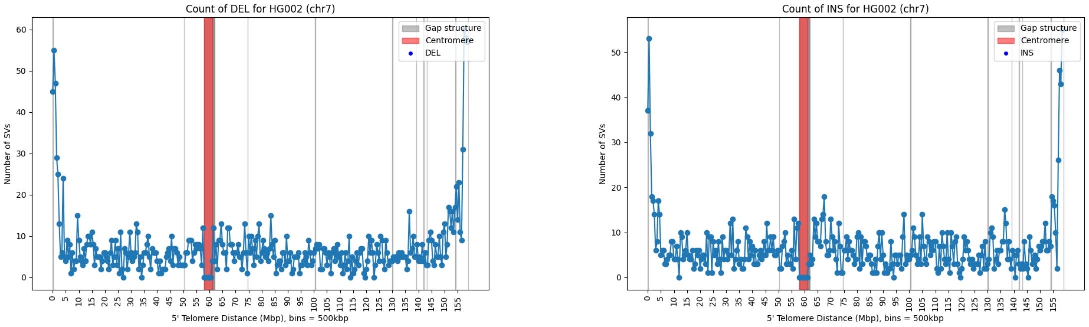

(g) Deletions and insertions for chromosome 7

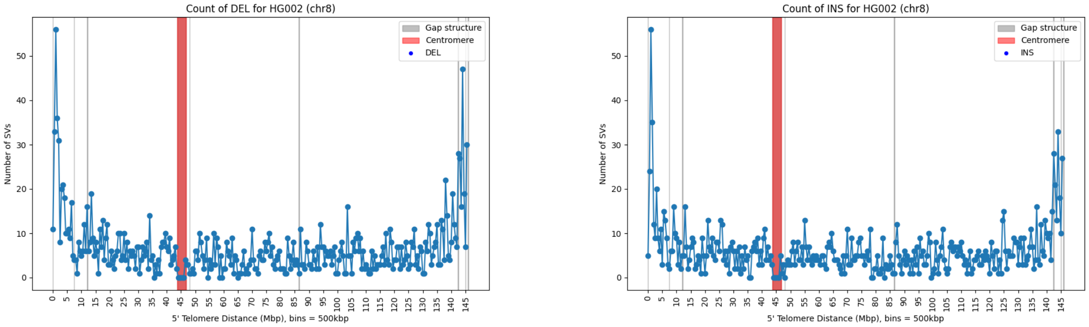

(h) Deletions and insertions for chromosome 8

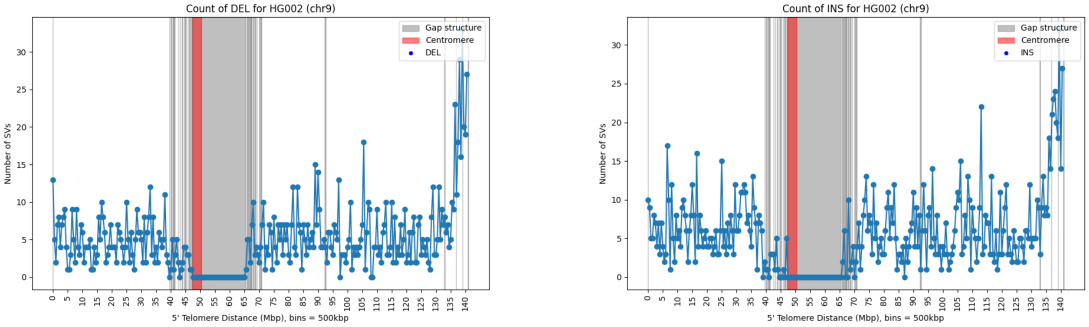

(i) Deletions and insertions for chromosome 9

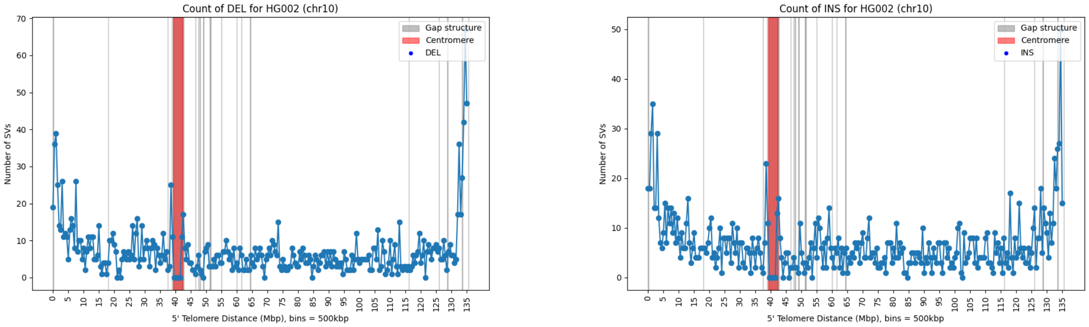

(j) Deletions and insertions for chromosome 10

Figure S1: SV distribution across chromosomes (HG002) for chromosomes 7 to 10.

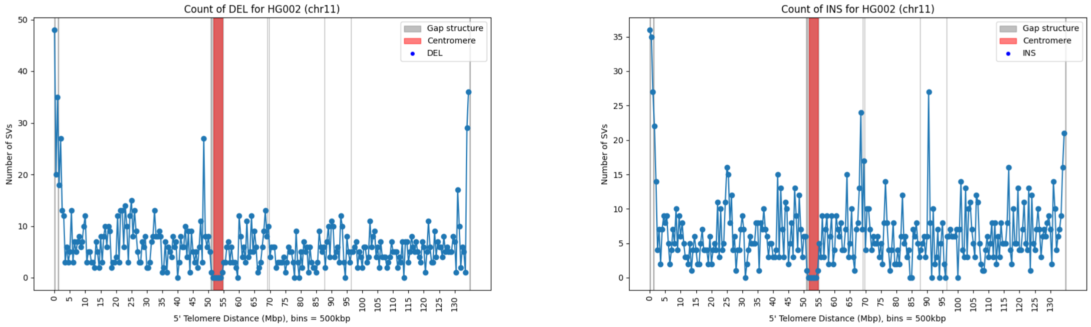

(k) Deletions and insertions for chromosome 11

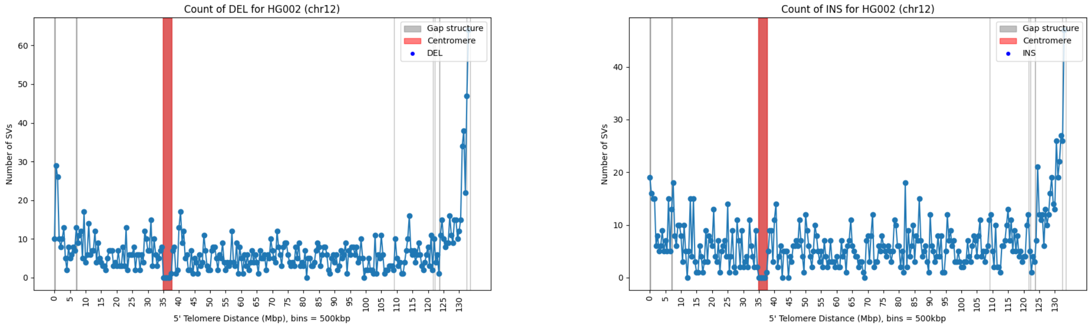

(l) Deletions and insertions for chromosome 12

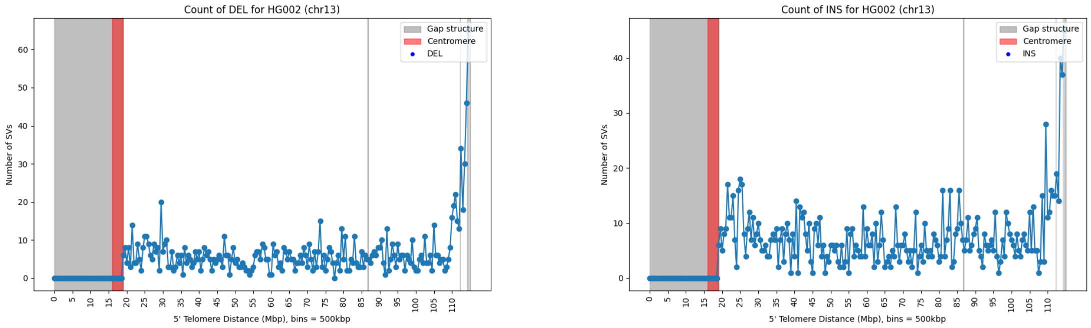

(m) Deletions and insertions for chromosome 13

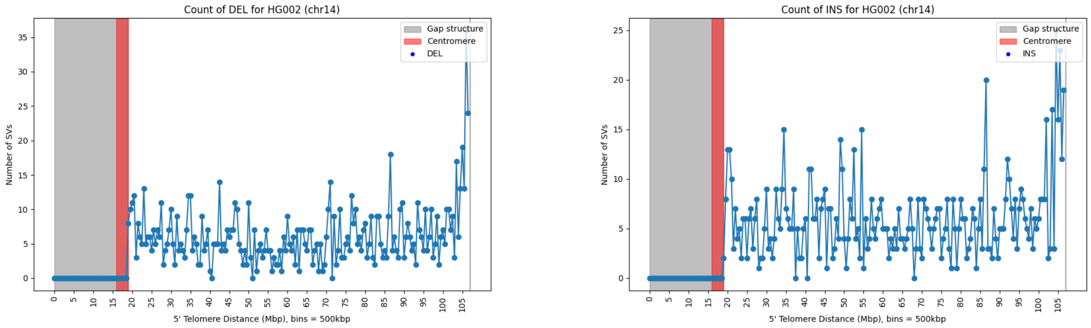

(n) Deletions and insertions for chromosome 14

Figure S1: SV distribution across chromosomes (HG002) for chromosomes 11 to 14.

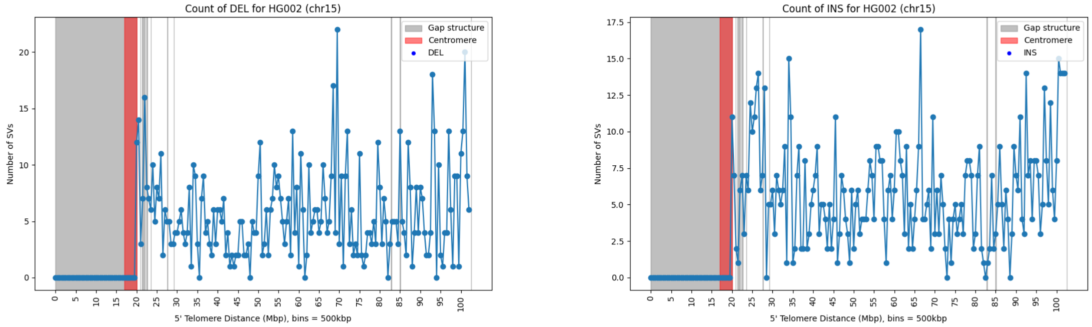

(o) Deletions and insertions for chromosome 15

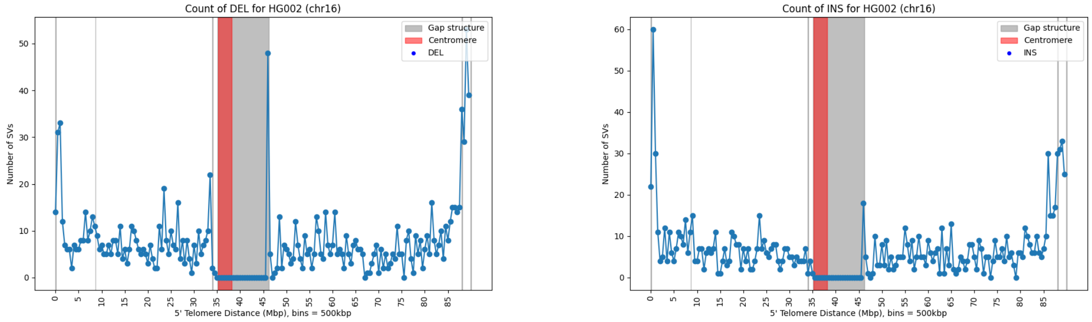

(p) Deletions and insertions for chromosome 16

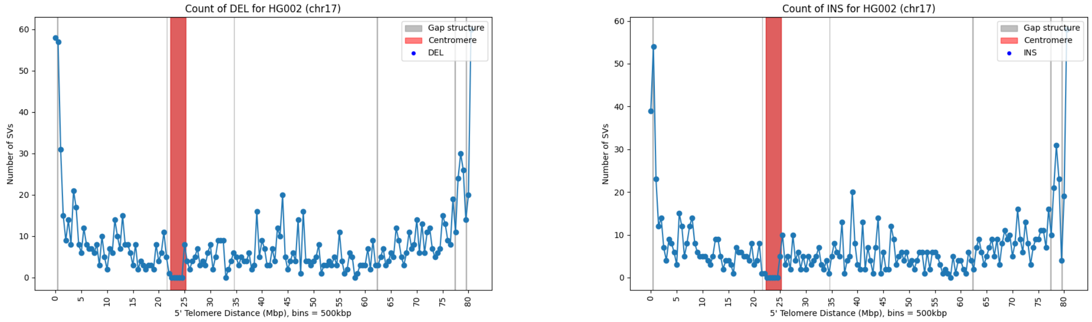

(q) Deletions and insertions for chromosome 17

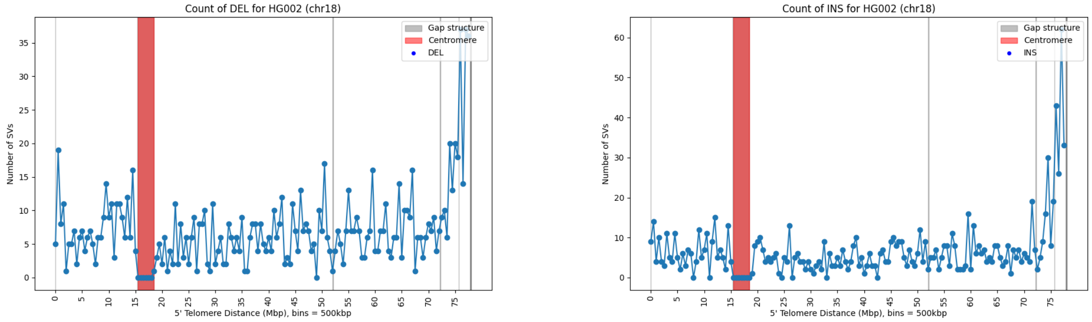

(r) Deletions and insertions for chromosome 18

Figure S1: SV distribution across chromosomes (HG002) for chromosomes 15 to 18.

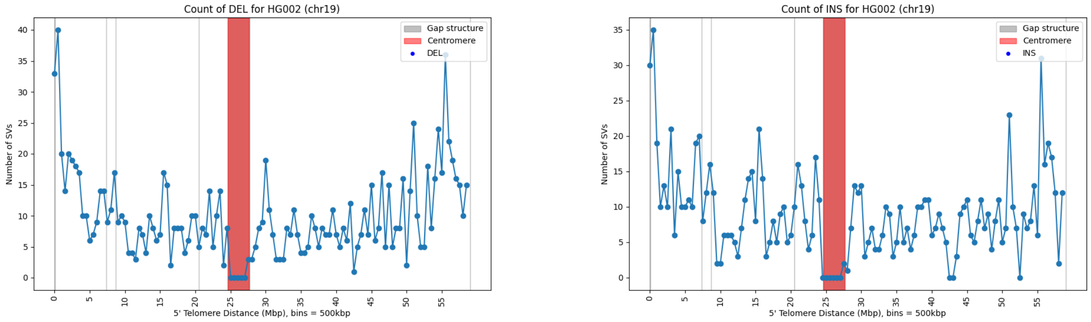

(s) Deletions and insertions for chromosome 19

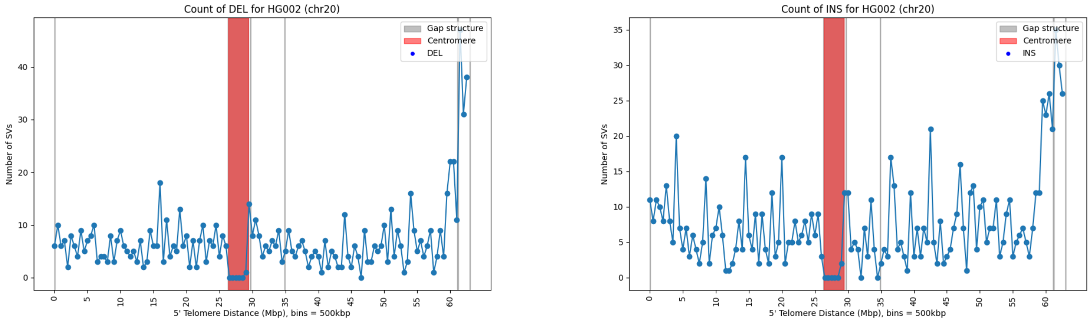

(t) Deletions and insertions for chromosome 20

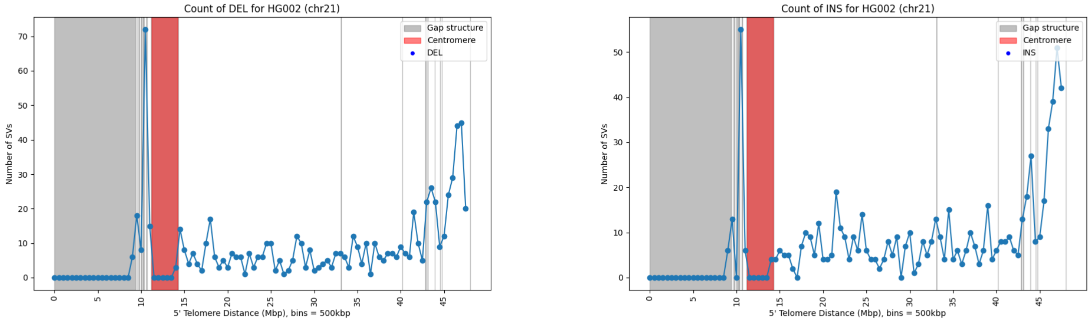

(u) Deletions and insertions for chromosome 21

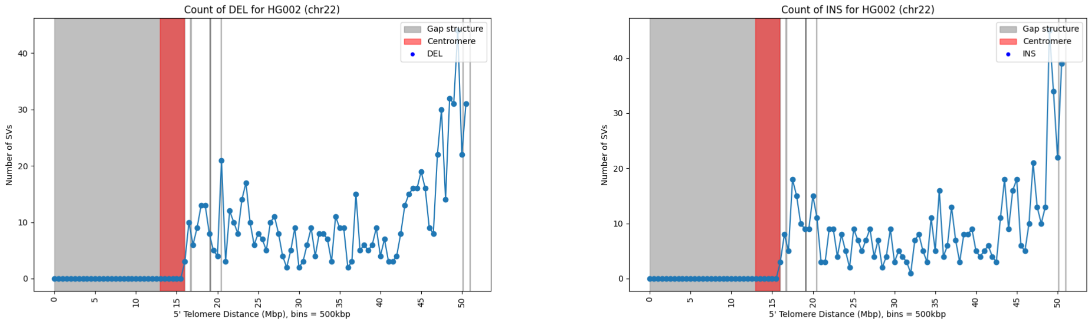

(v) Deletions and insertions for chromosome 22

Figure S1: SV distribution across chromosomes (HG002) for chromosomes 19 to 22.

1.2.2 15 *Cell* Samples

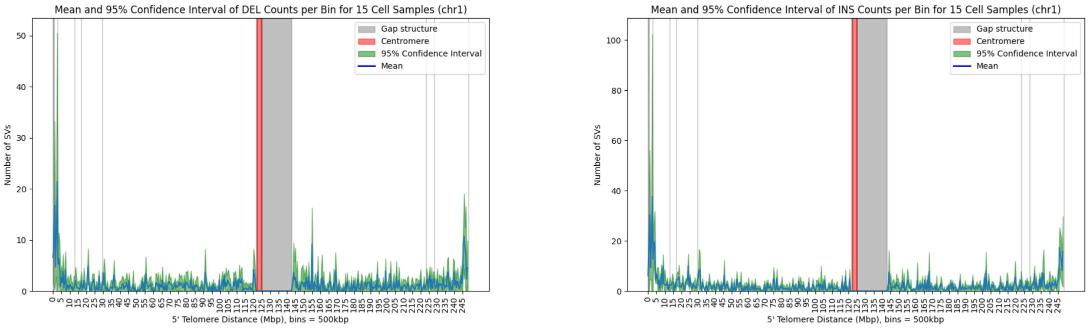

(a) Deletions and insertions for chromosome 1

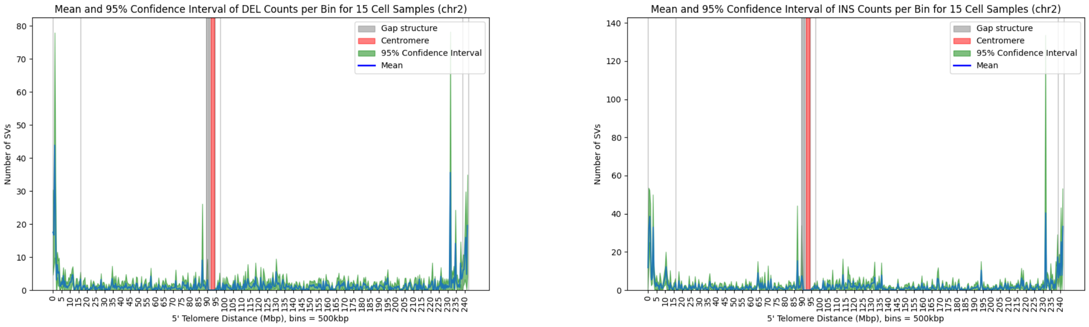

(b) Deletions and insertions for chromosome 2

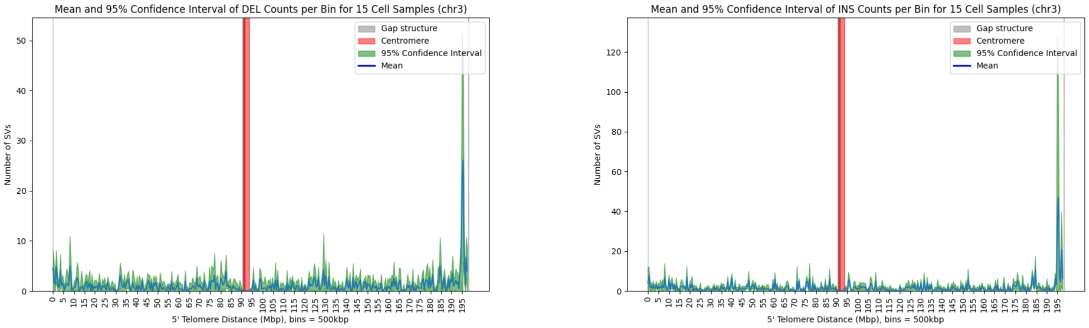

(c) Deletions and insertions for chromosome 3

Figure S2: Mean SV counts and 95% confidence intervals across chromosomes (15 *Cell* Samples) for chromosomes 1 to 3.

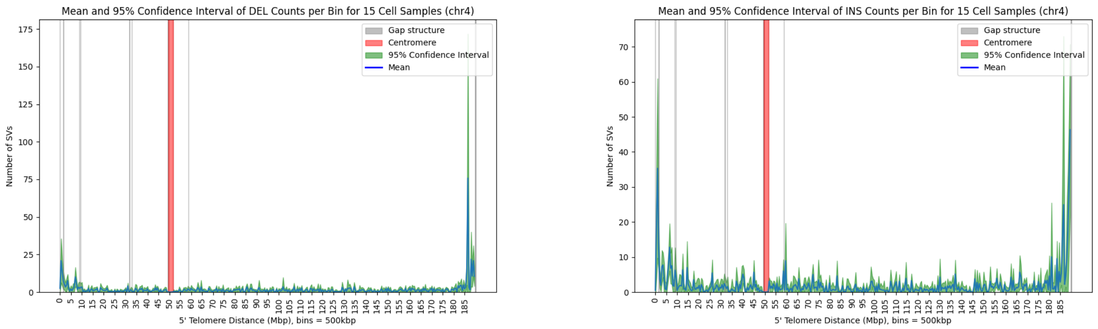

(d) Deletions and insertions for chromosome 4

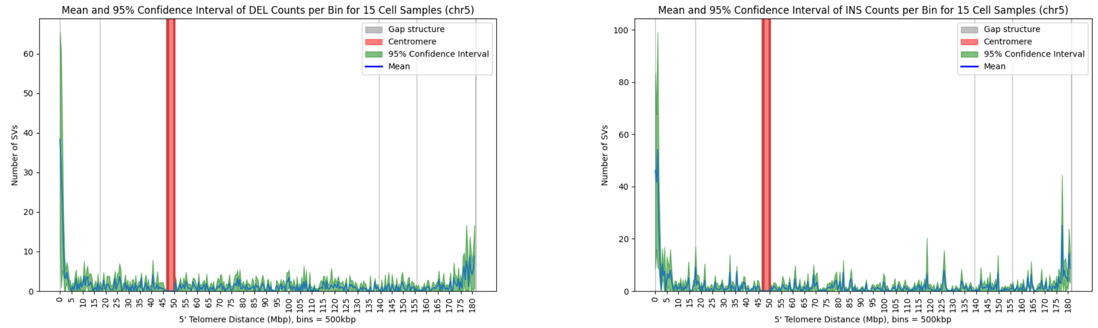

(e) Deletions and insertions for chromosome 5

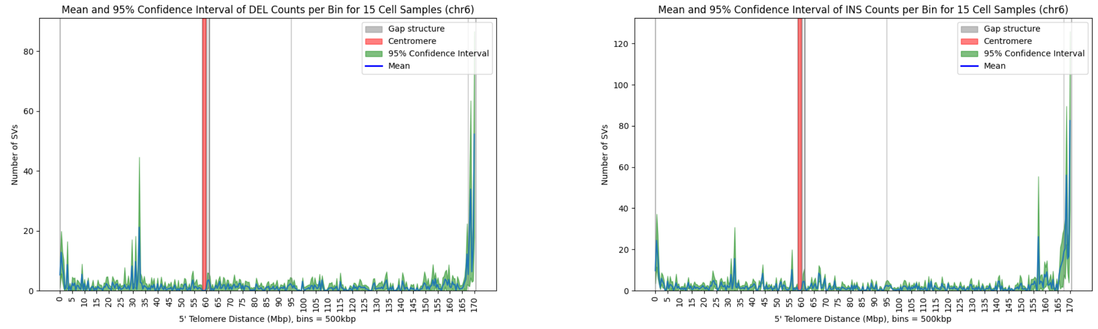

(f) Deletions and insertions for chromosome 6

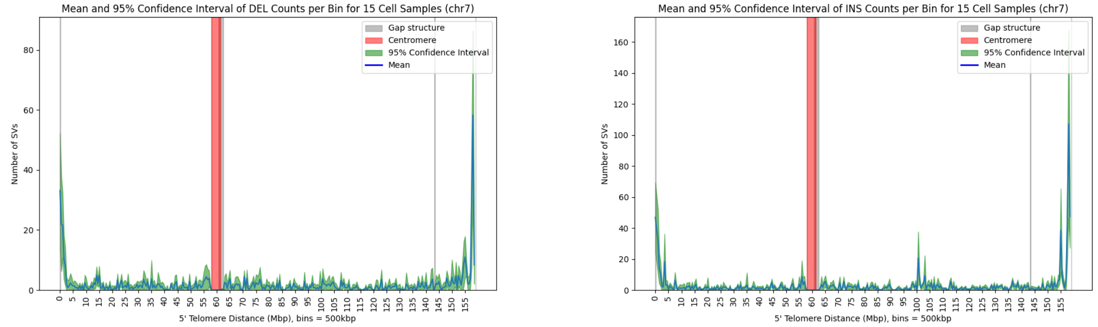

(g) Deletions and insertions for chromosome 7

Figure S2: Mean SV counts and 95% confidence intervals across chromosomes (15 *Cell* Samples) for chromosomes 4 to 7.

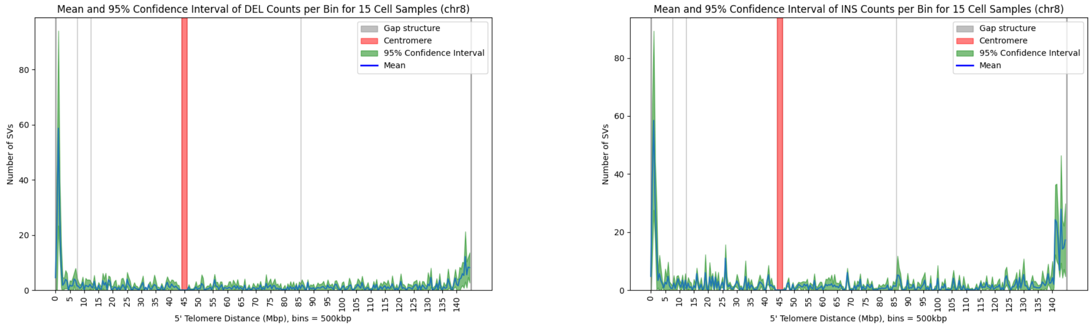

(h) Deletions and insertions for chromosome 8

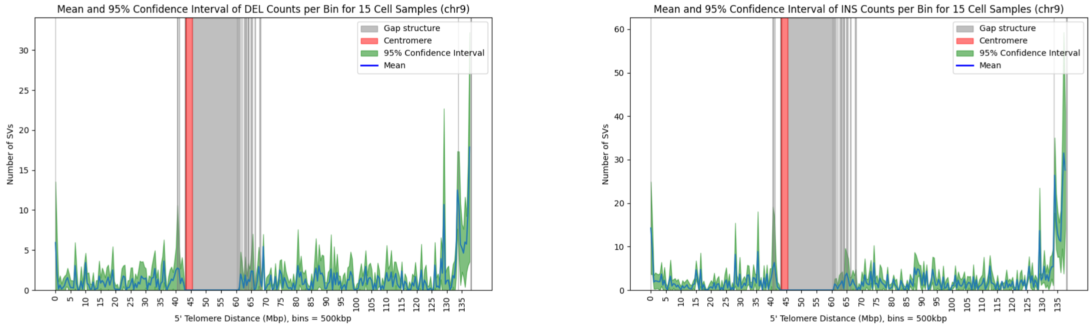

(i) Deletions and insertions for chromosome 9

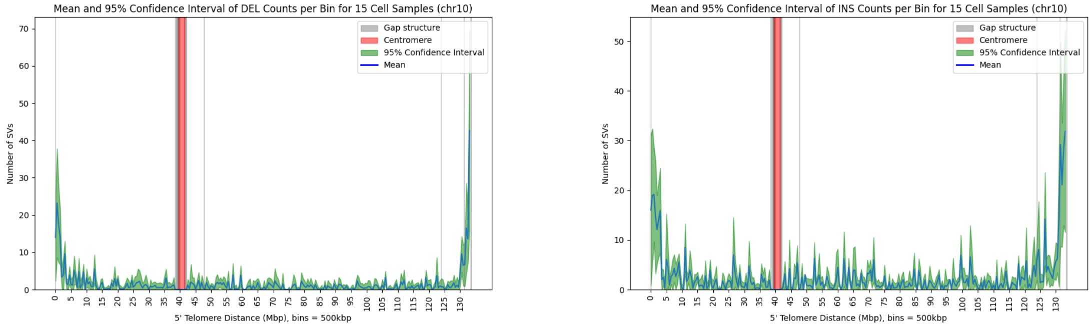

(j) Deletions and insertions for chromosome 10

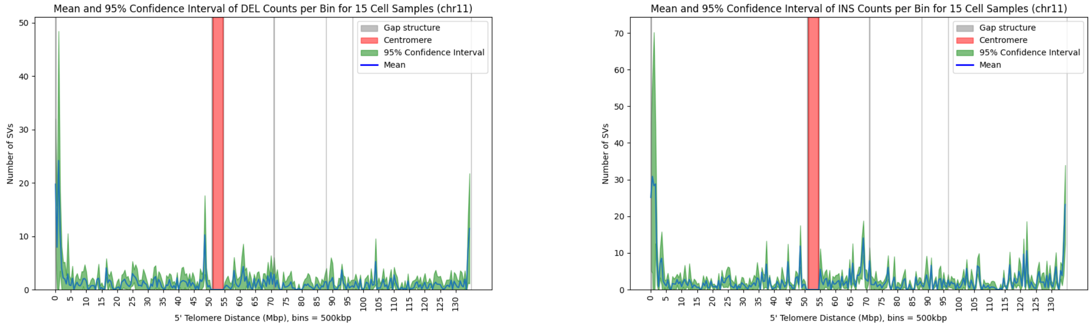

(k) Deletions and insertions for chromosome 11

Figure S2: Mean SV counts and 95% confidence intervals across chromosomes (15 *Cell* Samples) for chromosomes 8 to 11.

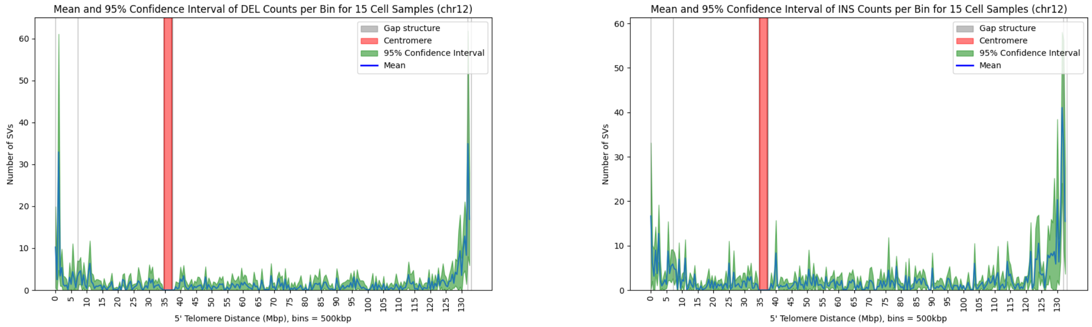

(l) Deletions and insertions for chromosome 12

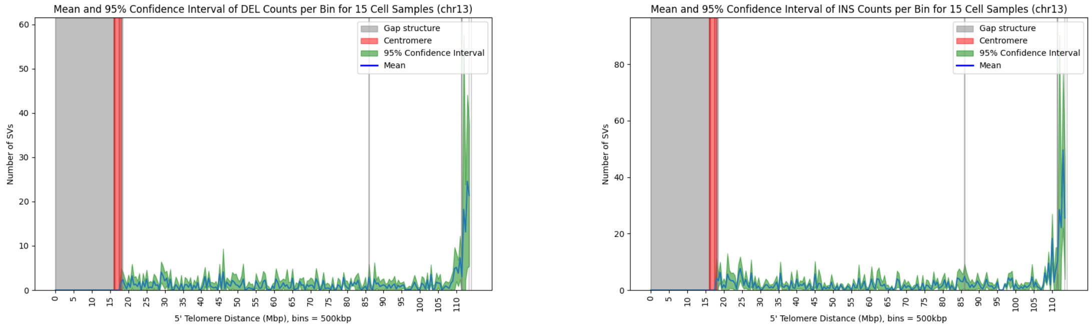

(m) Deletions and insertions for chromosome 13

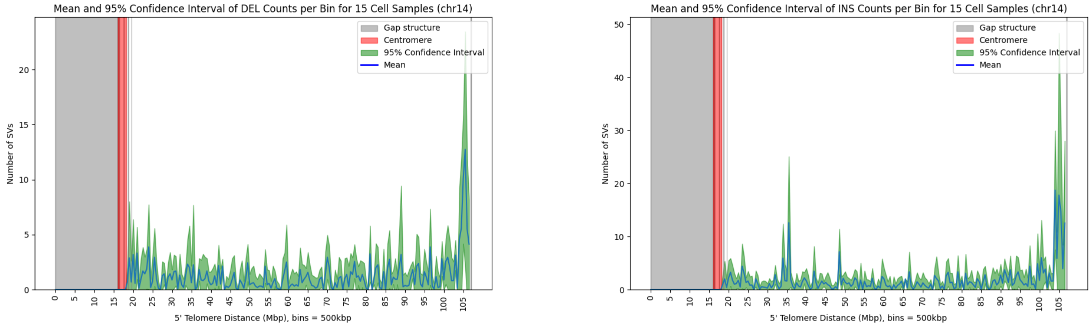

(n) Deletions and insertions for chromosome 14

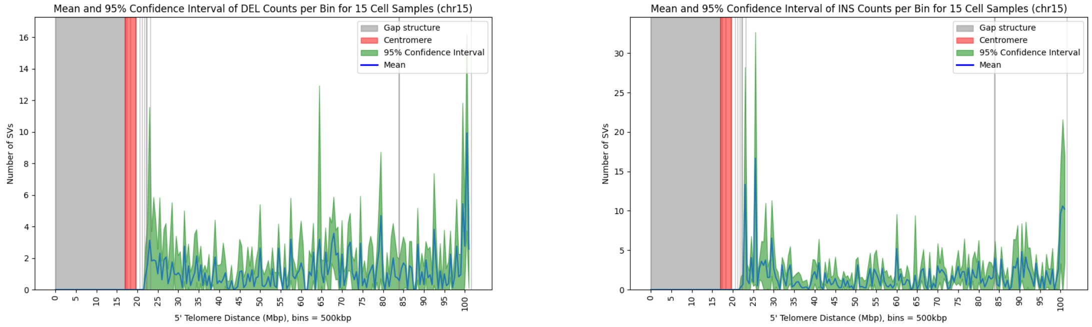

(o) Deletions and insertions for chromosome 15

Figure S2: Mean SV counts and 95% confidence intervals across chromosomes (15 *Cell* Samples) for chromosomes 12 to 15.

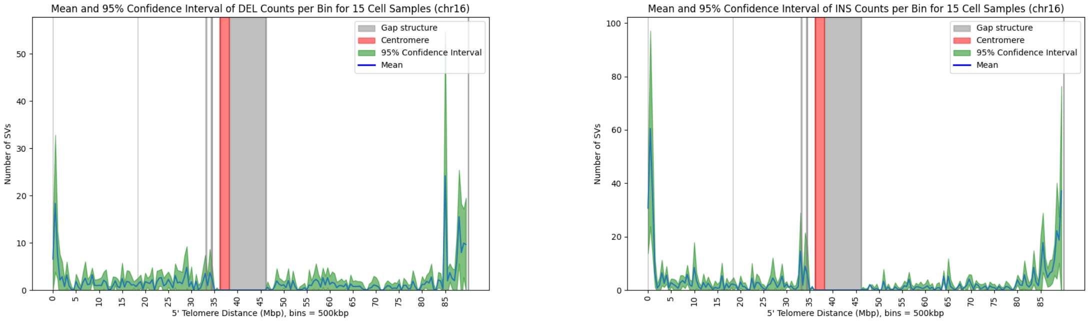

(p) Deletions and insertions for chromosome 16

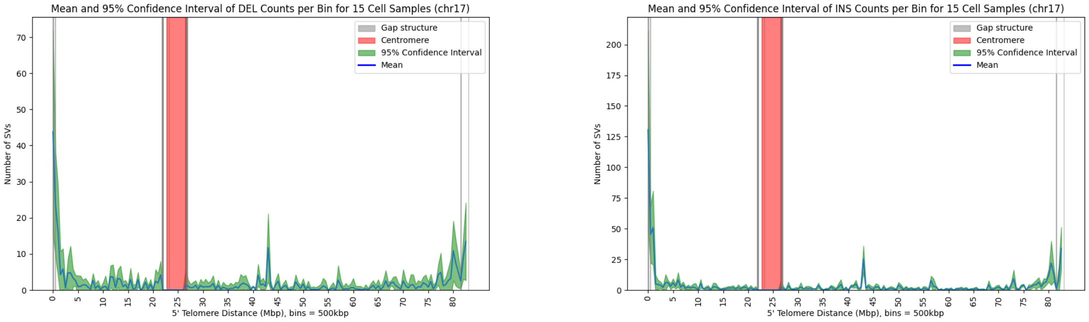

(q) Deletions and insertions for chromosome 17

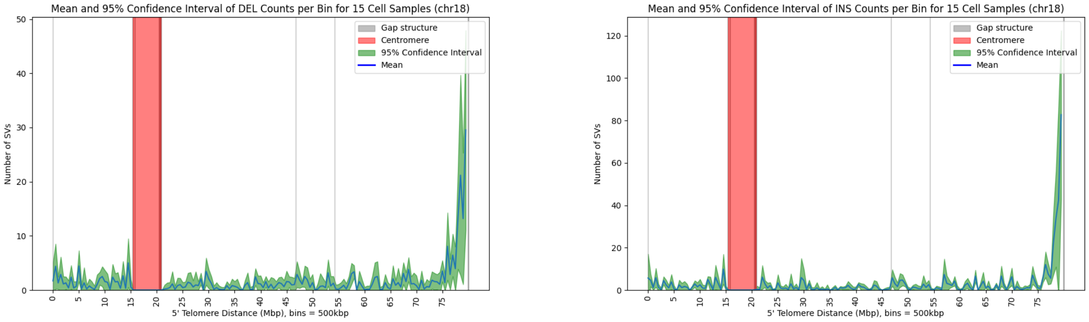

(r) Deletions and insertions for chromosome 18

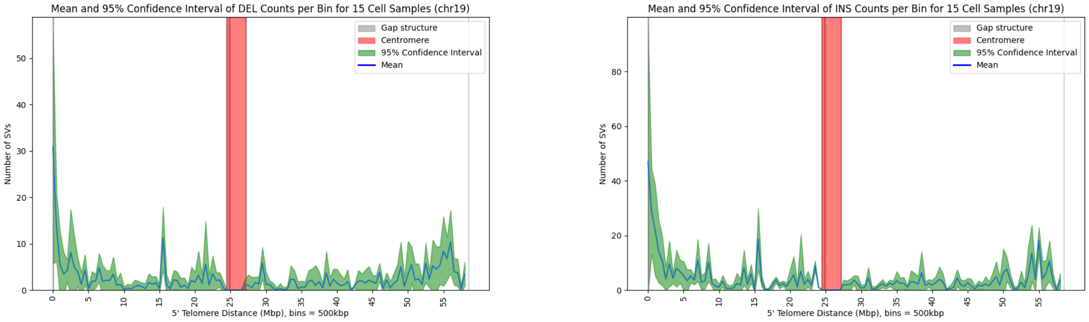

(s) Deletions and insertions for chromosome 19

Figure S2: Mean SV counts and 95% confidence intervals across chromosomes (15 *Cell* Samples) for chromosomes 16 to 19.

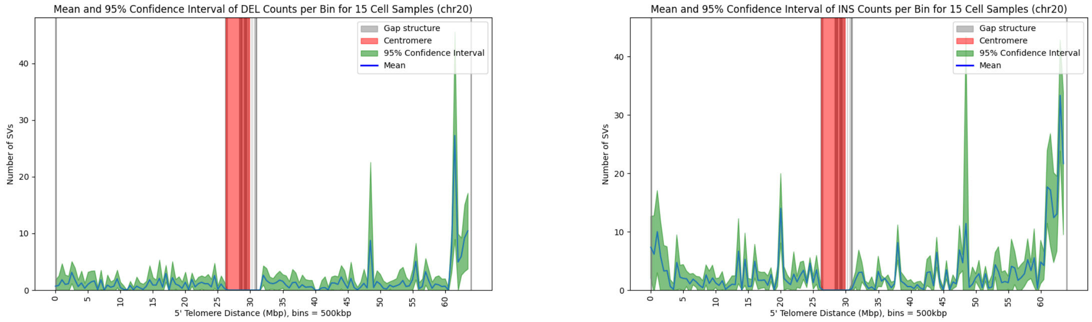

(t) Deletions and insertions for chromosome 20

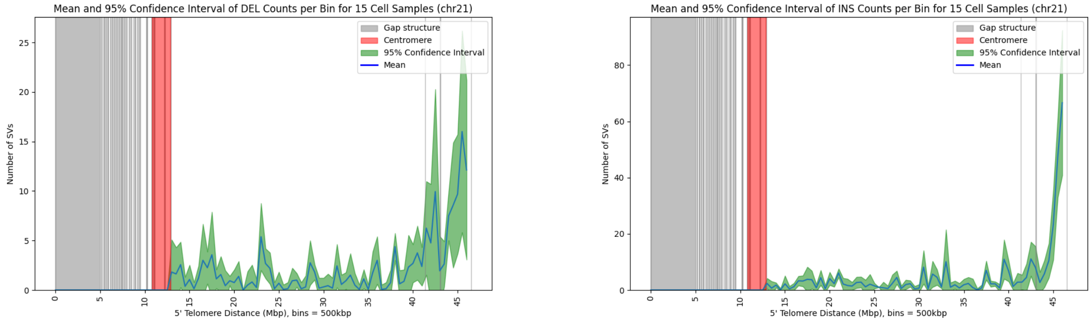

(u) Deletions and insertions for chromosome 21

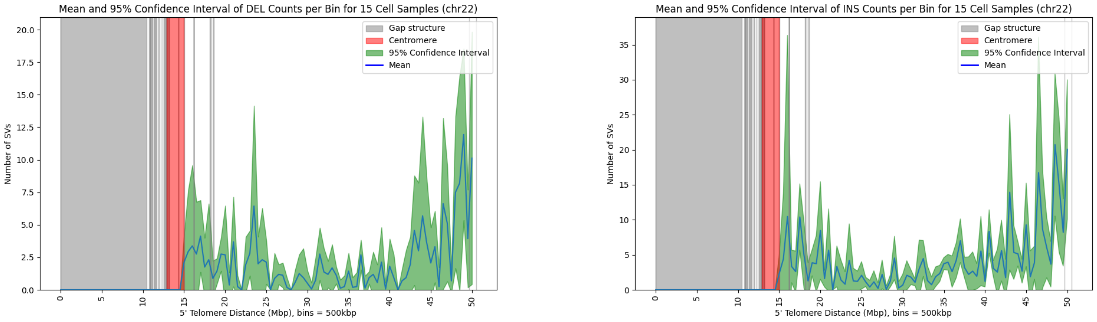

(v) Deletions and insertions for chromosome 22

Figure S2: Mean SV counts and 95% confidence intervals across chromosomes (15 *Cell* Samples) for chromosomes 20 to 22.

1.2.3 32 *HGSVC* Samples

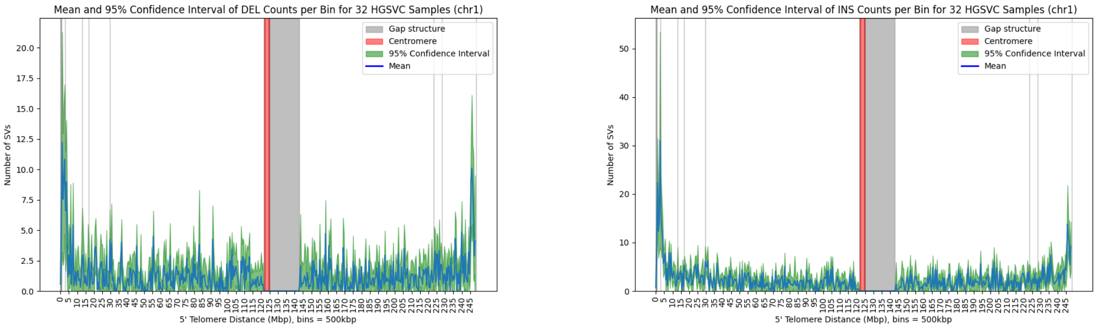

(a) Deletions and insertions for chromosome 1

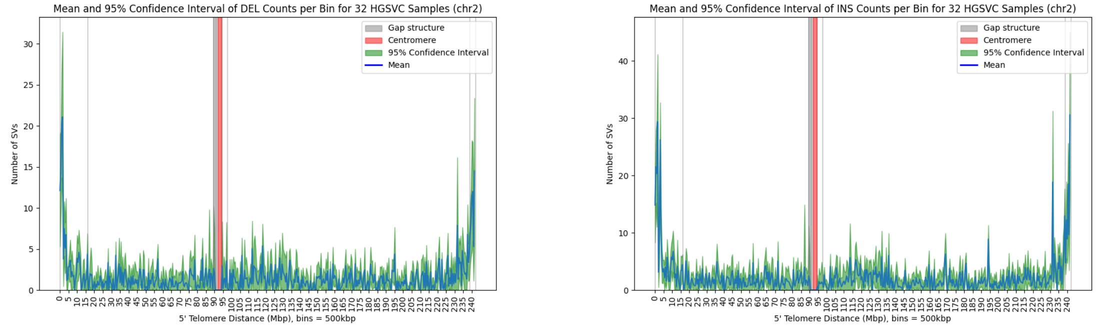

(b) Deletions and insertions for chromosome 2

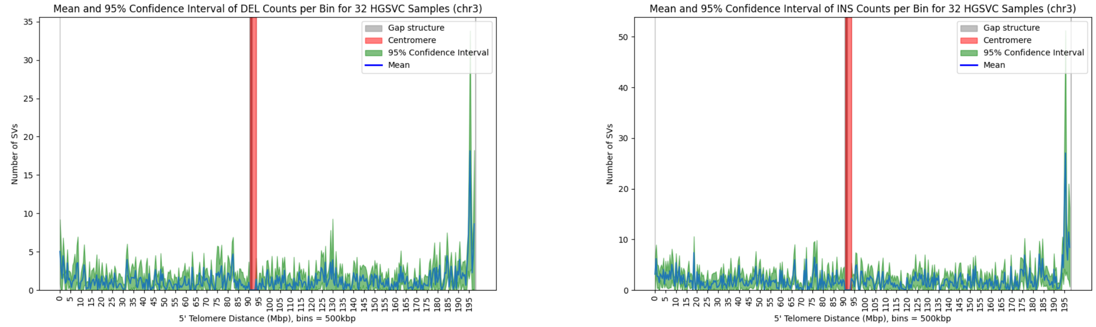

(c) Deletions and insertions for chromosome 3

Figure S3: Mean SV counts and 95% confidence intervals across chromosomes (32 *HGSVC* Samples) for chromosomes 1 to 3.

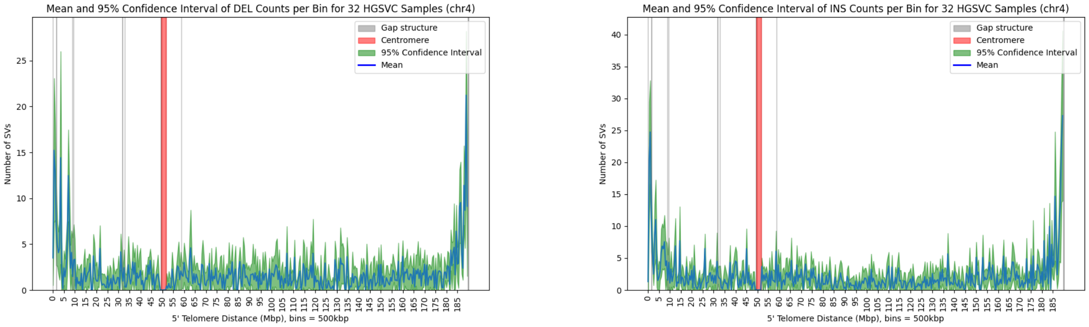

(d) Deletions and insertions for chromosome 4

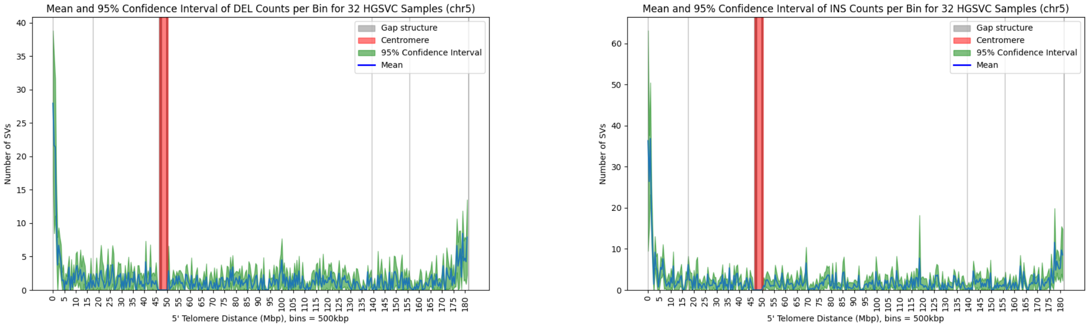

(e) Deletions and insertions for chromosome 5

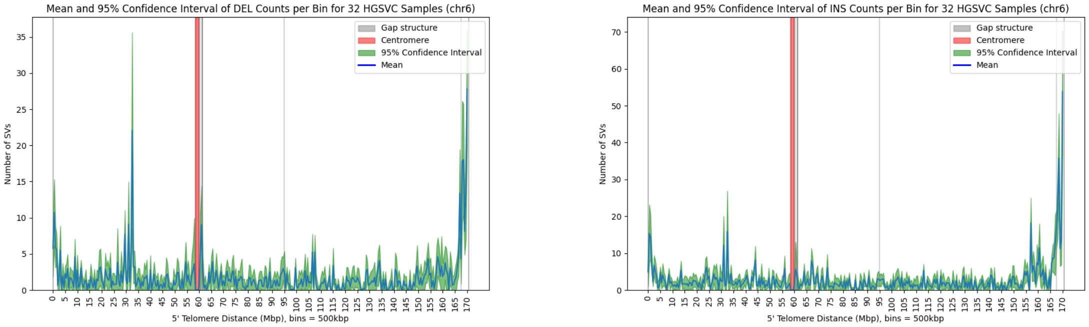

(f) Deletions and insertions for chromosome 6

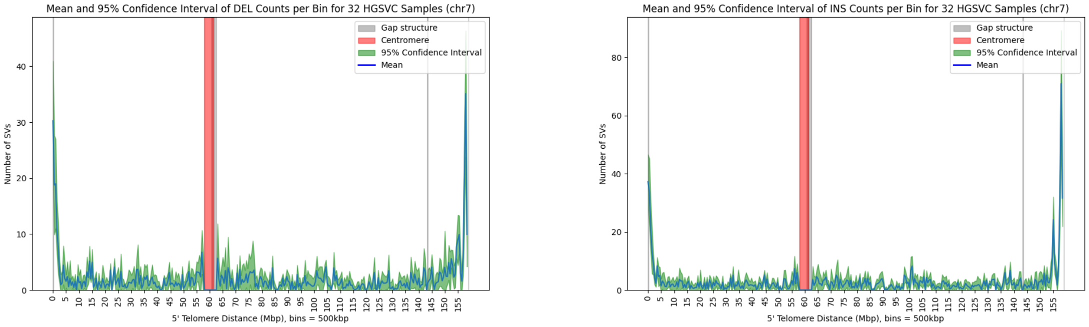

(g) Deletions and insertions for chromosome 7

Figure S3: Mean SV counts and 95% confidence intervals across chromosomes (32 *HGSVC* Samples) for chromosomes 4 to 7.

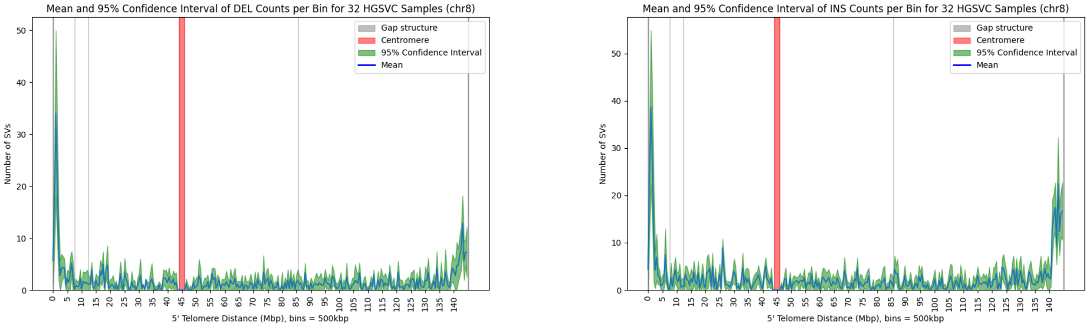

(h) Deletions and insertions for chromosome 8

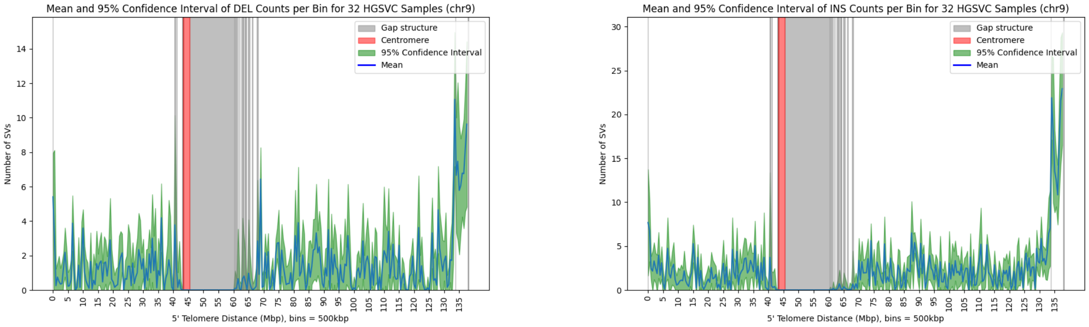

(i) Deletions and insertions for chromosome 9

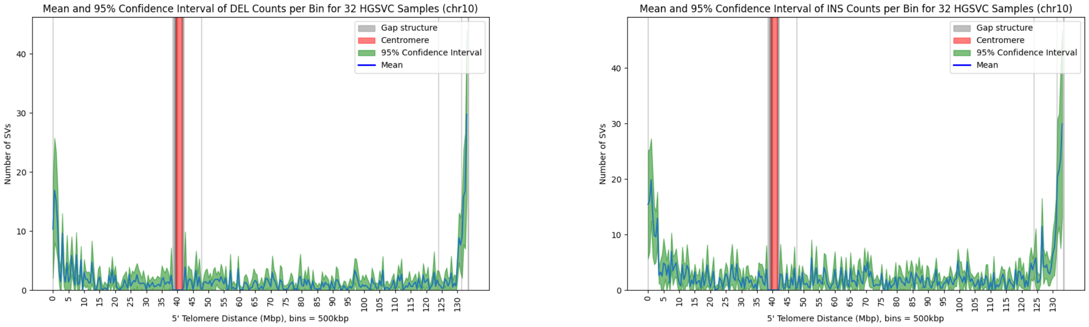

(j) Deletions and insertions for chromosome 10

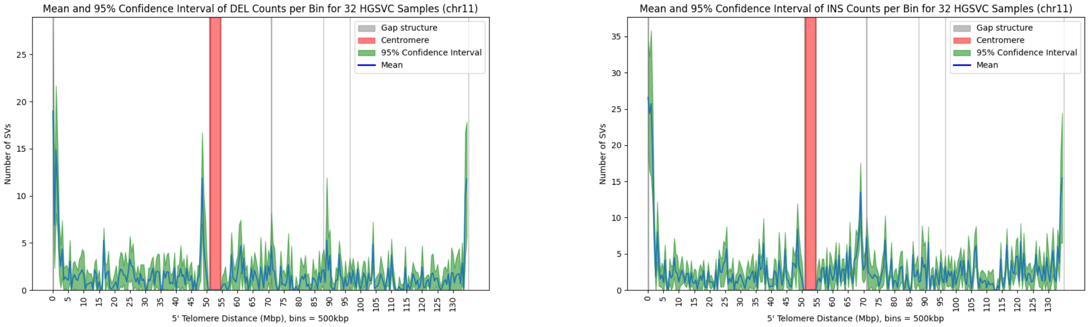

(k) Deletions and insertions for chromosome 11

Figure S3: Mean SV counts and 95% confidence intervals across chromosomes (32 *HGSVC* Samples) for chromosomes 8 to 11.

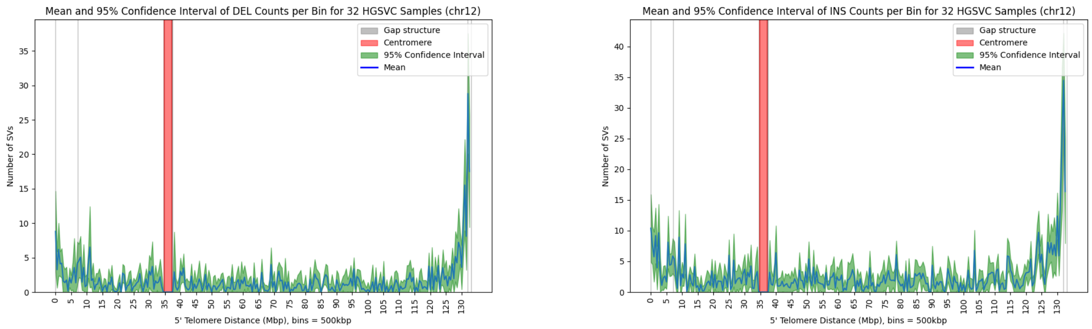

(l) Deletions and insertions for chromosome 12

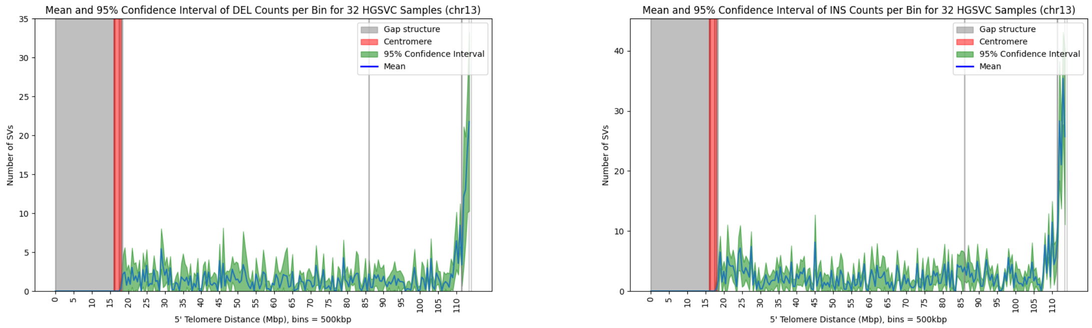

(m) Deletions and insertions for chromosome 13

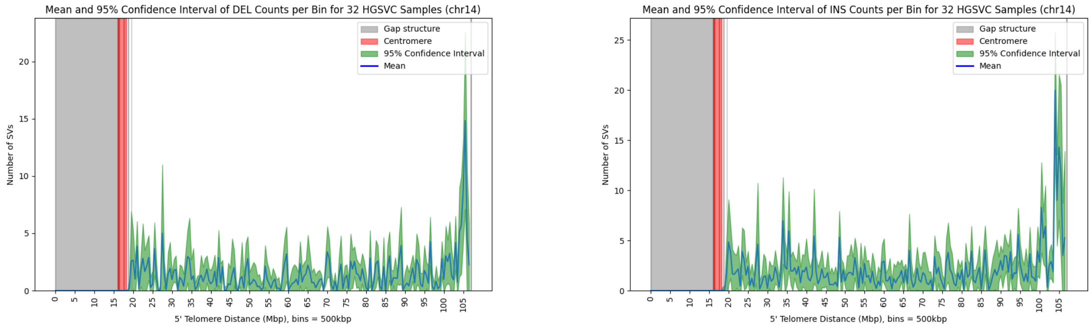

(n) Deletions and insertions for chromosome 14

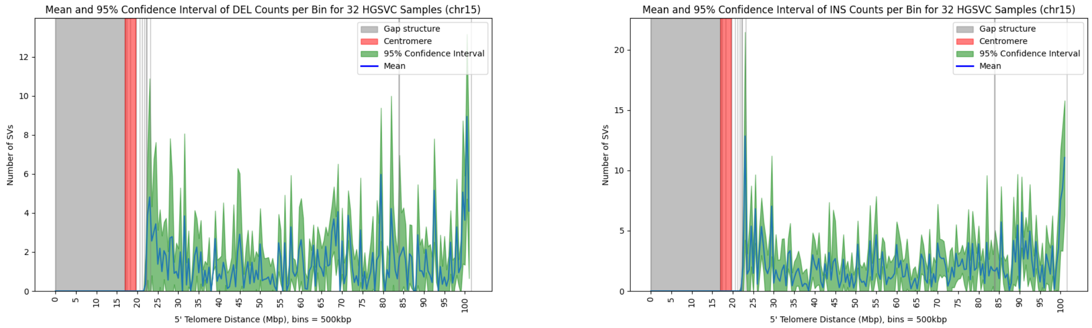

(o) Deletions and insertions for chromosome 15

Figure S3: Mean SV counts and 95% confidence intervals across chromosomes (32 *HGSVC* Samples) for chromosomes 12 to 15.

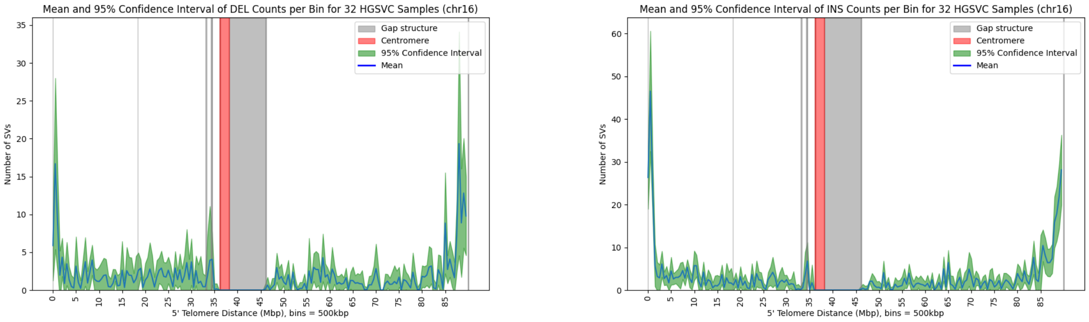

(p) Deletions and insertions for chromosome 16

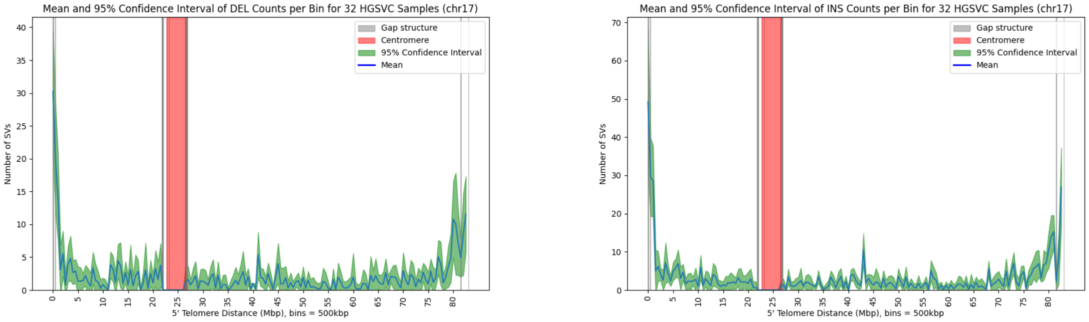

(q) Deletions and insertions for chromosome 17

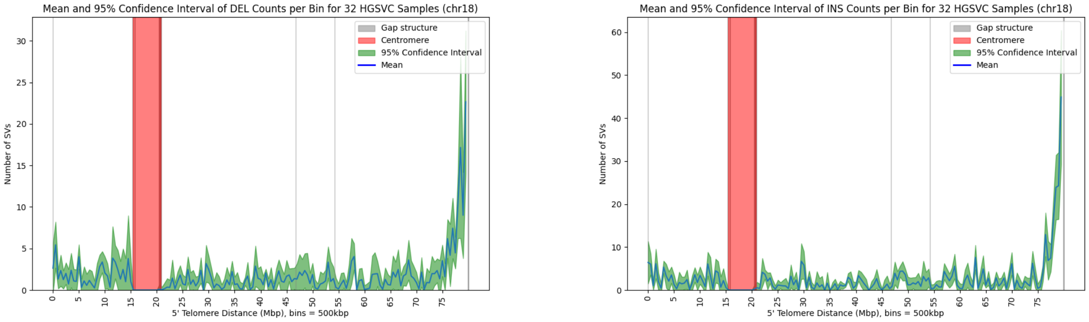

(r) Deletions and insertions for chromosome 18

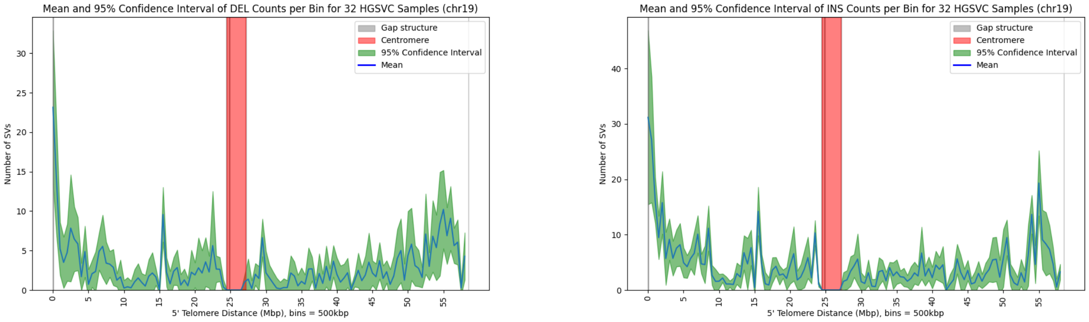

(s) Deletions and insertions for chromosome 19

Figure S3: Mean SV counts and 95% confidence intervals across chromosomes (32 *HGSVC* Samples) for chromosomes 16 to 19.

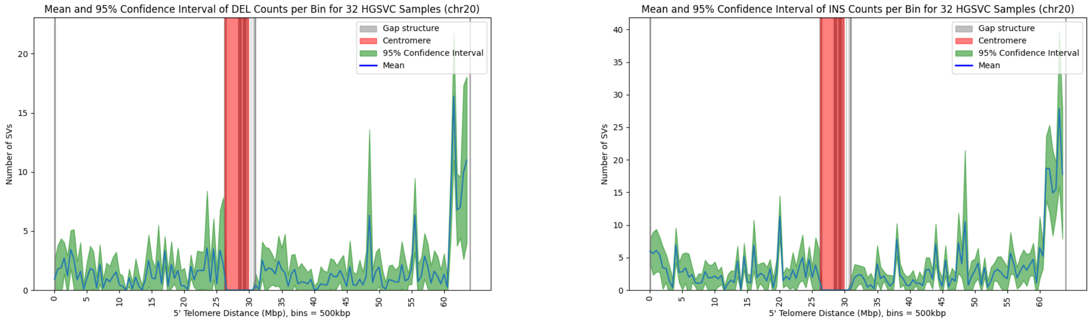

(t) Deletions and insertions for chromosome 20

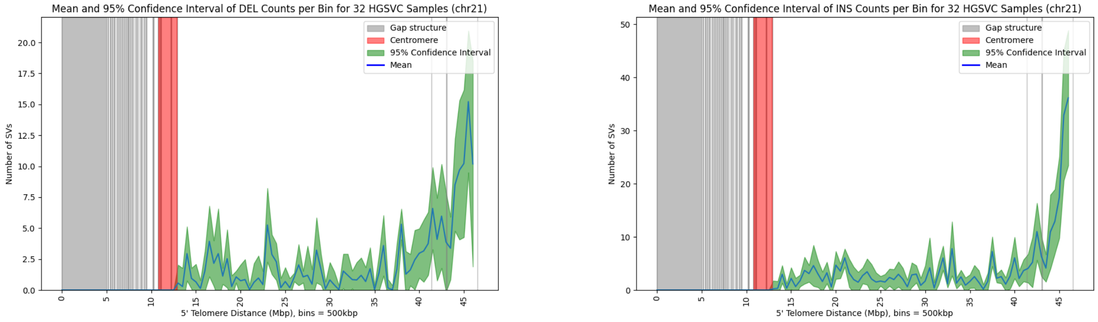

(u) Deletions and insertions for chromosome 21

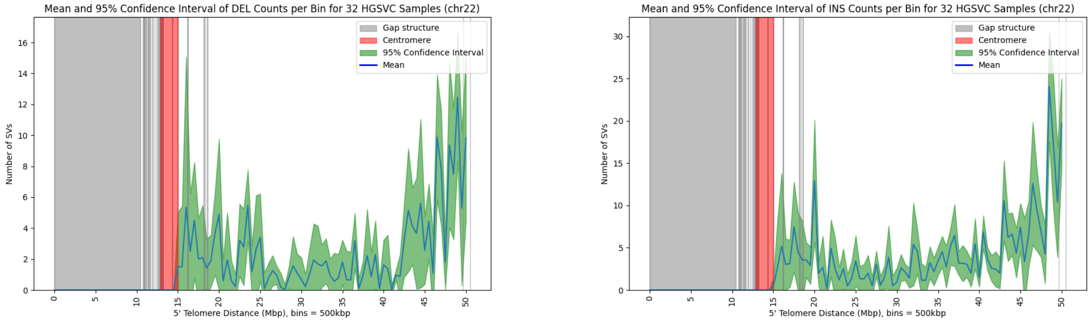

(v) Deletions and insertions for chromosome 22

Figure S3: Mean SV counts and 95% confidence intervals across chromosomes (32 *HGSVC* Samples) for chromosomes 20 to 22.

1.3 Higher rates of SVs in TR regions

1.3.1 15 *Cell* Samples and HG002

Figure S4 shows the distribution of deletions and insertions in TR regions and non-tandem repeat (NonTR) regions for the 15 *Cell* samples and HG002. Deep blue indicates deletions in TR regions, light blue shows deletions in NonTR regions, deep red represents insertions in TR regions, and light pink denotes insertions in NonTR regions.

Table S1 and Table S2 present the counts of deletions and insertions in TR regions across the 15 *Cell* samples. In both tables, the values in parentheses indicate the proportion of deletions in TR regions or insertions in TR regions relative to the total deletions or insertions for each chromosome. Additionally, each table includes the mean and standard deviation of TR counts, as well as the mean and standard deviation of TR region proportions and their ratios to chromosome lengths. Notably, TR regions are relatively small, accounting for approximately 2.78% (in chromosome 19) to 10.76% (in chromosome 11) of each chromosome's length. Despite their limited size, these regions harbor a significant proportion of both deletions and insertions.

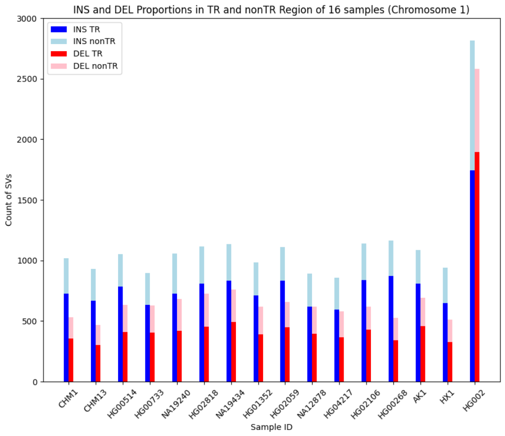

(a) Chromosome 1

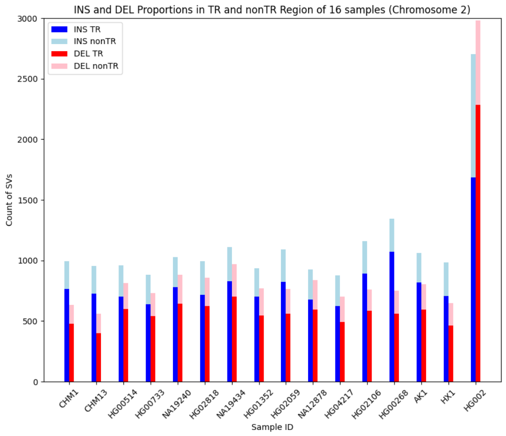

(b) Chromosome 2

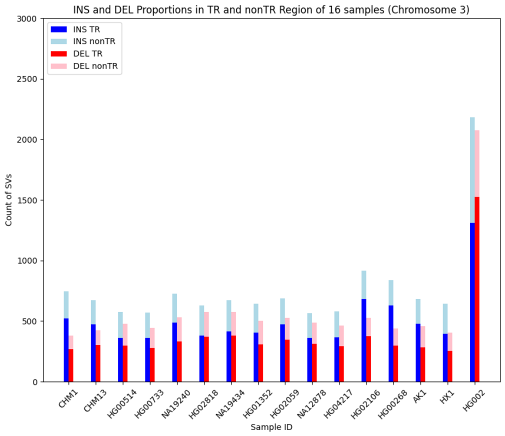

(c) Chromosome 3

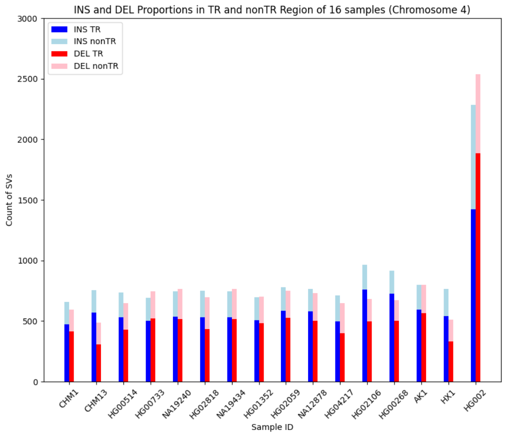

(d) Chromosome 4

Figure S4: Distribution of insertions and deletions in tandem repeat and non-tandem repeat regions in 15 *Cell* samples and HG002 for chromosome 1 to 4

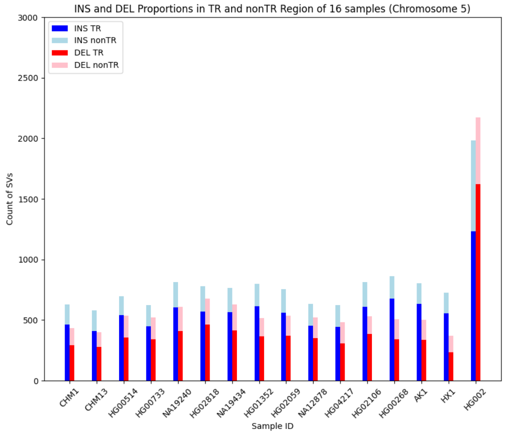

(e) Chromosome 5

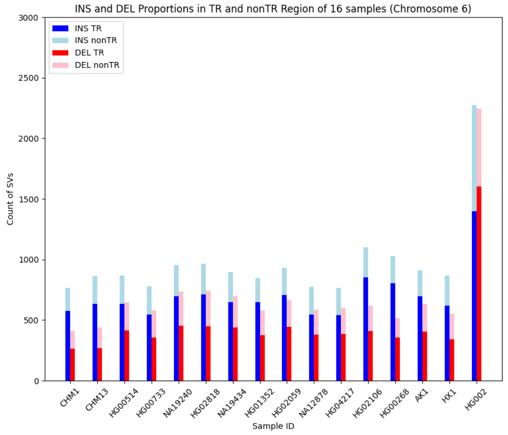

(f) Chromosome 6

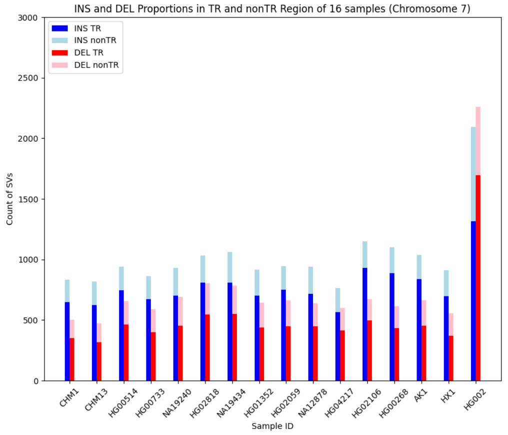

(g) Chromosome 7

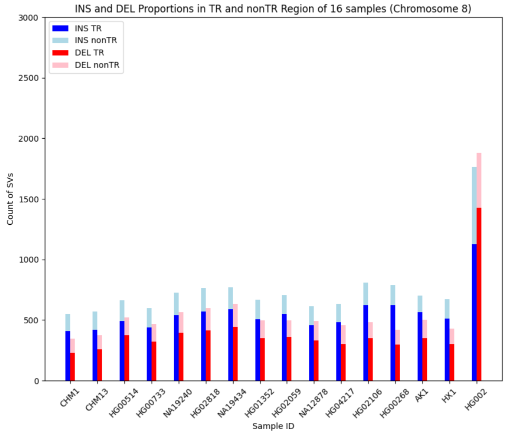

(h) Chromosome 8

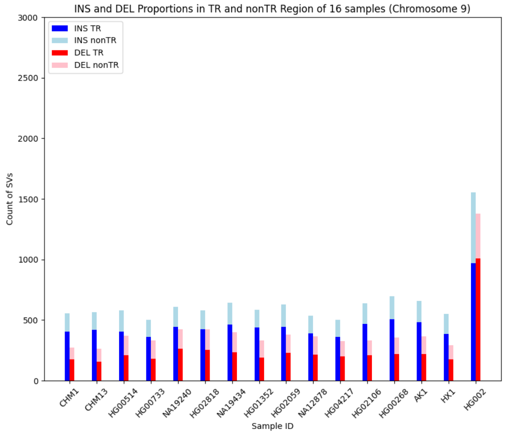

(i) Chromosome 9

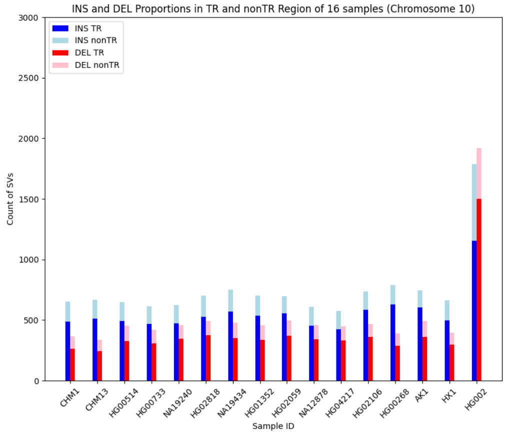

(j) Chromosome 10

Figure S4: Distribution of insertions and deletions in tandem repeat and non-tandem repeat regions in 15 *Cell* samples and HG002 for chromosome 5 to 10

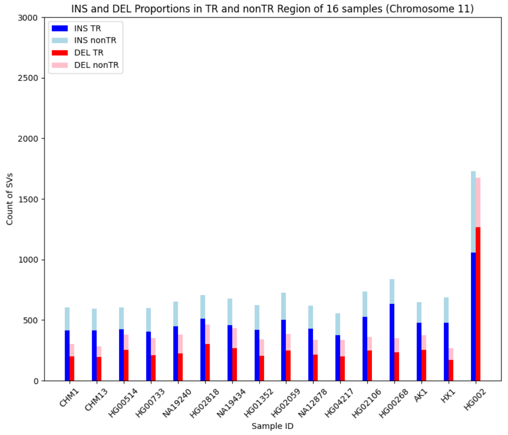

(k) Chromosome 11

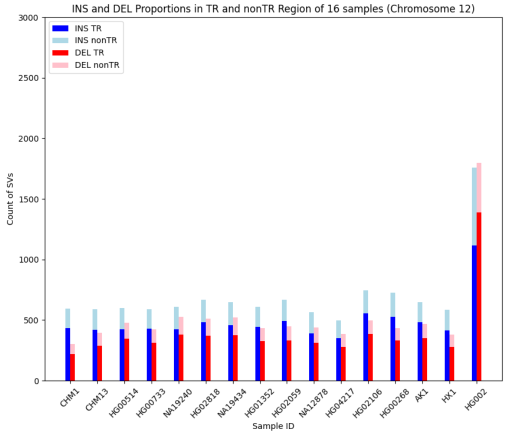

(l) Chromosome 12

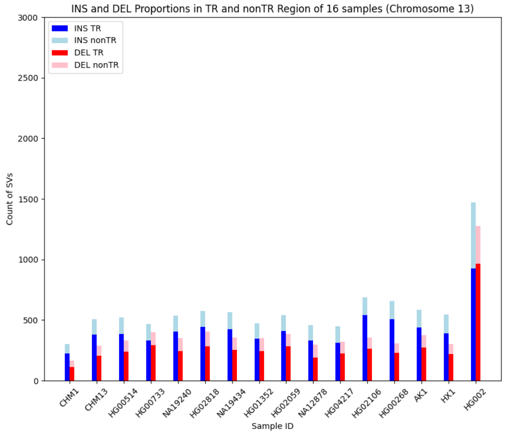

(m) Chromosome 13

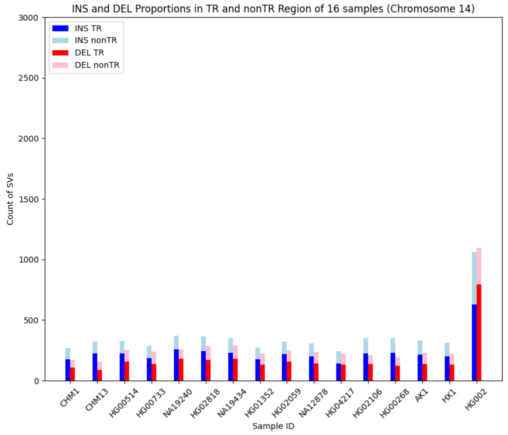

(n) Chromosome 14

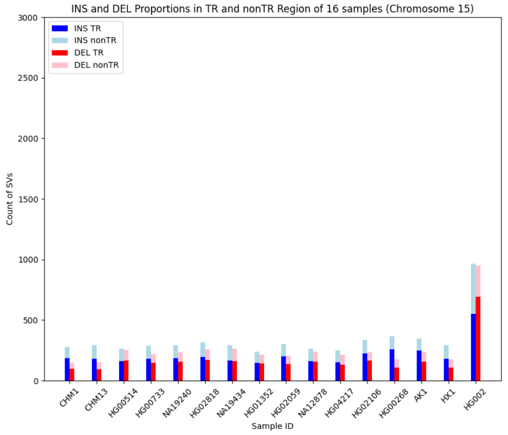

(o) Chromosome 15

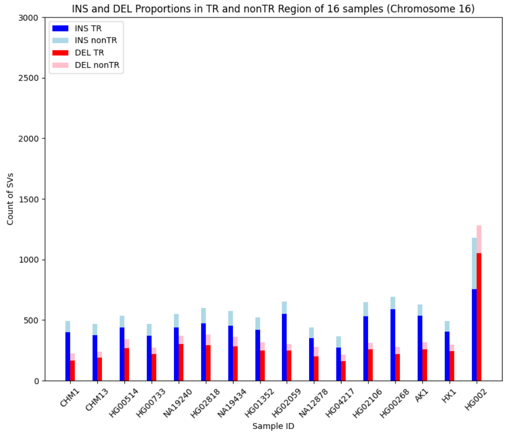

(p) Chromosome 16

Figure S4: Distribution of insertions and deletions in tandem repeat and non-tandem repeat regions in 15 *Cell* samples and HG002 for chromosome 11 to 16

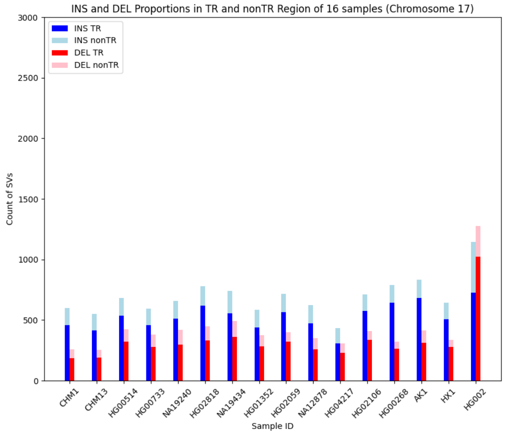

(q) Chromosome 17

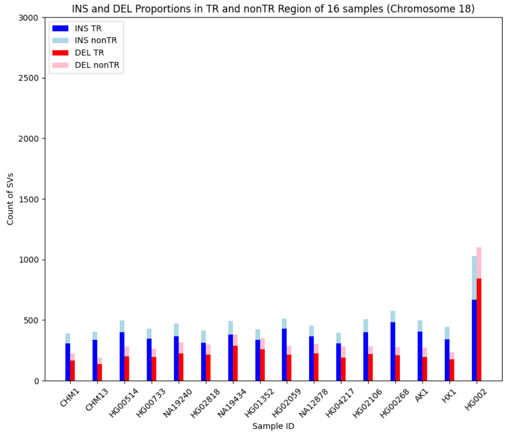

(r) Chromosome 18

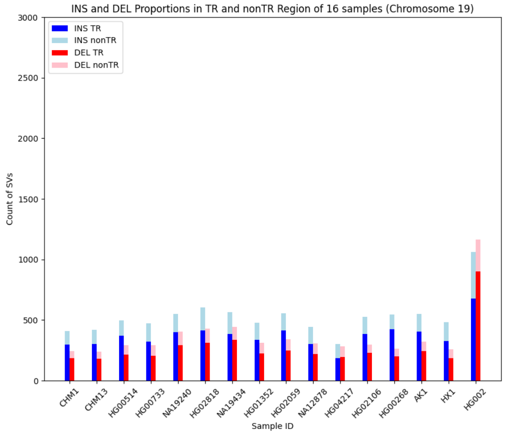

(s) Chromosome 19

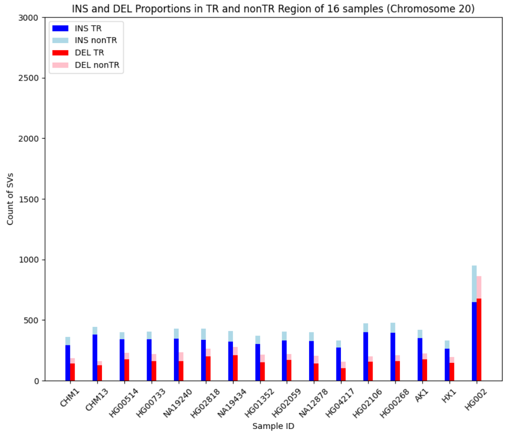

(t) Chromosome 20

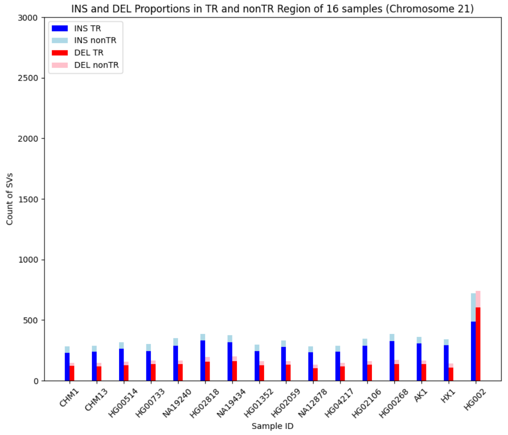

(u) Chromosome 21

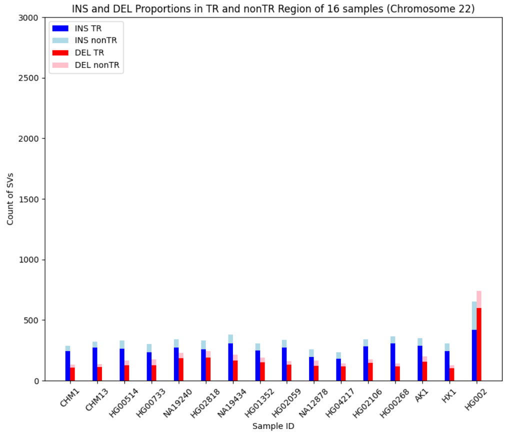

(v) Chromosome 22

Figure S4: Distribution of insertions and deletions in tandem repeat and non-tandem repeat regions in 15 *Cell* samples and HG002 for chromosome 17 to 22

Table S1: Counts and proportions of deletions in tandem repeat regions across 15 *Cell* samples

TR DEL(%)	CHM1	CHM13	HG00514	HG00733	NA19240	HG02818	NA19434	HG01352	HG02059	NA12878	HG04217	HG02106	HG00268	AK1	HX1	total.DEL.mean.sd.	TR ratio mean(sd)	TR.region.ratio
chr1	355(67.11%)	300(64.38%)	410(64.87%)	406(64.86%)	420(61.58%)	452(62.17%)	490(64.47%)	389(62.94%)	446(67.68%)	394(63.55%)	364(62.65%)	428(69.26%)	339(64.57%)	458(66.18%)	325(63.6%)	616.40(80.01)	64.66%(2.13%)	3.47%
chr2	479(75.43%)	400(71.17%)	600(73.89%)	541(74.11%)	641(72.59%)	623(72.53%)	700(72.31%)	545(70.96%)	560(73.39%)	595(71.09%)	494(70.27%)	583(76.61%)	562(75.03%)	592(73.45%)	463(71.56%)	765.53(100.14)	72.96%(1.83%)	4.34%
chr3	268(70.9%)	302(71.06%)	298(62.61%)	276(62.02%)	329(62.08%)	370(64.46%)	378(65.62%)	305(60.64%)	348(65.91%)	310(63.79%)	292(63.34%)	373(71.05%)	298(68.04%)	284(62.28%)	254(62.87%)	480.33(56.92)	65.11%(3.56%)	5.18%
chr4	412(69.36%)	309(63.45%)	431(66.31%)	521(70.03%)	516(67.28%)	432(62.25%)	515(67.32%)	482(68.66%)	526(70.04%)	502(68.77%)	400(61.63%)	497(73.09%)	501(74.55%)	563(70.64%)	333(65.04%)	679.60(87.78)	67.89%(3.72%)	4.72%
chr5	290(66.97%)	276(69.35%)	356(66.17%)	339(64.94%)	407(66.72%)	465(68.89%)	413(65.87%)	365(70.6%)	368(68.66%)	349(67.12%)	307(63.83%)	385(72.37%)	343(67.65%)	335(66.47%)	236(63.61%)	518.07(77.83)	67.28%(2.40%)	3.42%
chr6	265(64.79%)	268(60.91%)	412(63.78%)	356(61.38%)	455(61.99%)	447(60.57%)	439(63.07%)	377(65.0%)	443(66.32%)	381(65.46%)	385(64.38%)	409(66.18%)	354(68.47%)	403(63.67%)	342(62.18%)	599.27(92.00)	63.88%(2.24%)	3.80%
chr7	349(69.66%)	317(67.3%)	461(70.06%)	399(67.63%)	454(65.7%)	545(67.96%)	548(69.81%)	437(67.75%)	450(68.18%)	446(69.91%)	414(69.0%)	497(73.96%)	434(70.68%)	453(68.53%)	371(66.97%)	636.13(86.38)	68.87%(1.96%)	4.34%
chr8	231(67.15%)	258(69.17%)	375(72.25%)	319(68.16%)	393(69.56%)	413(68.95%)	443(69.98%)	353(70.74%)	358(72.03%)	329(66.87%)	303(66.01%)	353(73.24%)	296(70.48%)	351(70.2%)	302(70.56%)	485.20(74.81)	69.69%(2.05%)	5.86%
chr9	176(64.23%)	158(60.54%)	211(57.18%)	180(54.71%)	265(62.5%)	253(59.67%)	235(58.9%)	192(57.66%)	231(60.95%)	216(59.34%)	200(60.98%)	211(63.94%)	217(60.96%)	217(59.78%)	176(60.69%)	348.20(47.48)	60.13%(2.48%)	8.19%
chr10	261(71.7%)	243(72.54%)	327(72.19%)	308(73.86%)	345(75.66%)	373(76.12%)	350(73.53%)	334(73.25%)	368(74.34%)	341(74.78%)	331(74.22%)	360(76.76%)	285(73.26%)	362(73.88%)	298(75.25%)	439.20(46.90)	74.09%(1.44%)	8.61%
chr11	200(66.23%)	193(68.2%)	255(67.11%)	209(59.89%)	225(59.52%)	302(65.37%)	270(62.5%)	204(59.82%)	247(64.32%)	213(63.39%)	201(59.64%)	249(68.78%)	236(67.62%)	255(67.82%)	169(63.53%)	355.80(49.48)	64.25%(3.38%)	10.76%
chr12	217(71.85%)	289(73.35%)	348(73.26%)	310(73.11%)	378(72.14%)	372(72.94%)	376(71.89%)	325(75.23%)	332(73.94%)	313(71.62%)	276(71.69%)	386(77.98%)	331(76.44%)	351(75.16%)	279(73.42%)	442.00(58.88)	73.60%(1.87%)	2.91%
chr13	110(65.48%)	204(71.33%)	238(71.9%)	292(72.82%)	244(69.32%)	283(69.88%)	252(70.99%)	243(69.63%)	284(73.96%)	192(64.86%)	222(69.59%)	261(73.52%)	227(73.46%)	274(72.68%)	221(73.42%)	332.53(56.88)	70.86%(2.80%)	7.42%
chr14	108(63.16%)	89(57.42%)	156(61.9%)	136(56.9%)	178(68.73%)	171(60.42%)	178(60.96%)	133(59.11%)	155(62.0%)	139(59.4%)	130(59.36%)	136(64.76%)	122(61.93%)	134(58.01%)	132(60.83%)	228.93(35.93)	60.99%(3.03%)	7.87%
chr15	99(67.35%)	94(62.67%)	168(65.88%)	148(66.97%)	157(67.09%)	169(65.25%)	162(61.6%)	143(66.82%)	138(67.65%)	154(64.71%)	132(61.4%)	167(71.06%)	107(60.8%)	157(66.81%)	106(60.92%)	214.67(36.27)	65.13%(3.04%)	8.20%
chr16	167(74.55%)	192(80.33%)	267(78.3%)	217(80.07%)	300(80.86%)	292(76.64%)	283(78.39%)	246(77.36%)	247(81.25%)	201(71.79%)	159(73.95%)	256(81.53%)	217(78.34%)	260(81.76%)	242(81.21%)	300.80(49.23)	78.42%(3.07%)	3.31%
chr17	186(71.54%)	191(75.2%)	321(76.07%)	276(73.02%)	295(70.07%)	333(74.0%)	359(73.12%)	282(74.8%)	321(80.25%)	259(73.79%)	231(74.76%)	336(81.75%)	263(81.42%)	313(75.42%)	276(81.66%)	373.33(65.20)	75.79%(3.75%)	2.90%
chr18	168(74.34%)	135(70.68%)	198(70.46%)	196(75.1%)	223(70.35%)	214(72.05%)	287(75.53%)	256(73.14%)	212(74.13%)	224(74.17%)	190(67.14%)	218(77.3%)	208(75.36%)	193(70.44%)	177(75.0%)	282.80(44.84)	73.01%(2.73%)	4.07%
chr19	184(75.41%)	180(75.0%)	214(72.79%)	204(69.62%)	293(72.52%)	313(73.13%)	335(75.62%)	224(71.79%)	246(71.72%)	220(71.66%)	196(69.26%)	228(77.03%)	199(76.25%)	243(75.23%)	183(70.38%)	315.40(61.60)	73.16%(2.47%)	2.78%
chr20	143(77.3%)	128(78.53%)	173(75.22%)	161(73.52%)	162(69.53%)	201(76.72%)	210(76.09%)	152(70.37%)	169(77.52%)	139(68.47%)	104(67.1%)	155(77.5%)	159(76.08%)	176(78.57%)	145(74.74%)	212.47(31.13)	74.48%(3.81%)	5.09%
chr21	122(84.14%)	118(81.94%)	127(80.38%)	138(82.14%)	135(80.36%)	157(81.35%)	159(79.9%)	126(78.26%)	131(80.37%)	104(78.2%)	119(82.64%)	133(83.12%)	138(81.66%)	134(80.72%)	108(76.6%)	160.80(17.68)	80.78%(2.01%)	3.72%
chr22	106(79.7%)	111(81.62%)	128(77.11%)	126(72.0%)	184(80.7%)	191(78.93%)	168(78.14%)	149(79.26%)	130(81.25%)	124(75.15%)	117(82.39%)	144(82.76%)	115(82.14%)	158(79.0%)	101(78.29%)	172.87(34.40)	79.23%(2.91%)	3.86%

Table S2: Counts and proportions of insertions in tandem repeat regions across 15 *Cell* samples

TR INS(%)	CHM1	CHM13	HG00514	HG00733	NA19240	HG02818	NA19434	HG01352	HG02059	NA12878	HG04217	HG02106	HG00268	AK1	HX1	total_INS_mean(sd)	TR ratio mean(sd)	TR_ratio(%)
chr1	724(71.12%)	666(71.46%)	784(74.38%)	634(70.92%)	726(68.68%)	807(72.31%)	832(73.37%)	709(71.91%)	833(74.91%)	618(69.52%)	592(69.08%)	836(73.4%)	870(74.87%)	808(74.33%)	650(69.22%)	1025.07(98.89)	71.97%(2.19%)	3.47%
chr2	767(77.09%)	725(75.92%)	701(73.25%)	638(72.34%)	778(75.53%)	717(72.21%)	828(74.59%)	699(74.68%)	824(75.53%)	677(73.27%)	624(71.31%)	893(76.92%)	1073(79.9%)	819(77.19%)	707(71.7%)	1019.93(117.24)	74.76%(2.44%)	4.34%
chr3	521(70.03%)	471(70.09%)	358(62.15%)	362(63.29%)	485(66.62%)	378(60.1%)	414(61.79%)	402(62.52%)	470(68.41%)	360(63.49%)	365(63.04%)	683(74.48%)	629(75.24%)	478(70.09%)	393(61.31%)	676.20(95.86)	66.18%(4.90%)	5.18%
chr4	473(72.1%)	572(75.96%)	532(72.48%)	501(72.5%)	537(72.27%)	531(70.71%)	532(71.41%)	508(73.2%)	585(75.19%)	578(75.36%)	497(70.1%)	759(78.9%)	724(78.95%)	594(74.53%)	539(70.27%)	764.27(77.81)	73.60%(2.83%)	4.72%
chr5	463(73.61%)	407(70.42%)	541(77.51%)	450(72.12%)	606(74.36%)	570(72.98%)	565(73.76%)	612(76.69%)	559(73.94%)	453(71.68%)	444(71.15%)	608(74.69%)	677(78.36%)	633(78.73%)	554(76.52%)	727.13(86.88)	74.43%(2.62%)	3.42%
chr6	575(75.16%)	633(73.52%)	631(72.7%)	544(69.65%)	696(73.03%)	713(74.12%)	648(72.4%)	650(76.65%)	706(75.91%)	544(70.47%)	540(70.77%)	851(77.36%)	804(78.29%)	697(76.34%)	619(71.48%)	886.93(94.96)	73.86%(2.68%)	3.80%
chr7	647(77.67%)	623(76.35%)	744(79.15%)	674(78.01%)	703(75.75%)	808(78.22%)	808(76.01%)	702(76.47%)	752(79.75%)	715(75.9%)	563(73.4%)	928(80.7%)	884(80.29%)	839(81.06%)	695(76.46%)	949.47(105.08)	77.68%(2.19%)	4.34%
chr8	408(74.18%)	417(73.16%)	493(74.7%)	440(73.58%)	541(74.52%)	572(74.77%)	590(76.52%)	508(76.28%)	551(78.27%)	460(75.04%)	480(76.07%)	622(76.79%)	622(78.83%)	563(80.09%)	509(75.52%)	682.00(77.76)	75.89%(1.97%)	5.86%
chr9	403(72.88%)	417(74.07%)	402(69.31%)	358(71.17%)	443(72.62%)	425(73.28%)	463(71.89%)	438(75.0%)	442(70.49%)	390(72.76%)	361(72.2%)	466(73.04%)	508(72.88%)	483(73.63%)	383(69.76%)	588.00(54.98)	72.33%(1.58%)	8.19%
chr10	486(74.54%)	513(77.03%)	493(75.85%)	469(76.14%)	472(75.88%)	524(74.96%)	568(75.94%)	538(76.53%)	554(79.37%)	452(74.34%)	424(73.87%)	583(79.54%)	626(79.44%)	603(81.05%)	495(75.0%)	677.40(58.00)	76.63%(2.21%)	8.61%
chr11	415(68.71%)	413(69.41%)	425(70.48%)	403(67.28%)	450(68.81%)	510(72.03%)	459(67.8%)	421(67.79%)	503(69.38%)	429(69.53%)	375(67.69%)	526(71.37%)	635(75.6%)	476(73.23%)	479(69.62%)	658.13(70.69)	69.92%(2.31%)	10.76%
chr12	435(72.99%)	418(70.73%)	426(71.24%)	430(72.88%)	422(69.07%)	482(72.16%)	456(70.37%)	443(72.74%)	494(74.17%)	392(69.26%)	349(69.94%)	555(74.3%)	525(72.31%)	480(74.3%)	413(70.84%)	622.93(60.58)	71.82%(1.77%)	2.91%
chr13	224(74.17%)	381(75.45%)	387(74.28%)	329(70.15%)	403(75.33%)	442(77.14%)	425(75.09%)	346(73.0%)	408(75.42%)	330(71.9%)	314(69.93%)	539(78.46%)	507(77.17%)	436(74.79%)	391(71.74%)	524.40(88.61)	74.27%(2.51%)	7.42%
chr14	173(64.55%)	226(70.85%)	223(68.83%)	186(65.03%)	257(69.09%)	242(66.48%)	230(65.9%)	175(64.34%)	217(67.18%)	198(64.92%)	143(58.37%)	224(63.82%)	230(65.71%)	213(64.74%)	199(63.78%)	317.93(36.02)	65.57%(2.88%)	7.87%
chr15	183(66.3%)	179(61.51%)	162(62.07%)	180(62.28%)	185(62.93%)	195(61.71%)	168(57.73%)	144(60.0%)	199(65.68%)	162(61.36%)	151(60.4%)	226(67.66%)	258(70.3%)	250(72.25%)	180(61.86%)	294.20(34.07)	63.60%(4.02%)	8.20%
chr16	401(81.34%)	373(79.7%)	439(81.9%)	371(79.61%)	436(79.42%)	474(79.0%)	453(78.65%)	420(80.46%)	550(83.97%)	351(79.95%)	274(74.66%)	530(81.54%)	589(84.99%)	538(85.53%)	403(81.74%)	542.40(88.35)	80.83%(2.72%)	3.31%
chr17	458(76.72%)	414(75.27%)	538(79.12%)	457(77.07%)	510(77.51%)	617(79.2%)	553(74.73%)	440(75.6%)	566(78.94%)	470(75.68%)	308(70.97%)	575(80.65%)	645(81.85%)	683(82.09%)	506(78.82%)	661.73(100.83)	77.61%(2.95%)	2.90%
chr18	306(78.06%)	334(82.47%)	400(80.32%)	344(80.0%)	363(77.07%)	310(75.24%)	382(77.64%)	336(79.06%)	427(83.73%)	367(80.66%)	306(77.66%)	399(78.85%)	480(83.48%)	406(82.02%)	343(77.6%)	460.13(50.62)	79.59%(2.51%)	4.07%
chr19	296(72.55%)	302(71.9%)	368(73.9%)	321(68.01%)	400(72.46%)	415(68.94%)	387(68.25%)	337(70.5%)	414(74.46%)	302(67.87%)	186(61.18%)	384(73.14%)	422(77.57%)	406(74.09%)	326(67.78%)	493.33(74.33)	70.84%(3.97%)	2.78%
chr20	291(81.28%)	381(85.62%)	339(84.96%)	342(84.44%)	346(80.47%)	337(78.55%)	320(78.05%)	301(81.35%)	333(82.22%)	328(82.0%)	275(83.33%)	400(85.11%)	394(82.6%)	350(83.14%)	262(78.92%)	405.40(42.38)	82.14%(2.40%)	5.09%
chr21	231(81.34%)	237(82.58%)	265(83.6%)	242(80.4%)	285(81.2%)	329(85.01%)	316(84.72%)	244(82.71%)	277(83.69%)	233(82.33%)	240(83.62%)	286(82.42%)	328(85.64%)	309(86.07%)	291(85.34%)	328.40(36.35)	83.38%(1.73%)	3.72%
chr22	243(84.08%)	272(84.74%)	261(79.09%)	233(77.67%)	271(79.47%)	259(78.01%)	307(81.0%)	248(81.31%)	273(80.77%)	196(75.97%)	182(77.12%)	281(82.4%)	305(83.11%)	289(82.81%)	245(79.55%)	319.60(37.07)	80.47%(2.64%)	3.86%

1.3.2 32 *HGSVC* Samples

Figure S5 shows the distribution of deletions and insertions in TR and NonTR regions for 32 *HGSVC* samples, revealing patterns consistent with those observed in the 15 *Cell* samples. Tables S3 and S4 present the TR region variant counts for these 32 samples, demonstrating similar trends to Tables S1 and S2 from the *Cell* cohort.

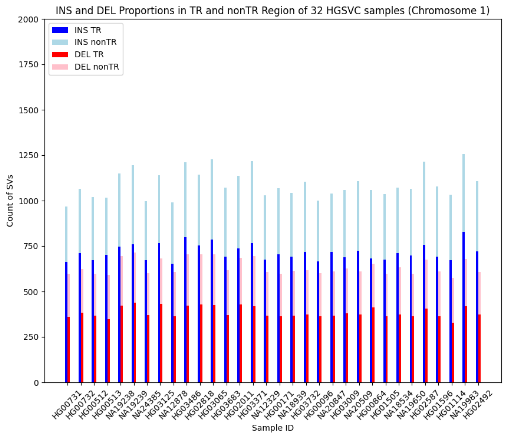

(a) Chromosome 1

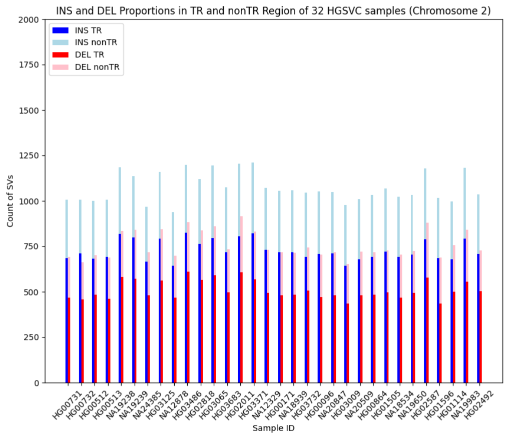

(b) Chromosome 2

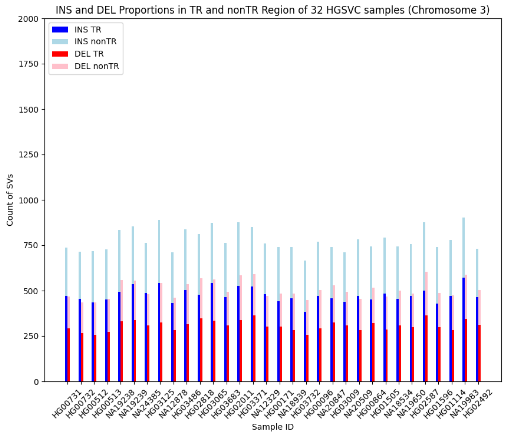

(c) Chromosome 3

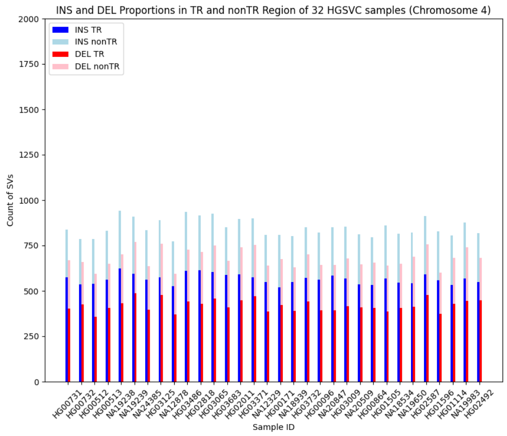

(d) Chromosome 4

Figure S5: Distribution of insertions and deletions in tandem repeat and non-tandem repeat regions in 32 *HGSVC* samples for chromosome 1 to 4

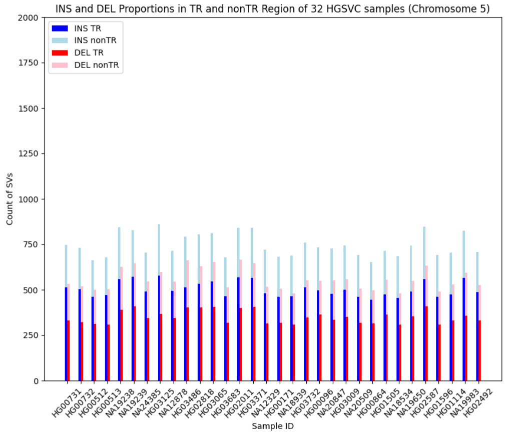

(e) Chromosome 5

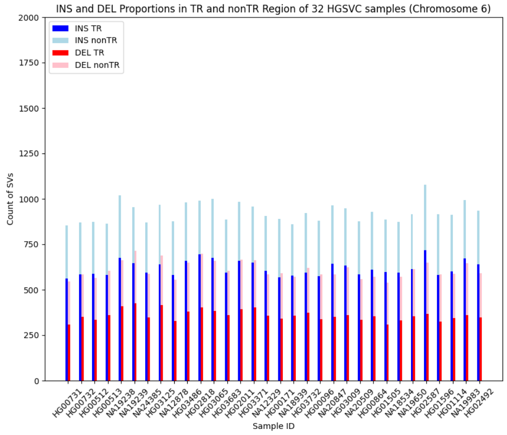

(f) Chromosome 6

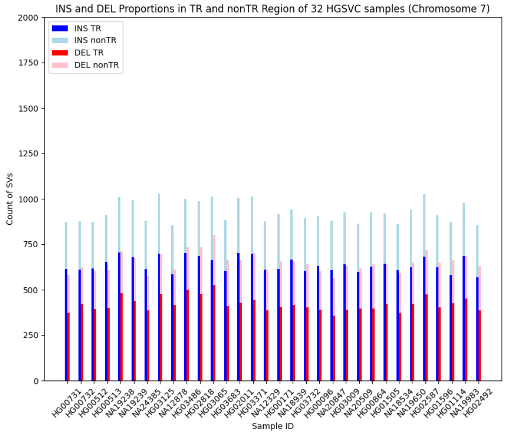

(g) Chromosome 7

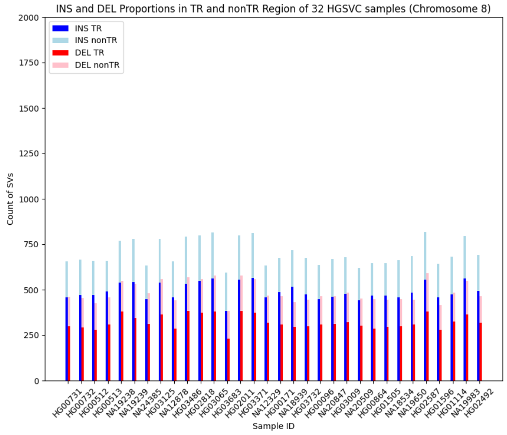

(h) Chromosome 8

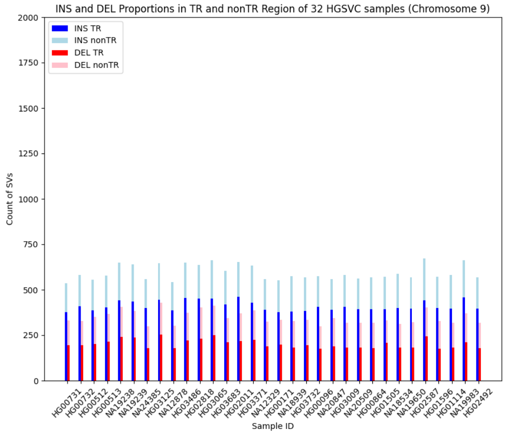

(i) Chromosome 9

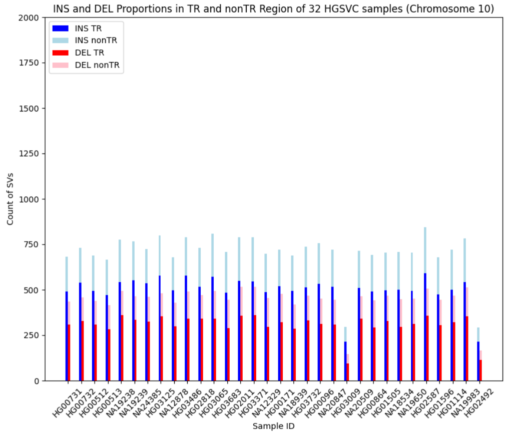

(j) Chromosome 10

Figure S5: Distribution of insertions and deletions in tandem repeat and non-tandem repeat regions in 32 *HGSVC* samples for chromosome 5 to 10

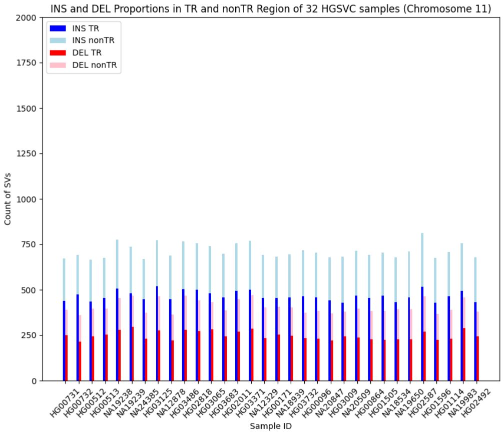

(k) Chromosome 11

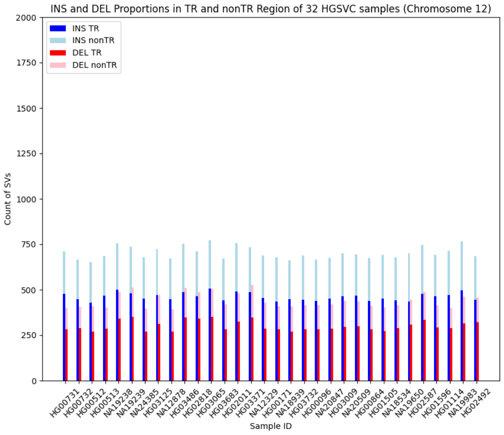

(l) Chromosome 12

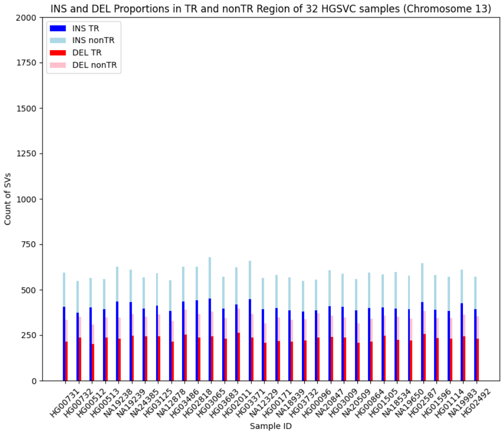

(m) Chromosome 13

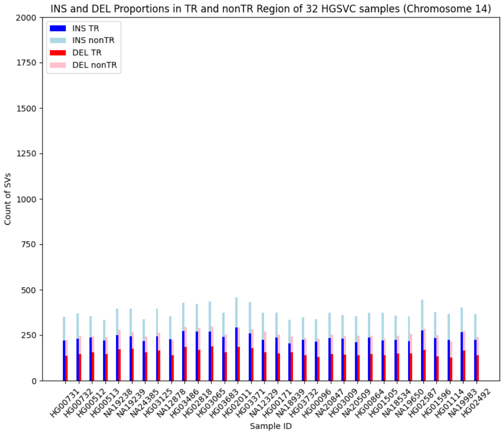

(n) Chromosome 14

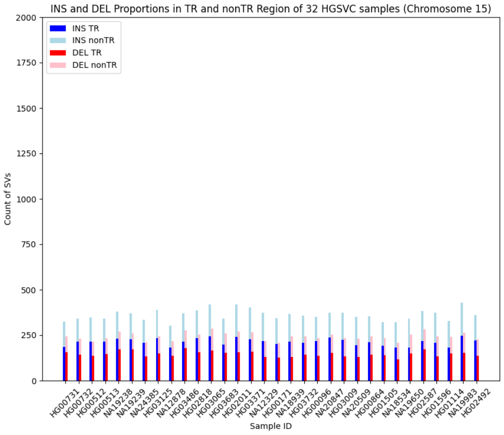

(o) Chromosome 15

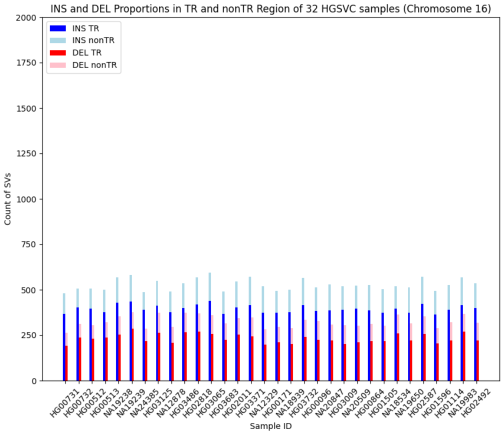

(p) Chromosome 16

Figure S5: Distribution of insertions and deletions in tandem repeat and non-tandem repeat regions in 32 *HGSVC* samples for chromosome 11 to 16

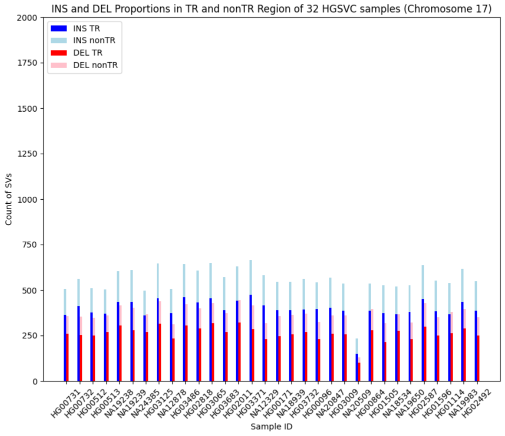

(q) Chromosome 17

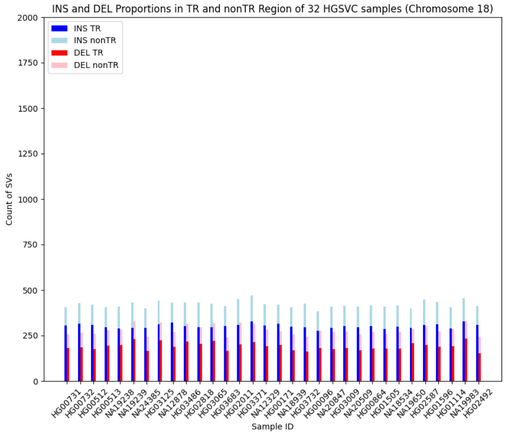

(r) Chromosome 18

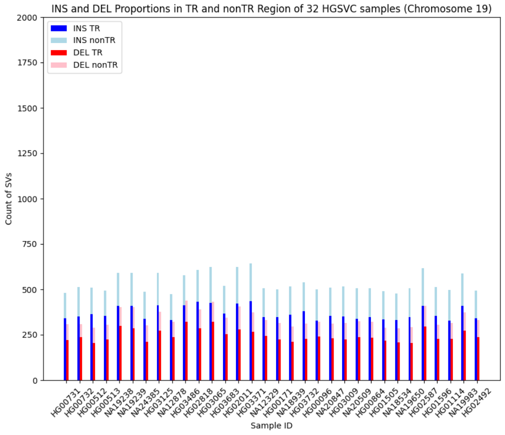

(s) Chromosome 19

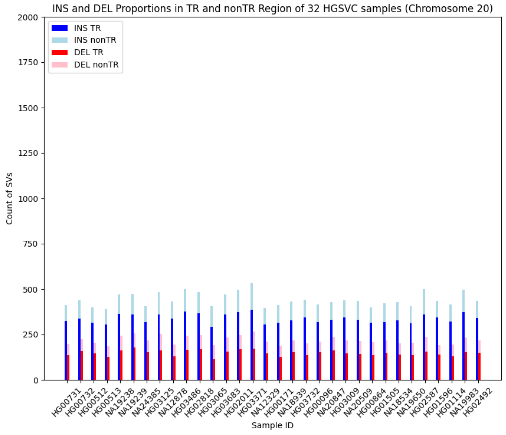

(t) Chromosome 20

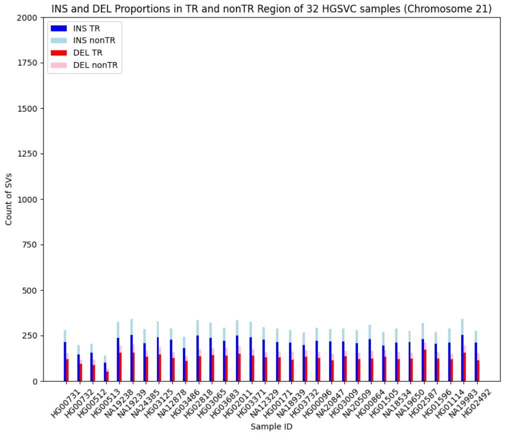

(u) Chromosome 21

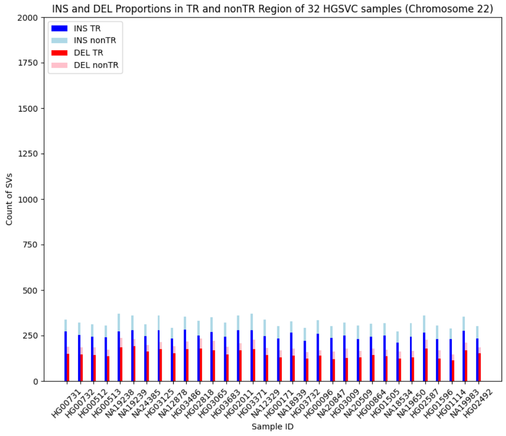

(v) Chromosome 22

Figure S5: Distribution of insertions and deletions in tandem repeat and non-tandem repeat regions in 32 *HGSVC* samples for chromosome 17 to 22

Table S3: Counts and proportions of deletions in tandem repeat regions across 32 *HGSVC* samples (transposed)

Sample	chr1	chr2	chr3	chr4	chr5	chr6	chr7	chr8	chr9	chr10	chr11	chr12	chr13	chr14	chr15	chr16	chr17	chr18	chr19	chr20	chr21	chr22
HG00731	599(60.27%)	690(67.54%)	469(62.05%)	669(60.24%)	533(62.29%)	544(56.62%)	580(64.48%)	461(65.08%)	332(59.03%)	436(70.87%)	389(64.01%)	400(71.00%)	334(64.07%)	225(60.00%)	243(63.79%)	263(72.62%)	357(72.83%)	257(70.42%)	308(71.75%)	197(69.54%)	154(77.92%)	188(79.79%)
HG00732	622(61.41%)	663(69.23%)	436(61.01%)	659(64.64%)	519(62.24%)	586(59.90%)	623(67.58%)	456(64.25%)	327(59.33%)	458(71.40%)	360(59.72%)	407(71.25%)	350(67.71%)	248(58.47%)	229(62.01%)	311(75.88%)	355(71.55%)	267(69.29%)	310(76.77%)	225(71.11%)	116(81.03%)	186(78.49%)
HG00512	599(61.27%)	702(68.95%)	435(59.08%)	593(60.20%)	501(62.48%)	564(59.22%)	603(65.17%)	424(65.57%)	351(57.26%)	437(70.25%)	395(62.03%)	410(65.61%)	310(65.16%)	244(63.93%)	213(63.85%)	306(75.16%)	347(71.75%)	260(66.92%)	288(71.18%)	206(70.38%)	116(76.72%)	185(77.83%)
HG00513	590(59.15%)	688(67.15%)	456(59.87%)	650(62.46%)	503(61.63%)	604(59.44%)	606(66.01%)	459(66.88%)	366(58.74%)	416(67.55%)	397(63.98%)	403(70.72%)	346(68.79%)	238(60.92%)	235(61.70%)	320(73.75%)	360(74.44%)	279(69.53%)	305(73.77%)	184(69.02%)	68(75.00%)	173(78.61%)
NA19238	695(60.86%)	834(69.54%)	558(59.14%)	702(61.68%)	627(62.04%)	661(61.72%)	710(67.89%)	549(69.03%)	405(59.51%)	492(73.17%)	453(61.36%)	487(70.02%)	349(66.19%)	279(61.65%)	269(64.31%)	355(71.55%)	417(72.90%)	282(69.86%)	404(74.01%)	244(66.39%)	196(80.10%)	236(77.97%)
NA19239	713(61.29%)	841(68.13%)	555(61.08%)	768(63.41%)	646(63.47%)	714(59.38%)	676(64.64%)	531(64.97%)	383(61.62%)	463(72.57%)	467(62.95%)	514(68.48%)	368(67.39%)	265(66.04%)	259(66.41%)	378(75.66%)	403(69.48%)	328(70.12%)	403(71.22%)	256(69.53%)	201(78.11%)	230(82.61%)
NA24385	601(61.73%)	718(66.71%)	480(64.17%)	636(62.26%)	546(63.00%)	588(59.35%)	579(66.84%)	482(64.94%)	299(59.87%)	460(70.87%)	373(62.19%)	396(67.93%)	350(69.14%)	242(64.05%)	212(62.26%)	287(75.96%)	366(73.49%)	244(67.62%)	303(69.97%)	219(68.95%)	164(81.10%)	199(80.90%)
HG03125	682(63.34%)	843(66.67%)	542(60.15%)	761(62.55%)	598(61.54%)	689(60.08%)	698(68.19%)	559(65.30%)	428(59.35%)	480(73.75%)	464(59.69%)	471(66.03%)	365(67.12%)	264(62.12%)	243(60.91%)	374(70.05%)	438(71.69%)	321(69.78%)	378(72.49%)	252(63.89%)	186(78.49%)	214(81.78%)
NA12878	608(59.87%)	697(66.86%)	461(61.17%)	594(62.29%)	545(63.12%)	554(59.21%)	612(67.65%)	442(64.93%)	302(58.94%)	427(69.79%)	365(60.55%)	394(68.53%)	328(64.94%)	225(62.22%)	216(62.96%)	295(70.85%)	313(75.08%)	271(69.37%)	321(73.52%)	194(67.01%)	160(78.75%)	192(79.17%)
HG03486	704(59.94%)	884(68.89%)	535(58.69%)	726(61.02%)	662(60.57%)	649(58.55%)	734(68.12%)	567(67.72%)	375(58.67%)	490(69.80%)	466(60.09%)	511(67.91%)	389(65.29%)	297(61.95%)	277(64.26%)	373(71.04%)	422(72.04%)	316(68.67%)	437(73.23%)	245(67.75%)	135(80.74%)	216(80.56%)
HG02818	705(60.99%)	838(67.42%)	568(61.27%)	713(60.31%)	630(63.65%)	697(57.68%)	734(64.85%)	559(66.55%)	404(57.69%)	470(72.34%)	443(61.85%)	486(70.16%)	368(64.67%)	288(59.03%)	253(62.05%)	370(72.70%)	398(72.86%)	295(69.83%)	390(73.08%)	248(68.14%)	176(76.70%)	234(75.64%)
HG03065	706(60.20%)	862(68.56%)	562(59.25%)	750(61.07%)	653(62.33%)	660(57.88%)	803(65.38%)	579(65.63%)	411(60.83%)	495(68.89%)	433(65.36%)	505(69.31%)	379(64.64%)	299(62.54%)	285(58.25%)	361(71.19%)	428(74.53%)	319(69.59%)	431(74.25%)	192(59.90%)	177(81.35%)	221(76.02%)
HG03683	617(59.97%)	735(67.62%)	493(62.27%)	664(61.45%)	513(62.18%)	605(59.67%)	662(61.63%)	382(60.21%)	344(61.05%)	446(64.57%)	387(62.79%)	418(67.94%)	345(66.38%)	252(62.30%)	261(57.85%)	316(70.57%)	375(71.73%)	239(69.46%)	343(73.76%)	233(66.95%)	182(76.92%)	187(77.54%)
HG02011	684(62.57%)	915(66.34%)	584(58.05%)	740(60.54%)	664(60.09%)	666(58.86%)	661(64.75%)	579(66.15%)	371(59.03%)	515(69.32%)	448(60.26%)	479(67.64%)	395(66.33%)	292(63.01%)	269(57.99%)	345(73.04%)	445(72.58%)	321(62.62%)	405(69.14%)	247(68.83%)	190(77.89%)	207(81.16%)
HG03371	694(60.37%)	830(68.43%)	590(61.52%)	754(62.47%)	647(62.75%)	661(61.12%)	701(63.48%)	559(66.55%)	387(57.88%)	516(69.77%)	472(60.80%)	526(65.78%)	367(64.58%)	283(62.89%)	265(59.62%)	349(69.63%)	416(68.75%)	315(68.25%)	375(71.20%)	267(64.79%)	175(80.00%)	227(76.65%)
NA12329	606(60.40%)	729(67.76%)	471(64.33%)	638(60.34%)	516(61.05%)	586(61.09%)	612(63.07%)	468(67.95%)	326(57.67%)	455(65.27%)	403(58.31%)	430(66.74%)	314(66.24%)	271(57.93%)	218(59.63%)	282(70.57%)	318(72.01%)	282(68.44%)	331(73.11%)	211(69.67%)	160(81.25%)	181(78.45%)
HG00171	596(61.24%)	718(66.85%)	483(62.73%)	675(62.67%)	505(63.17%)	592(57.77%)	655(61.83%)	464(66.38%)	335(59.40%)	476(67.44%)	405(62.22%)	408(69.12%)	348(62.64%)	254(59.05%)	207(61.35%)	297(70.70%)	358(69.27%)	274(71.90%)	315(71.42%)	188(68.08%)	162(80.86%)	168(77.38%)
NA18939	614(59.61%)	713(67.74%)	485(58.35%)	629(62.00%)	480(64.38%)	571(62.35%)	656(63.41%)	431(68.45%)	327(55.35%)	418(68.66%)	404(61.38%)	409(65.77%)	334(64.07%)	243(64.19%)	243(53.91%)	290(68.96%)	365(70.14%)	256(65.62%)	295(71.86%)	219(68.95%)	161(73.29%)	180(77.22%)
HG03732	616(60.71%)	744(67.88%)	447(57.27%)	702(63.11%)	551(63.16%)	621(60.06%)	642(62.46%)	445(66.97%)	336(57.74%)	468(70.51%)	374(62.57%)	414(68.36%)	339(64.90%)	234(58.97%)	242(59.50%)	334(72.15%)	369(73.17%)	245(66.12%)	313(72.84%)	200(68.00%)	170(78.82%)	159(77.36%)
HG00096	602(60.63%)	704(67.05%)	502(58.37%)	644(61.18%)	549(66.12%)	586(57.85%)	602(64.78%)	463(66.52%)	298(58.38%)	451(69.40%)	383(59.79%)	415(67.71%)	370(64.05%)	231(56.71%)	235(57.44%)	328(68.29%)	326(71.16%)	277(66.06%)	322(74.53%)	211(72.03%)	158(80.38%)	169(82.84%)
NA20847	609(60.10%)	716(67.32%)	528(61.55%)	643(61.28%)	551(60.98%)	586(59.73%)	565(63.19%)	465(67.10%)	345(54.49%)	444(69.59%)	370(60.00%)	418(68.66%)	358(67.32%)	252(57.93%)	254(59.45%)	310(71.29%)	362(71.55%)	271(64.94%)	311(73.95%)	237(67.93%)	148(77.70%)	161(75.15%)
HG03009	628(60.35%)	653(66.62%)	495(62.63%)	677(61.15%)	559(62.79%)	622(58.20%)	629(61.84%)	483(66.87%)	318(57.55%)	464(73.49%)	381(63.78%)	438(67.81%)	348(68.10%)	242(59.09%)	233(57.51%)	305(65.57%)	360(71.39%)	274(66.78%)	315(71.11%)	217(67.74%)	164(82.32%)	177(72.32%)
NA20509	612(61.11%)	722(66.76%)	454(62.11%)	647(63.06%)	506(62.65%)	559(59.74%)	617(64.02%)	451(67.18%)	319(56.73%)	443(66.14%)	397(59.70%)	435(68.97%)	316(65.82%)	246(56.91%)	231(55.84%)	302(69.54%)	131(76.34%)	258(65.89%)	324(73.46%)	213(67.14%)	155(77.42%)	166(78.31%)
HG00864	652(63.04%)	717(67.36%)	516(62.02%)	655(62.14%)	496(63.51%)	573(61.95%)	638(62.23%)	448(63.84%)	319(55.80%)	469(69.72%)	384(58.85%)	413(68.28%)	342(62.57%)	247(59.11%)	243(58.44%)	313(69.01%)	396(70.20%)	269(66.54%)	320(72.81%)	208(64.90%)	166(75.30%)	180(78.88%)
HG01505	598(60.70%)	727(68.36%)	468(61.11%)	639(60.25%)	556(65.28%)	538(57.62%)	641(65.83%)	445(66.74%)	332(62.35%)	447(66.22%)	382(58.90%)	404(67.82%)	357(68.90%)	229(60.70%)	233(59.23%)	302(72.18%)	318(67.30%)	261(67.82%)	290(75.52%)	217(69.12%)	169(78.11%)	171(80.12%)
NA18534	633(58.93%)	706(66.28%)	501(61.67%)	649(62.71%)	479(64.09%)	572(57.87%)	592(63.34%)	447(67.11%)	311(58.20%)	451(69.17%)	393(58.02%)	414(69.81%)	351(63.82%)	247(60.73%)	209(56.45%)	365(71.51%)	368(74.73%)	269(66.17%)	286(73.08%)	202(69.80%)	160(75.00%)	161(76.40%)
NA19650	597(60.80%)	724(68.23%)	483(61.70%)	688(59.74%)	548(64.60%)	614(57.49%)	650(64.77%)	446(69.28%)	320(56.88%)	506(70.36%)	393(57.51%)	446(69.51%)	341(65.10%)	255(58.04%)	254(58.26%)	314(70.38%)	322(72.05%)	285(72.63%)	293(70.31%)	205(65.85%)	157(78.98%)	166(78.31%)
HG02587	676(59.91%)	879(65.87%)	603(60.19%)	755(63.17%)	634(64.51%)	648(56.48%)	716(66.06%)	591(64.13%)	404(59.90%)	444(68.46%)	465(58.06%)	488(68.24%)	383(67.36%)	287(58.54%)	283(60.42%)	353(73.09%)	429(69.69%)	303(65.35%)	408(72.55%)	237(66.24%)	210(81.90%)	226(79.65%)
HG01596	610(59.84%)	687(63.46%)	486(61.73%)	602(62.13%)	489(63.39%)	586(55.63%)	648(62.35%)	415(67.47%)	327(53.52%)	468(69.01%)	366(60.93%)	415(70.12%)	343(68.51%)	251(52.99%)	244(54.91%)	288(70.49%)	352(70.73%)	274(68.25%)	305(74.42%)	193(71.50%)	160(76.87%)	168(73.81%)
HG01114	576(57.12%)	756(66.27%)	475(59.58%)	682(62.76%)	528(62.88%)	587(58.60%)	663(64.25%)	483(67.49%)	317(57.72%)	512(69.34%)	391(58.57%)	400(72.00%)	345(66.37%)	213(59.62%)	239(61.92%)	322(68.32%)	381(69.03%)	285(67.72%)	315(71.74%)	196(66.83%)	150(80.00%)	147(77.55%)
NA19983	680(61.62%)	842(65.80%)	588(58.33%)	740(60.00%)	595(60.00%)	645(55.97%)	685(65.84%)	548(66.42%)	370(57.03%)	165(68.48%)	459(63.18%)	461(68.11%)	365(66.30%)	274(59.85%)	262(58.78%)	367(73.57%)	396(72.73%)	329(70.82%)	372(73.12%)	235(64.68%)	195(80.00%)	211(80.09%)
HG02492	607(61.78%)	728(69.23%)	502(61.95%)	682(65.54%)	525(62.86%)	590(58.93%)	630(61.27%)	464(68.75%)	317(56.78%)	216(68.52%)	380(63.68%)	459(69.93%)	354(64.97%)	239(58.96%)	226(59.73%)	319(69.59%)	350(71.71%)	244(62.30%)	330(72.12%)	216(68.52%)	153(75.16%)	186(81.18%)
TR ratio mean(sd)	60.66%(1.2%)	67.4%(1.24%)	60.76%(1.77%)	61.87%(1.36%)	62.77%(1.41%)	58.94%(1.67%)	64.6%(2.02%)	66.33%(1.79%)	58.24%(1.95%)	69.37%(2.44%)	61.04%(2.06%)	68.6%(1.61%)	65.92%(1.77%)	60.33%(2.67%)	60.03%(2.92%)	71.4%(2.37%)	71.84%(2.01%)	67.96%(2.41%)	72.73%(1.61%)	67.79%(2.45%)	78.57%(2.31%)	78.43%(2.41%)
TR region ratio	3.47%	2.91%	3.31%	2.9%	4.07%	2.78%	5.09%	3.72%	3.86%	4.34%	5.18%	4.72%	3.42%	3.8%	4.34%	5.86%	8.19%	8.61%	10.76%	7.42%	7.87%	8.2%

Table S4: Counts and proportions of insertions in tandem repeat regions across 32 *HGSVC* samples (transposed)

Sample	chr1	chr2	chr3	chr4	chr5	chr6	chr7	chr8	chr9	chr10	chr11	chr12	chr13	chr14	chr15	chr16	chr17	chr18	chr19	chr20	chr21	chr22
HG00731	968(68.29%)	1007(68.12%)	737(63.91%)	838(68.62%)	747(68.54%)	853(65.77%)	873(70.22%)	655(69.77%)	535(70.28%)	681(72.10%)	673(64.93%)	711(67.09%)	593(68.47%)	351(63.25%)	326(57.06%)	481(76.09%)	508(71.85%)	407(74.94%)	479(70.98%)	411(79.32%)	278(77.34%)	339(80.24%)
HG00732	1066(66.70%)	1007(70.71%)	714(63.45%)	786(68.32%)	732(68.72%)	871(67.16%)	876(69.52%)	667(70.46%)	582(70.10%)	731(73.87%)	693(68.54%)	667(67.32%)	549(67.94%)	369(62.87%)	342(62.28%)	508(79.13%)	561(73.26%)	427(74.00%)	512(68.75%)	437(77.57%)	197(74.11%)	321(78.84%)
HG00512	1021(65.92%)	1001(68.23%)	719(60.36%)	787(68.49%)	663(69.38%)	873(67.35%)	873(70.56%)	660(71.21%)	556(69.42%)	687(71.91%)	664(65.36%)	654(65.44%)	565(71.33%)	355(67.04%)	349(61.60%)	506(78.26%)	509(74.26%)	420(73.57%)	510(71.57%)	400(78.50%)	203(77.34%)	312(77.88%)
HG00513	1017(69.03%)	1008(68.75%)	728(61.95%)	830(67.59%)	680(69.41%)	864(67.13%)	911(71.46%)	659(74.36%)	577(69.50%)	665(70.68%)	674(67.36%)	685(68.32%)	560(70.00%)	334(65.87%)	340(62.94%)	499(75.35%)	502(73.51%)	407(72.48%)	492(71.80%)	389(78.65%)	139(73.38%)	305(78.36%)
NA19238	1148(64.98%)	1184(69.17%)	836(59.09%)	940(66.17%)	844(65.99%)	1021(66.21%)	1009(69.67%)	770(70.00%)	648(68.36%)	775(69.81%)	775(65.29%)	755(66.09%)	627(69.54%)	397(63.22%)	380(60.79%)	569(75.40%)	603(72.14%)	408(70.83%)	591(69.20%)	471(77.07%)	326(72.70%)	370(74.05%)
NA19239	1195(63.60%)	1135(70.48%)	855(62.69%)	909(65.35%)	829(69.12%)	954(67.82%)	995(68.04%)	780(69.62%)	640(67.97%)	767(72.10%)	738(65.31%)	737(65.13%)	611(70.87%)	395(61.27%)	370(61.35%)	581(74.70%)	611(71.36%)	431(67.98%)	592(68.92%)	475(75.58%)	340(74.71%)	361(77.56%)
NA24385	997(67.40%)	966(68.74%)	764(63.87%)	833(67.47%)	703(69.84%)	870(68.28%)	880(69.86%)	634(70.50%)	560(71.07%)	723(74.00%)	670(67.01%)	678(66.67%)	569(69.77%)	339(63.72%)	334(62.27%)	487(79.88%)	498(72.69%)	398(73.37%)	487(69.61%)	406(78.57%)	285(73.33%)	313(79.23%)
HG03125	1141(67.22%)	1160(68.19%)	888(61.04%)	890(64.61%)	861(67.13%)	967(66.18%)	1029(67.83%)	779(69.06%)	646(68.89%)	799(72.22%)	772(67.10%)	724(64.92%)	591(69.88%)	397(61.21%)	390(60.26%)	549(75.05%)	645(70.54%)	440(70.68%)	592(69.43%)	485(74.23%)	328(73.17%)	359(77.99%)
NA12878	990(65.76%)	697(68.59%)	712(60.67%)	772(68.13%)	714(69.19%)	877(66.36%)	853(68.45%)	655(69.77%)	541(71.16%)	680(72.94%)	687(65.06%)	671(66.62%)	552(69.57%)	354(63.84%)	301(60.13%)	490(76.99%)	506(73.72%)	433(73.90%)	475(69.47%)	432(78.24%)	290(78.28%)	293(79.54%)
HG03486	1210(66.12%)	1197(69.01%)	839(59.95%)	935(65.24%)	792(64.90%)	980(67.24%)	999(70.17%)	792(67.17%)	649(69.80%)	789(73.13%)	766(65.79%)	754(64.46%)	627(69.22%)	427(63.93%)	371(57.69%)	536(74.25%)	642(71.96%)	433(69.75%)	579(71.13%)	501(75.25%)	242(75.21%)	353(79.89%)
HG02818	1144(65.82%)	1119(68.10%)	811(58.94%)	914(67.07%)	804(66.42%)	991(70.03%)	987(69.40%)	798(68.67%)	636(70.75%)	730(70.82%)	757(66.18%)	712(65.03%)	627(70.49%)	423(63.36%)	386(60.88%)	569(73.46%)	608(70.89%)	432(68.75%)	606(71.23%)	485(75.67%)	336(74.70%)	330(76.06%)
HG03065	1226(64.11%)	1194(66.58%)	875(62.06%)	925(65.41%)	812(67.12%)	1000(67.40%)	1012(65.51%)	816(68.73%)	662(68.13%)	808(70.67%)	740(65.14%)	773(65.72%)	680(66.47%)	436(61.95%)	420(57.62%)	594(73.74%)	648(70.37%)	426(69.02%)	622(68.33%)	405(72.35%)	320(74.06%)	352(76.99%)
HG03683	1072(64.37%)	1074(66.85%)	764(60.75%)	852(68.90%)	677(68.54%)	886(67.04%)	884(68.44%)	594(64.65%)	603(69.65%)	707(68.46%)	699(65.38%)	671(65.72%)	573(69.12%)	372(64.52%)	341(57.77%)	489(75.26%)	572(68.18%)	413(73.11%)	520(70.58%)	471(76.49%)	292(75.68%)	323(75.85%)
HG02011	1137(64.82%)	1206(66.83%)	877(59.97%)	896(65.85%)	842(67.58%)	983(67.04%)	1005(69.65%)	798(69.55%)	653(70.60%)	788(69.53%)	756(65.34%)	756(64.68%)	622(67.20%)	459(63.62%)	418(57.68%)	547(73.85%)	629(69.95%)	450(68.67%)	623(68.39%)	497(74.80%)	333(75.34%)	361(77.27%)
HG03371	1216(62.91%)	1210(68.02%)	850(61.41%)	900(64.00%)	842(67.23%)	957(67.96%)	1014(68.74%)	811(69.79%)	632(67.56%)	789(68.95%)	769(64.89%)	734(66.85%)	658(68.24%)	432(60.19%)	403(56.58%)	572(72.55%)	667(70.94%)	470(70.00%)	643(67.85%)	531(72.88%)	325(73.54%)	369(75.34%)
NA12329	1028(65.76%)	1073(68.03%)	761(63.21%)	807(68.03%)	722(66.76%)	906(66.67%)	876(69.75%)	634(72.08%)	558(70.07%)	699(69.81%)	693(65.66%)	688(66.13%)	564(69.50%)	372(60.22%)	372(58.60%)	519(72.25%)	582(71.65%)	423(72.10%)	508(68.70%)	397(76.85%)	295(77.20%)	337(73.57%)
HG00171	1069(65.95%)	1054(67.94%)	741(59.51%)	807(64.19%)	683(67.35%)	889(63.78%)	914(67.17%)	675(72.00%)	335(59.40%)	720(72.08%)	681(66.81%)	679(64.06%)	580(68.97%)	372(63.44%)	343(58.31%)	493(71.59%)	545(71.56%)	419(74.98%)	500(69.40%)	412(76.21%)	289(74.09%)	302(77.12%)
NA18939	1042(66.51%)	1058(67.87%)	739(66.14%)	803(68.24%)	687(67.54%)	859(67.29%)	941(70.56%)	717(71.83%)	327(55.35%)	687(71.91%)	695(65.73%)	661(67.93%)	567(68.04%)	336(60.71%)	366(58.73%)	499(67.59%)	544(71.53%)	406(73.32%)	515(70.23%)	433(78.13%)	278(76.27%)	327(81.35%)
HG03732	1103(65.00%)	1046(66.16%)	664(57.83%)	852(67.14%)	761(67.41%)	922(64.43%)	893(67.52%)	675(70.37%)	336(57.74%)	737(69.74%)	718(64.63%)	687(64.69%)	549(69.40%)	346(64.41%)	356(58.73%)	564(73.96%)	562(70.11%)	426(69.73%)	499(65.93%)	440(78.18%)	267(73.78%)	291(77.94%)
HG00096	1000(66.60%)	1053(67.14%)	770(61.20%)	823(68.29%)	734(67.57%)	880(65.23%)	905(69.50%)	636(70.40%)	574(70.73%)	756(70.53%)	680(65.00%)	674(65.87%)	554(69.01%)	375(62.40%)	350(61.71%)	530(72.83%)	567(71.25%)	410(70.98%)	509(67.94%)	429(76.89%)	286(76.24%)	320(77.85%)
NA20847	1039(68.91%)	1050(67.62%)	740(62.03%)	850(68.82%)	727(65.45%)	965(66.53%)	881(68.90%)	670(68.81%)	559(69.90%)	721(71.71%)	681(62.96%)	700(66.29%)	608(67.24%)	359(63.79%)	375(62.93%)	520(74.62%)	536(71.83%)	411(73.26%)	505(66.93%)	439(78.13%)	289(74.75%)	304(75.63%)
HG03009	1057(65.09%)	977(65.71%)	712(61.38%)	812(66.13%)	742(67.39%)	947(66.74%)	924(69.37%)	680(70.15%)	582(69.92%)	294(72.79%)	715(65.59%)	694(67.44%)	589(68.96%)	353(59.44%)	350(55.43%)	524(75.38%)	233(63.95%)	408(73.21%)	506(66.98%)	436(75.92%)	278(74.46%)	316(77.53%)
NA20509	1106(65.55%)	1050(67.62%)	782(60.23%)	797(66.70%)	690(66.81%)	875(66.86%)	865(69.02%)	621(70.85%)	561(69.89%)	713(71.55%)	690(65.65%)	675(64.74%)	559(69.23%)	373(63.76%)	355(59.73%)	525(73.71%)	535(72.34%)	415(73.08%)	491(68.24%)	399(78.95%)	309(75.08%)	319(78.05%)
HG00864	1057(64.62%)	1069(67.44%)	744(60.71%)	860(66.05%)	653(67.93%)	927(66.03%)	925(67.68%)	645(72.40%)	568(69.39%)	709(70.32%)	705(66.24%)	693(65.04%)	595(67.06%)	358(62.54%)	322(59.34%)	520(75.96%)	525(72.09%)	414(73.21%)	508(68.12%)	405(77.04%)	289(73.10%)	318(78.05%)
HG01505	1037(65.00%)	1024(67.68%)	791(61.12%)	815(67.12%)	714(66.53%)	886(67.21%)	920(70.00%)	645(72.40%)	571(68.93%)	703(70.27%)	678(63.72%)	678(65.01%)	583(68.97%)	374(62.54%)	320(57.19%)	503(73.58%)	636(72.19%)	409(73.08%)	476(69.76%)	429(76.92%)	268(73.10%)	273(76.92%)
NA18534	1072(66.32%)	1032(68.12%)	874(68.02%)	823(67.12%)	685(66.53%)	916(66.05%)	941(66.31%)	685(70.51%)	587(68.90%)	843(69.99%)	712(64.33%)	700(62.00%)	578(66.90%)	445(61.79%)	383(56.64%)	512(72.66%)	512(72.09%)	400(72.75%)	616(66.56%)	500(72.40%)	319(72.12%)	359(77.07%)
NA19650	1065(65.63%)	1032(68.12%)	917(63.43%)	829(67.55%)	743(65.81%)	1078(66.60%)	1025(66.43%)	819(65.89%)	673(69.64%)	434(79.03%)	813(63.59%)	746(63.94%)	645(66.83%)	377(62.07%)	304(75.33%)	573(73.82%)	616(70.91%)	448(69.19%)	359(77.07%)	341(69.12%)	354(78.24%)	335(77.99%)
HG02587	1213(62.41%)	1179(66.84%)	877(57.01%)	913(64.84%)	846(66.02%)	917(63.47%)	875(66.31%)	644(70.96%)	680(69.85%)	680(69.85%)	674(63.75%)	693(66.96%)	581(66.83%)	680(69.85%)	430(57.21%)	495(73.74%)	553(69.44%)	434(71.72%)	416(77.12%)	271(74.91%)	304(75.33%)	303(76.90%)
HG01596	1077(64.25%)	1017(67.35%)	741(58.04%)	829(67.55%)	691(66.71%)	917(63.47%)	908(68.73%)	690(71.74%)	573(69.64%)	720(69.31%)	708(65.38%)	713(66.99%)	573(66.84%)	368(62.07%)	360(61.39%)	526(74.21%)	540(68.15%)	405(71.72%)	496(66.32%)	496(77.12%)	290(73.10%)	290(80.00%)
HG01114	1031(65.28%)	998(67.94%)	779(60.59%)	805(66.09%)	705(67.39%)	912(65.89%)	784(69.01%)	682(68.23%)	580(68.10%)	784(69.01%)	758(65.30%)	765(64.89%)	611(69.35%)	404(65.84%)	430(57.21%)	569(74.13%)	616(70.59%)	456(71.58%)	588(69.12%)	436(78.24%)	341(74.12%)	354(78.24%)
NA19983	1258(65.90%)	1182(66.92%)	901(63.60%)	876(64.95%)	825(68.36%)	993(67.54%)	978(70.00%)	795(70.88%)	663(69.80%)	293(72.68%)	758(65.30%)	713(66.90%)	611(69.35%)	368(61.13%)	329(55.65%)	526(74.13%)	548(70.26%)	411(71.94%)	492(66.32%)	496(77.12%)	275(76.36%)	303(76.90%)
HG02492	1108(64.98%)	1036(68.24%)	731(63.38%)	819(66.91%)	708(68.73%)	935(68.30%)	857(66.39%)	690(71.74%)	568(69.39%)	216(68.52%)	679(63.79%)	686(64.83%)	572(68.77%)	368(61.13%)	360(61.39%)	535(74.31%)	548(70.26%)	411(71.94%)	492(66.32%)	436(78.24%)	275(76.36%)	303(76.90%)
TR ratio mean(sd)	65.65%(1.51%)	67.86%(1.08%)	61.11%(1.76%)	66.71%(1.43%)	67.49%(1.24%)	66.78%(1.31%)	68.81%(1.49%)	70.17%(1.77%)	69.16%(1.31%)	71.06%(1.47%)	65.38%(1.17%)	65.70%(1.31%)	68.71%(1.31%)	62.68%(1.85%)	58.97%(2.42%)	74.81%(1.79%)	71.12%(1.92%)	71.79%(1.98%)	69.12%(1.49%)	76.58%(1.88%)	74.85%(1.62%)	77.38%(1.86%)
TR region ratio	3.47%	2.91%	3.31%	2.9%	4.07%	2.78%	5.09%	3.72%	3.86%	4.34%	5.18%	4.72%	3.42%	3.8%	4.34%	5.86%	8.19%	8.61%	10.76%	7.42%	7.87%	8.2%

1.4 Non-uniform distribution in SV lengths

Figure S6 illustrates the empirical length distribution of SVs for HG002 and 15 *Cell* samples. The figure is divided into four subfigures, with each representing distinct length ranges of the SVs.
A large-scale study involving 3,622 Icelandic individuals(Beyter et al., 2021) identified prominent peaks in the SV length distribution at approximately 300 bp, 2500 bp, and 6000 bp. Additionally, further analysis of publicly available SV data from comprehensive characterizations of both HG002 and the 15 *Cell* samples corroborate these findings, revealing similar distribution patterns across the datasets.

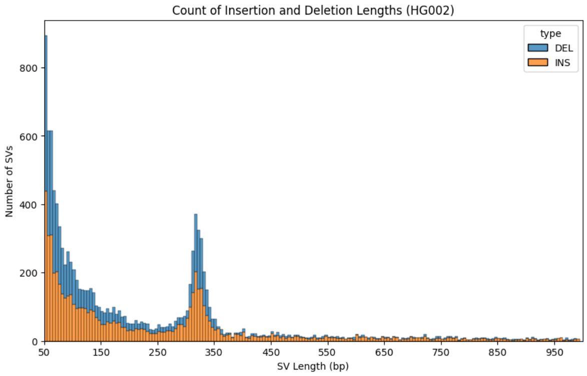

(a) Empirical length distribution of SVs in *HG002* (50-1000 bp).

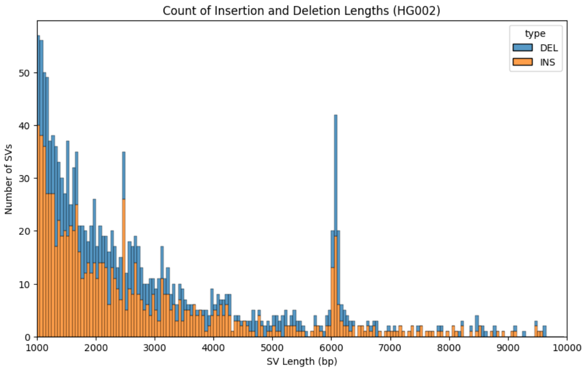

(b) Empirical length distribution of SVs in *HG002* (1000-10000 bp)

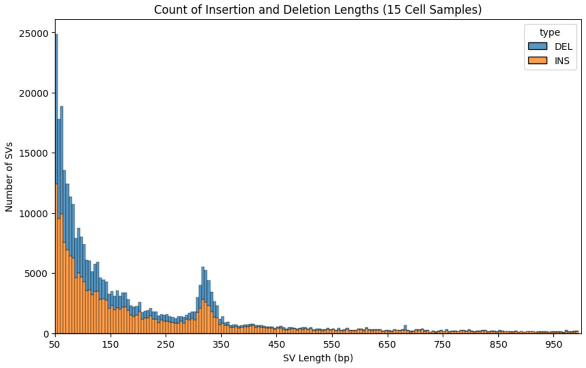

(c) Empirical length distribution of SVs across the 15 *Cell* samples (50-1000 bp).

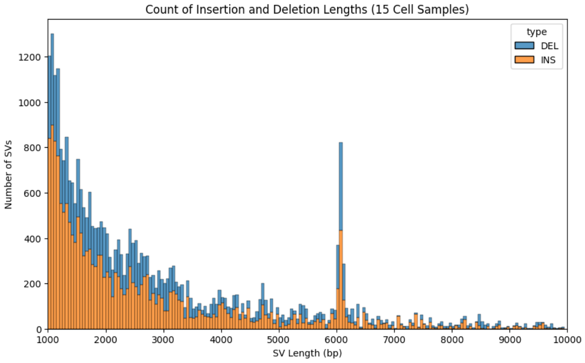

(d) Empirical length distribution of SVs across the 15 *Cell* samples (1000-10000 bp)

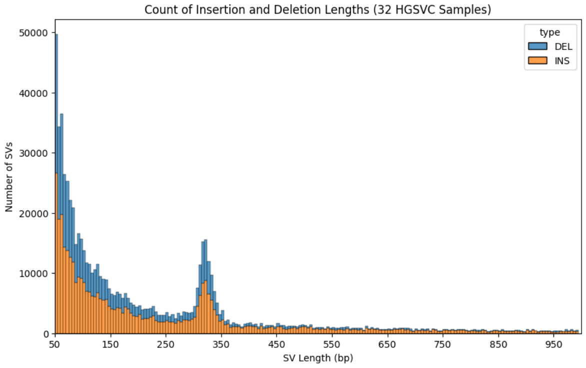

(e) Empirical length distribution of SVs across the 32 *HGSVC* samples (50-1000 bp).

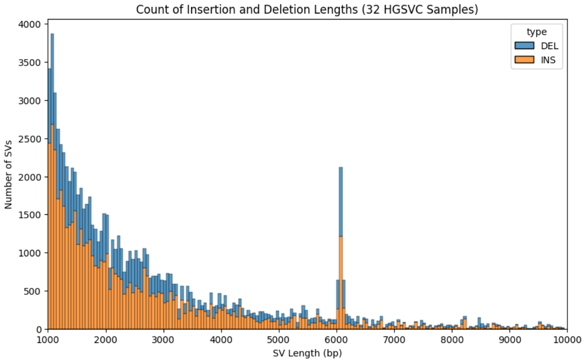

(f) Empirical length distribution of SVs across the 32 *HGSVC* samples (1000-10000 bp)

Figure S6: The empirical length distribution of *HG002*, 15 *Cell* samples and 32 *HGSVC* samples

1.5 Population-Level SV Annotations of gnomAD v4.1

The gnomAD v4.1 (Collins et al., 2020) VCF file provides comprehensive population allele frequencies and genotype statistics of population-level SVs. Below are the key annotations:

Code	Population	Geographical/Cultural Origin
afr	African/African American	Sub-Saharan African ancestry
ami	Amish	Pennsylvania Dutch/Amish community
amr	Admixed American	Latin American with mixed ancestry
asj	Ashkenazi Jewish	Central/Eastern European Jewish
eas	East Asian	Chinese, Japanese, Korean
fin	Finnish	Finnish-specific variants
mid	Middle Eastern	Arab, Persian, Turkish populations
nfe	Non-Finnish European	European (excluding Finnish)
rmi	Native American	Indigenous American populations
sas	South Asian	Indian, Pakistani, Bangladeshi

Table S5: Population codes and descriptions in gnomAD SV v4.1

Field	Description
`AN`	Total number of alleles in population (chromosome count × sample size)
`AC`	Count of alternate alleles observed in population
`AF`	Alternate allele frequency (calculated as AC/AN, range 0-1)
`N_BI_GENOS`	Total number of biallelic genotypes (diploid samples × 2)
`N_HOMREF`	Count of homozygous reference genotypes
`N_HET`	Count of heterozygous genotypes
`N_HOMALT`	Count of homozygous alternate genotypes
`FREQ_HOMREF`	Proportion of homozygous reference genotypes (N_HOMREF/AN)
`FREQ_HET`	Proportion of heterozygous genotypes (N_HET/AN)
`FREQ_HOMALT`	Proportion of homozygous alternate genotypes (N_HOMALT/AN)

Table S6: Key population-level SV annotation fields in gnomAD v4.1

Code	Description
AN	Allele Number (total number of alleles)
AC	Allele Count (count of specific alleles)
AF	Allele Frequency (proportion of specific alleles)
N_BI_GENOS	Number of Biallelic Genotypes
N_HOMREF	Number of Homozygous Reference Genotypes
N_HET	Number of Heterozygous Genotypes
N_HOMALT	Number of Homozygous Alternate Genotypes
FREQ_HOMREF	Frequency of Homozygous Reference Genotypes
FREQ_HET	Frequency of Heterozygous Genotypes
FREQ_HOMALT	Frequency of Homozygous Alternate Genotypes

Table S7: Genotype frequency metrics in gnomAD SV v4.1

Key Features:

- **Sex stratification**: Append `_XX` (female) or `_XY` (male) to population codes (e.g., `AF_nfe_XX`).
- **Quality flags**: Confidence metrics like `LOW_CONFIDENCE_REPETITIVE_LARGE_DUP` indicate unreliable calls.
- **Technical metadata**: Includes PCR-specific metrics (e.g., `PCRMINUS_NCR`).

2 Introduction to BVSim

BVSim facilitates both uniform and non-uniform SV generation. Users can input any reference genome, and BVSim learns the SV position distribution from user-provided BED files. It offers seven modes of sequence simulation, including exact mode, uniform mode, uniform-parallel mode, CSV mode, mimic mode, wave mode, and wave-region mode.

2.1 Exact/uniform/uniform-parallel

The **exact mode** performs comprehensive variant screening for the user-input variant table, automatically excluding any overlapping SVs or small variants based on their genomic coordinates (sorted from smallest to largest position). Note that this quality control process may require additional computation time in BVSim version 1.0.0. In **uniform mode**, the simulator randomly samples new variant positions from available genomic regions that have not been previously assigned to other variants. The **uniform-parallel mode** extends this functionality through a segmented approach:

- The reference sequence is partitioned into M segments as in mimic/wave/wage-region mode. Variants are sampled independently within each segment.
- The terminal segment retaining residual length when the sequence length is not divisible by the bin size.
- Cross-segment variants are systematically excluded.

2.2 CSV Definitions

BVSim can simulate 18 types of CSVs randomly without spatial assumption, including:

- ID1: Tandem Inverted Duplication (TanInvDup)
- ID2: Dispersed Inverted Duplication (DisInvDup)
- ID3: Dispersed Duplication (DisDup)
- ID4: Inversion with 5' or 3' Flanking Deletion (DEL+INV/INV+DEL)
- ID5: 5' Deletion and Dispersed Inverted Duplication (DEL+DisInvDup)
- ID6: 5' Deletion and Dispersed Duplication (DEL+DisDup)
- ID7: Tandem Duplication and 3' Deletion (TanDup+DEL)
- ID8: Tandem Inverted Duplication and 3' Deletion (TanInvDup+DEL)
- ID9: Tandem Duplication, Deletion and Inversion (TanDup+DEL+INV)
- ID10: Tandem Inverted Duplication, Deletion and Inversion (TanInvDup+DEL+INV)
- ID11: Paired-Deletion Inversion (DEL+INV+DEL)
- ID12: Inversion with 5' Flanking Duplication (DUP+INV)
- ID13: Inversion with 3' Flanking Duplication (INV+DUP)
- ID14: Paired-Duplication Inversion (DUP+INV+DUP)
- ID15: Inversion with 5' Flanking Duplication and 3' Flanking Deletion (DUP+INV+DEL)
- ID16: Inversion with 5' Flanking Deletion and 3' Flanking Duplication (DEL+INV+DUP)
- ID17: Inverted Duplication with Flanking Triplication (DupTripDup-INV)
- ID18: Insertion with Deletion (INSdel)

We include the above IDs in the output VCF files for simplicity.

2.3 Mimic/wave/wave-region: empirical vs probability

This section compares two distinct approaches for SV generation in the mimic mode, wave mode, and wave-region mode: empirical sample-matching versus probability-based simulation. The key distinction lies in how insertion/deletion counts are allocated across genomic segments.

The simulation workflow implements:

1. **Flag '-mode empirical'**:
   - Directly uses observed counts/means per bin from input samples
   - Preserves the exact spatial distribution of input variants
2. **Flag '-mode probability'**:
   - Converts input counts to segment-specific probabilities
   - Redistributes total variants (calculated from the sample if '-sum' flag or assigned by the user) via multinomial sampling

2.4 Implementation Details for Sampling

2.4.1 Position Sampling

Within each segment S_j, available positions are partitioned into:

- TR regions: $S_j^{\mathrm{TR}} = S_j \cap R_{\mathrm{user}}$
- Non-TR regions: $S_j^{\mathrm{nonTR}} = S_j \setminus S_j^{\mathrm{TR}}$

The start position x is sampled according to:

$$x \sim \begin{cases} \mathcal{U}(S_j) & \text{(wave mode)} \\ \begin{cases} \mathcal{U}(S_j^{\mathrm{TR}}) & \text{with prob. } p_{\mathrm{TR}} \\ \mathcal{U}(S_j^{\mathrm{nonTR}}) & \text{with prob. } 1 - p_{\mathrm{TR}} \end{cases} & \text{(wave-region mode)} \end{cases} \tag{1}$$

where $\mathcal{U}(\cdot)$ denotes uniform sampling over the specified region; p_{TR} is the user-defined TR region preference probability. Segments with all positions blocked are skipped (with warnings).

2.4.2 Length Sampling

$$l \sim P_t(l) \text{ with } l_{t,\min} \leq l \leq l_{t,\max} \tag{2}$$

where $l_{t,\min}, l_{t,\max}$ are users' input, which can truncate the empirical probability mass function $P_t(l)$ of samples' SV lengths.

2.4.3 Parallel Execution

Segments are processed in parallel according to their computational complexity, prioritized by a weighted score ρ_j:

$$\rho_j = k_j^{\mathrm{del}} + k_j^{\mathrm{ins}} + \bar{l}_j \tag{3}$$

where k_j^{del} and k_j^{ins} represent the simulated deletion and insertion counts in segment S_j, respectively. $\bar{l}_j$ denotes the length of unblocked regions within S_j

Higher ρ_j values indicate segments requiring more computational resources, which are scheduled for priority processing. This dynamic load-balancing approach optimizes parallel efficiency by:

- Distributing computationally intensive segments first;
- Accounting for both variant counts and region accessibility;

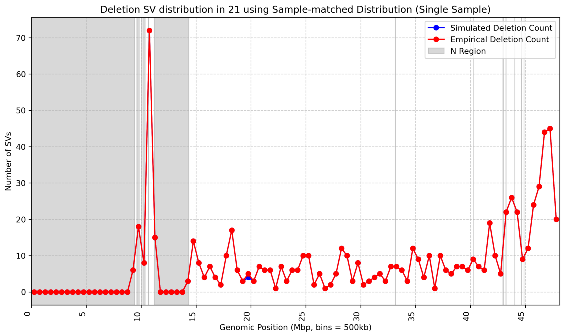

(a) Empirical mode (hg19 single-sample)

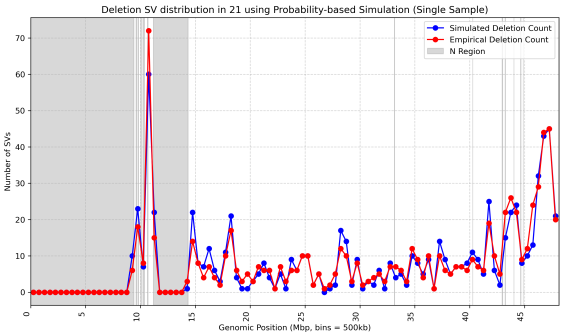

(b) Probability mode (hg19 single-sample)

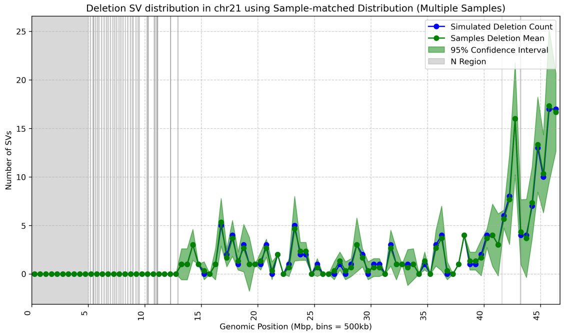

(c) Empirical mode (hg38 multi-sample)

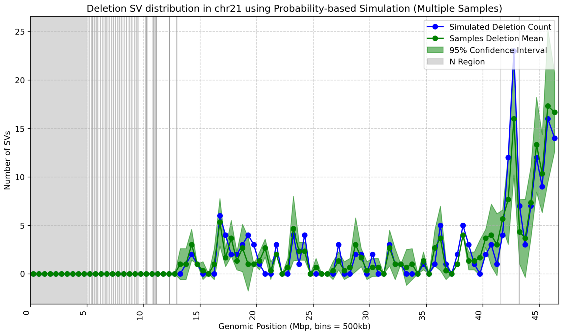

(d) Probability mode (hg38 multi-sample)

Figure S7: Comparison of deletion distribution generation methods on chromosome 21. Empirical mode (a,c) directly uses observed counts from input samples, while probability mode (b,d) redistributes variants according to segment-specific probabilities. Single-sample (a,b) versus multi-sample (c,d) comparisons demonstrate method consistency across datasets.

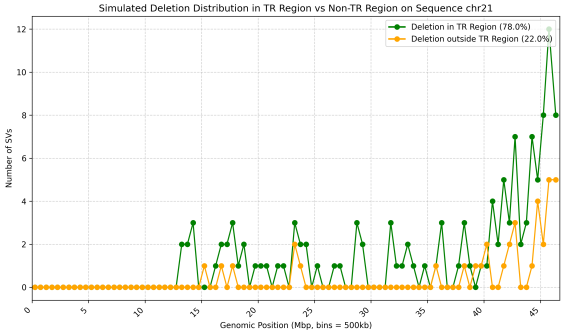

(a) Deletion distribution (p_del_region=0.8, L=500kbp)

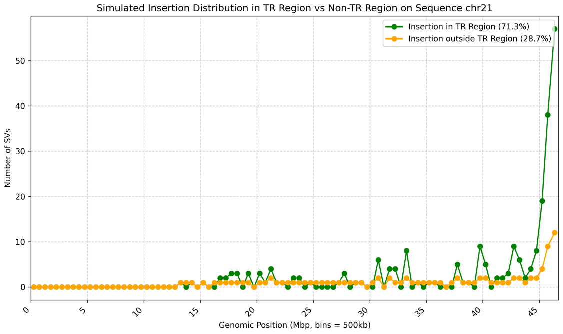

(b) Insertion distribution (p_ins_region=0.7, L=500kbp)

Figure S8: Simulated SV distributions in wave-region mode showing TR enrichment (chr21/hg38). (a) Deletions and (b) insertions demonstrate increased density in user-defined tandem repeat regions with 500kbp bin size.

2.5 Comparison with VarSim and Mutation-Simulator (MTS)

2.5.1 Methodological Differences in SV Simulation Approaches

Figure S9 illustrates the sampling probability spaces for different simulators using chromosome 10. Figure S9(a) highlights the fixed sampling constraints of VarSim (Mu et al., 2015). Figure S9(b) shows the uniform deletion distribution of SVs from the Mutation-Simulator (MTS) (Kühl et al., 2021). Figure S9(c) presents the flexible sampling approach for non-uniform insertions with uniform length distribution by MTS. Figure S9(d) captures the non-uniform distribution in both location and length produced by BVSim. This figure emphasizes the distinct methodologies among the simulators, particularly BVSim's ability to reflect more realistic patterns.

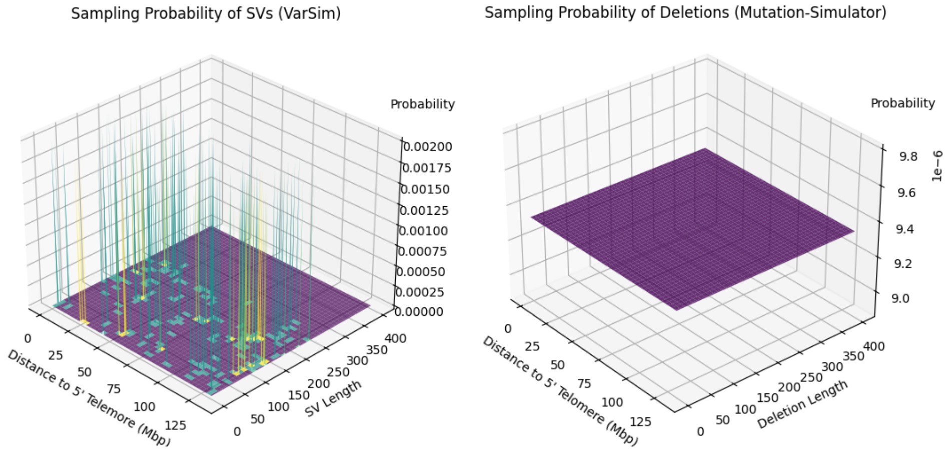

(a) Sampling probability of SVs (VarSim) (b) Sampling probability of deletions (MTS)

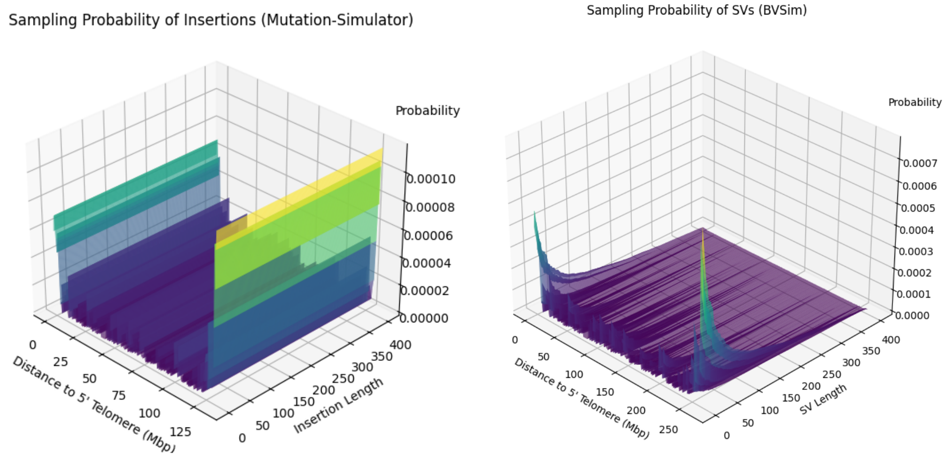

(c) Sampling probability of insertions (MTS) (d) Sampling probability of SVs (BVSim)

Figure S9: The illustration of sampling probability spaces for different simulators using chromosome 10 as an example. (a) The sampling probability space for SVs from fixed regions using VarSim. (b) The uniform sampling probability space for SVs using MTS across chromosome 10 (133.80 million base pairs, Mbp). (c) The flexible sampling probability space for insertions using MTS across chromosome 10. (d) The non-uniform sampling probability space for SVs using BVSim across chromosome 10

2.5.2 Comparative Analysis of SV Length Distributions Across Simulated Data

In Figure S10, we visualize the original F1 scores corresponding to Fig.1(b-c). In Figure S11, the length distributions of simulated SVs reveal distinct patterns across different simulation approaches. Detailed quantiles of simulated lengths are shown in Tables S8 and S9. Notably:

- **Extreme variants in VarSim**: VarSim's empirical sampling approach produces extreme-length variants, particularly evident in hg19 where DELs reach 208,848bp (208.8kb) and INSs span up to 83,616bp - orders of magnitude larger than BVSim's maximum lengths (3,993bp and 5,253bp, respectively).

- **Uniform generation in MTS**: MTS shows tightly constrained lengths in hg38 (all variants ≤500bp), with nearly identical quartiles between BVSim and MTS for DUPs and INVs, reflecting their shared uniform length generation for these variant types.

- **Realistic length distributions in BVSim**: For DELs and INSs in hg38, BVSim generates shorter variants (median DEL=115bp vs MTS's 276bp) with left-skewed distributions, better capturing natural size distributions where most variants are shorter but with a long-tail possibility.

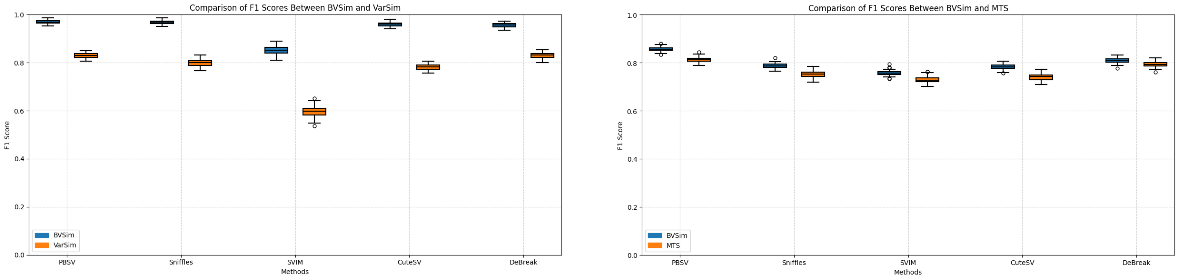

(a) BVSim vs VarSim (hg19) (b) BVSim vs MTS (hg38)

Figure S10: Boxplots showing the original F1 scores for variation detection algorithms on 50 datasets.(a) the F1 scores from BVSim datasets and those from VarSim (hg19). (b) the F1 scores from BVSim datasets and those from MTS (hg38).

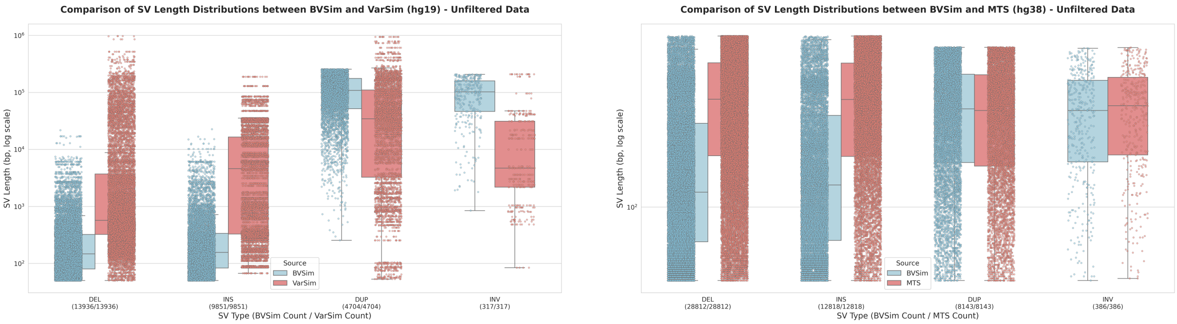

(a) BVSim vs VarSim (hg19) (b) BVSim vs MTS (hg38)

Figure S11: Comparison of simulated SV length distributions on chr21.(a) BVSim vs VarSim (hg19). (b) BVSim vs MTS (hg38).

Table S8: BVSim vs VarSiM: simulated SV Length Statistics (hg19)

Type	Source	Count	Mean	Std Dev	Min	25%	Median	75%	Max
DEL	BVSim	13812	314.37	491.31	50	79	144	318	3993
	VarSim	13798	11817.59	30646.32	50	319	564	3360	208848
INS	BVSim	9752	357.76	597.68	50	82.75	153	331	5253
	VarSim	9768	10550.16	15171.10	67	328	4516	16371	83616
DUP	BVSim	4656	113748.09	71572.90	254	51230.75	107834.5	172834.25	251636
	VarSim	4663	69461.05	90489.05	53	3051	34429	108727	605906
INV	BVSim	317	101964.08	62728.59	843	46065	101719	158071	204322
	VarSim	317	27686.64	51198.18	84	2173	4689	31091	207683

Table S9: BVSim vs MTS: simulated SV Length Statistics (hg38)

Type	Source	Count	Mean	Std Dev	Min	25%	Median	75%	Max
DEL	BVSim	28812	158.21	109.02	50	72	115	220	500
	MTS	28812	275.49	130.60	50	162	276	389	500
INS	BVSim	12818	164.94	112.33	50	73	123	237	499
	MTS	12818	274.57	130.86	50	161	275	388	500
DUP	BVSim	8143	250.90	114.87	50	152	252	349	450
	MTS	8143	248.15	116.44	50	147	248	347.5	450
INV	BVSim	386	242.88	108.68	50	153	248	329.75	446
	MTS	386	251.42	107.86	51	163.25	259.5	339	449

2.5.3 Visualization of SV Distribution Patterns: BVSim vs MTS

In Figure S12(a) and Figure S12(b), the count of insertions and deletions per interval for HG002 is compared to ten random simulations generated by MTS. The results demonstrate that the SVs produced by MTS exhibit a nearly uniform distribution across the intervals.

Conversely, Figure S12(c) through Figure S12(f) illustrate the performance of BVSim, which generates SVs that, while random, align with the distribution patterns observed in benchmark datasets such as HG002 and the 15 *Cell* samples. Specifically, BVSim captures the underlying SV distribution, highlighting its ability to model the complexities of real genomic data effectively. This suggests that BVSim provides a more accurate representation of SV distributions compared to uniform simulation approaches.

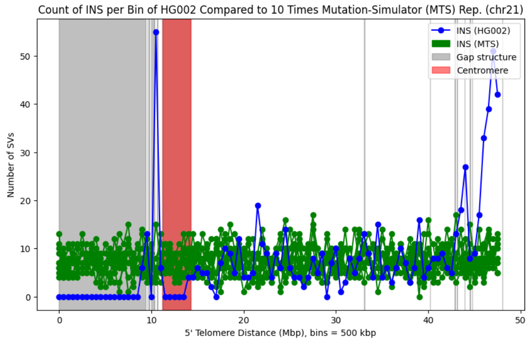

(a) Count of insertions per interval of HG002 compared to 10 times MTS simulation

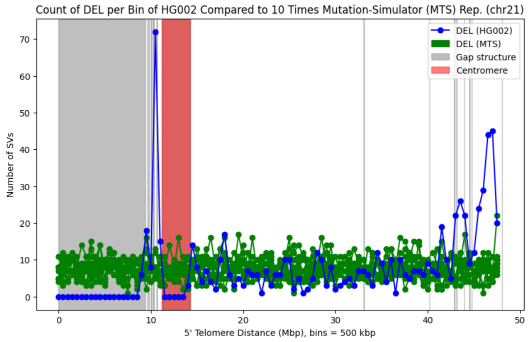

(b) Count of deletions per interval of HG002 compared to 10 times MTS simulation

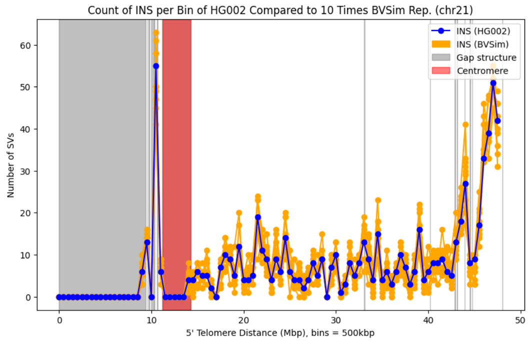

(c) Count of insertions per interval of HG002 compared to 10 times BVSim simulation

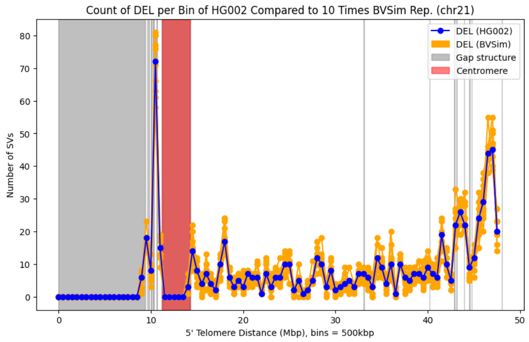

(d) Count of deletions per interval of HG002 compared to 10 times BVSim simulation

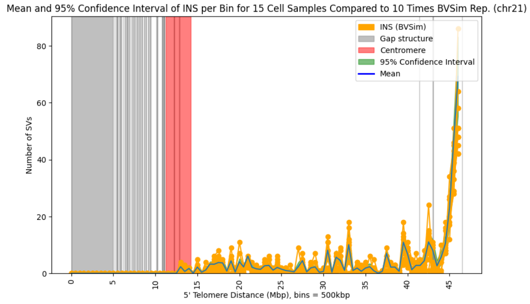

(e) Mean and 95% CI of insertions per interval for 15 *Cell* samples compared to 10 times BVSim simulation

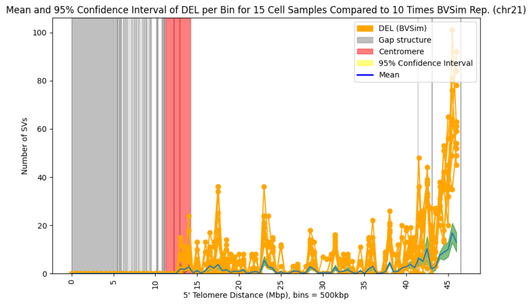

(f) Mean and 95% CI of deletions per interval for 15 *Cell* samples compared to 10 times BVSim simulation

Figure S12: Comparison of simulated SVs generated by MTS and BVSim against benchmark datasets

2.6 Runtime Results Across Different Simulation Modes

All benchmarks were performed on identical hardware with the following specifications in Table S10. However, the minimum requirement to run the code is significantly lower than this configuration - the simulations can run on any system with sufficient storage capacity, even on a single CPU core, though parallel computation requires at least 2 cores.

**Hardware Specification**	**Value**
CPU	2 $\times$ Intel Xeon Gold 6130 (32 threads total)
Base Frequency	2.1 GHz (Turbo Boost up to 3.7 GHz)
Memory	96 GB DDR4 RAM
Storage	Network-attached storage

Table S10: System hardware configuration for all replicates

In Table S11, we show the averaged run times across BVSim's different simulation modes, where all replicates for testing the runtime were performed with 10 replicates to ensure statistical reliability without writing the relative positions. The standard deviations (shown in parentheses) demonstrate the consistency of measurements across repeated runs. Time measurements reflect wall-clock seconds, while memory usage represents peak resident set size. The hardware configuration remained identical across all tests, though natural variations in system state (CPU frequency scaling, memory contention, etc.) contributed to minor runtime differences between non-simultaneous executions.

Table S11: Runtime and footprint results for BVSim modes with 10 replicates

**Mode**	**Sequence**	**Cores**	**Time(s)**	**Max.Memory(MB)**	**SVs**	**SNPs**	**Indels**	**Total**
Exact	20 kbp	1	2.598 ± 0.180	163.812 ± 2.255	40	100	20	145
CSV	20 kbp	1	4.370 ± 0.123	169.149 ± 7.425	115	100	20	235
Uniform	20 kbp	1	2.961 ± 0.300	164.562 ± 1.965	40	100	20	145
Uniform Parallel	chr21(hg19)	32	387.329 ± 4.433	7493.000 ± 1.647	1,479	35,102	7,020	43,601
Wave	chr21(hg19)	32	386.815 ± 3.645	7492.410 ± 1.930	1,479	35,101	7,020	43,600
Wave_region	chr21(hg19)	32	386.135 ± 6.834	7493.790 ± 5.157	1,479	35,101	7,020	43,600

Results based on 10 replicates. Values show mean(SD) with time in seconds and memory in MB.
Memory values converted from KB to MB (divided by 1024).
chr21 simulations used 500k bp bins (48,129,895 bp total).
Total Variations = SVs + SNPs + Indels.
Wave and wave_region modes are with single-sample and empirical mode (SV counts match HG002 bin counts).

In Table S12, we demonstrate the runtime for generating the whole genome under a specific setting.

All benchmarks were conducted on identical hardware configurations. Observed runtime variations between test runs stem from:

- **Dynamic CPU Frequency**: Scaling between 2.1-3.7 GHz
- **Memory Contention**: Background process competition
- **Filesystem Cache**: I/O performance variations

Table S12: Whole-Genome Runtime Performance (hg38 and hg19)

**Chromosome**	**hg38 (15 samples)**		**hg19 (HG002)**	
	Time(s)	Max.Memory(MB)	Time(s)	Max.Memory(MB)
chr1	4180.0	32 267.7	4365.0	32 738.5
chr2	4033.0	31 650.0	4443.0	31 731.6
chr3	2989.0	28 888.1	3010.0	28 942.4
chr4	2802.0	28 407.1	2777.0	28 520.7
chr5	2655.0	27 848.1	2587.0	27 883.9
chr6	2279.0	27 210.4	2393.0	27 266.6
chr7	1947.0	25 268.6	1909.0	25 346.9
chr8	1758.0	24 493.1	1675.0	24 648.2
chr9	1465.0	18 906.7	1483.0	19 670.9
chr10	1458.0	16 700.8	1460.0	18 031.6
chr11	1583.0	23 943.1	1440.0	17 998.3
chr12	1464.0	16 654.9	1639.0	16 767.1
chr13	1087.0	15 744.6	1038.0	15 740.7
chr14	942.0	15 285.1	900.0	15 259.4
chr15	925.0	14 963.9	852.0	15 451.3
chr16	801.0	14 115.3	708.0	13 724.4
chr17	735.0	13 566.1	634.0	12 902.3
chr18	690.0	12 763.6	676.0	12 727.6
chr19	405.0	7927.6	445.0	8025.0
chr20	457.0	8300.0	465.0	8292.3
chr21	274.0	7049.5	251.0	7318.0
chr22	294.0	7490.0	275.0	7458.5
**Total**	29572.0	-	29824.0	-
**Mean**	1344.2	19 847.3	1355.6	20 062.4
**Median**	1458.0	16 654.9	1440.0	17 998.3

Time measured in wall-clock seconds; memory in MB (Max RSS)
hg38: 15-sample batch processing; hg19: single-sample (HG002)
Chromosome order: chr1 (largest) to chr22 (smallest), except chr22 > chr21
Simulation parameters:
SNP rate: 0.0001; small indel rates: 0.00001 each
Region del/ins probabilities: 0.810/0.828
32 CPU cores; 500 kbp bin length
Empirical mode: SV counts match hg38 sample mean/HG002 bin counts

2.7 Dotplot with Annotated SVs

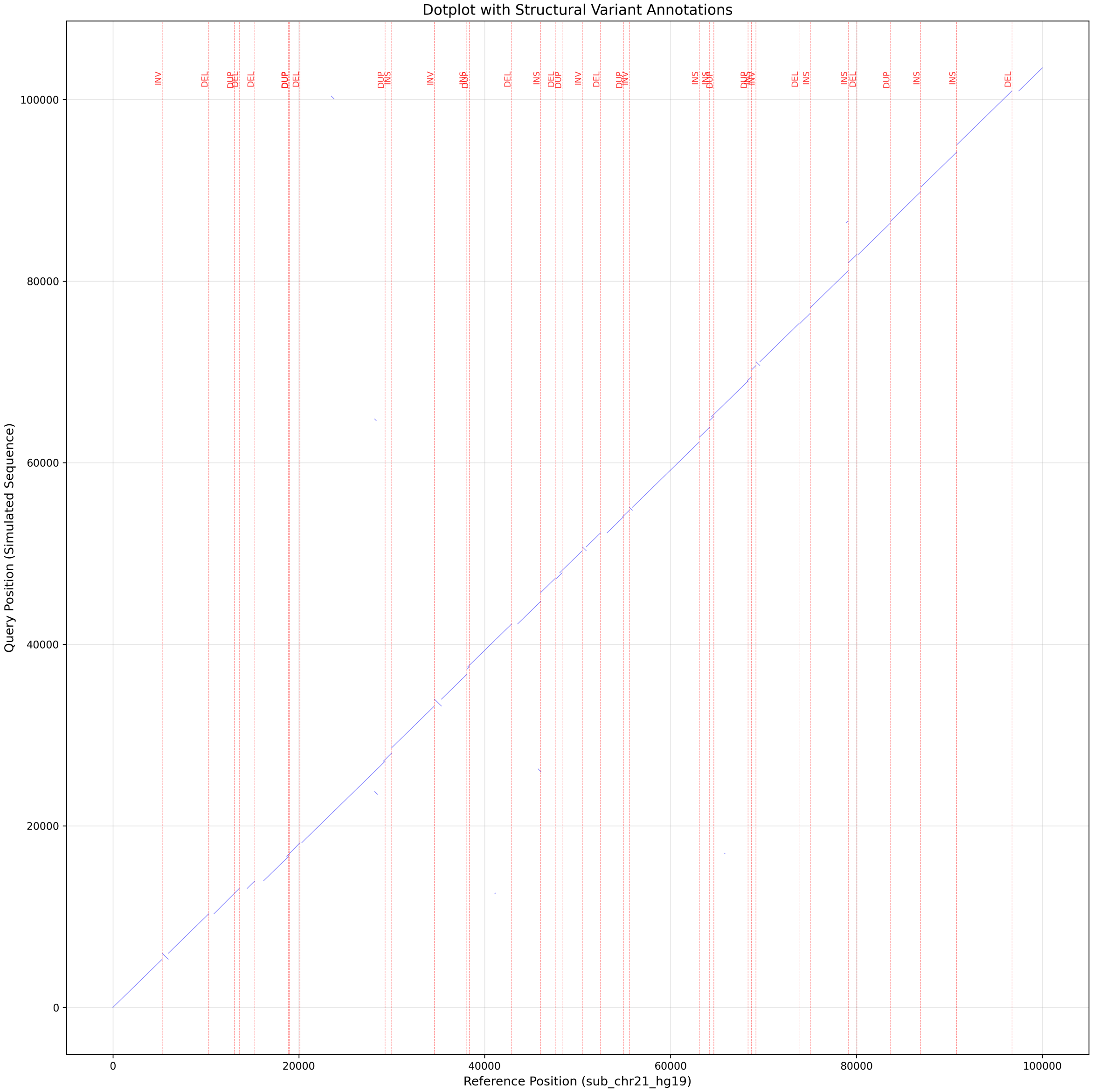

Figure S13: Dotplot comparison between the reference sequence (hg19 chr21:25,000,001-25,100,000) and simulated query sequence, annotated with SVs. Red dashed lines indicate SV positions labeled with their types (DEL/INS/DUP/INV).

Figure S13 illustrates the alignment of the simulated sequence against the reference genome, highlighting the artificially introduced SVs including deletions (DEL), inversions (INV), duplications (DUP), and insertions (INS).

References

Audano, P. A. et al. Characterizing the major structural variant alleles of the human genome. *Cell*, 176(3):663-675, 2019.

Beyter, D. et al. Long-read sequencing of 3,622 Icelanders provides insight into the role of structural variants in human diseases and other traits. *Nature Genetics*, 53(6):779–786, 2021.

Collins, R. L. et al. A structural variation reference for medical and population genetics. *Nature*, 581(7809):444–451, 2020.

Kühl, M. A. et al. Mutation-Simulator: fine-grained simulation of random mutations in any genome. *Bioinformatics*, 37(4):568–569, 2021.

Mu, J. C. et al. VarSim: a high-fidelity simulation and validation framework for high-throughput genome sequencing with cancer applications. *Bioinformatics*, 31(9):1469–1471, 2015.

Tan, J. H. J. et al. A catalogue of structural variation across ancestrally giverse asian genomes. *Nature Communications*, 15(1):9507, 2024.

Zook, J. M. et al. Extensive sequencing of seven human genomes to characterize benchmark reference materials. *Scientific Data*, 3(1):1–26, 2016.
