# Supplementary material for: BVSim: A benchmarking variation simulator mimicking human variation spectrum
Source: Gigascience. 2025 Aug 30;14:giaf095. doi: 10.1093/gigascience/giaf095 (PMC12398280; doi:10.1093/gigascience/giaf095)
Supplement: giaf095_GIGA-D-24-00483_original_submission [file giaf095_giga-d-24-00483_original_submission.pdf]

GigaScience

BVSim: A Benchmarking Variation Simulator Mimicking Human Variation Spectrum

--Manuscript Draft--

Manuscript Number:	GIGA-D-24-00483
Full Title:	BVSim: A Benchmarking Variation Simulator Mimicking Human Variation Spectrum
Article Type:	Technical Note
Funding Information:	Research Grants Council, University Grants Committee (T12-101/23-N, R4012-18 and C7015-23G) — Not applicable<br>Chinese University of Hong Kong (3136017) — Prof Xiaodan Fan<br>Hong Kong University of Science and Technology (3030_009, Z_1056, and BGF.001.2023) — Not applicable<br>Innovation and Technology Commission - Hong Kong (MHP/033/20) — Not applicable
Abstract:	Background: Genomic variations, including single nucleotide polymorphisms, small insertions and deletions, and structural variations, are crucial for understanding evolution and disease. However, comprehensive simulation tools for benchmarking genomic analysis methods are lacking. Existing simulators do not accurately represent the non-uniform distribution and length patterns of SVs in human genomes, and simulating complex structural variations remains challenging.<br>Results: We present BVSim, a flexible tool that provides probabilistic simulations of genomic variations, primarily focusing on human patterns while accommodating diverse species. BVSim effectively simulates both simple and complex structural variations and small variants by mimicking real-life variation distributions, which often exhibit higher frequencies near telomeres and within tandem repeat regions. Notably, BVSim allows users to input single or multiple benchmark samples from any reference genome, enabling the tool to summarize and represent the unique distribution patterns of structural variation positions and lengths specific to those species. Its compatibility with standard file formats facilitates seamless integration into various genomic research workflows, making it a very useful resource for benchmarking downstream tools such as variant callers. With numerical experiments, we show that BVSim generated more realistic sequences significantly different from other simulators' outputs.<br>Conclusions: BVSim is written in Python and freely available to non-commercial users under the GPL3 license. Source code, application guide, and toy examples are provided on the GitHub page at https://github.com/YongyiLuo98/BVSim.
Corresponding Author:	YONGYI LUO, Msc<br>The Chinese University of Hong Kong Faculty of Science<br>Hong Kong, CHINA
Corresponding Author Secondary Information:	
Corresponding Author's Institution:	The Chinese University of Hong Kong Faculty of Science
Corresponding Author's Secondary Institution:	
First Author:	Yongyi Luo, Msc
First Author Secondary Information:	
Order of Authors:	Yongyi Luo, Msc<br>Zhen Zhang, PhD<br>Jiandong Shi, PhD<br>Jingyu Hao

Powered by Editorial Manager® and ProduXion Manager® from Aries Systems Corporation

	Sheng Lian, PhD
	Taobo Hu
	Toyotaka Ishibashi, PhD
	Depeng Wang
	Shu Wang, PhD
	Weichuan Yu, PhD
	Xiaodan Fan, PhD
**Order of Authors Secondary Information:**	
**Additional Information:**	
**Question**	**Response**
Are you submitting this manuscript to a special series or article collection?	No
**Experimental design and statistics**<br><br>Full details of the experimental design and statistical methods used should be given in the Methods section, as detailed in our Minimum Standards Reporting Checklist. Information essential to interpreting the data presented should be made available in the figure legends.<br><br>Have you included all the information requested in your manuscript?	Yes
**Resources**<br><br>A description of all resources used, including antibodies, cell lines, animals and software tools, with enough information to allow them to be uniquely identified, should be included in the Methods section. Authors are strongly encouraged to cite Research Resource Identifiers (RRIDs) for antibodies, model organisms and tools, where possible.<br><br>Have you included the information requested as detailed in our Minimum Standards Reporting Checklist?	Yes
**Availability of data and materials**	Yes

All datasets and code on which the conclusions of the paper rely must be either included in your submission or deposited in publicly available repositories (where available and ethically appropriate), referencing such data using a unique identifier in the references and in the “Availability of Data and Materials” section of your manuscript.<br><br>Have you have met the above requirement as detailed in our Minimum Standards Reporting Checklist?	
GigaScience has policies and guidelines in place for the use of generative AI-writing tools such as ChatGPT. If you have used such writing tools to assist with writing the manuscript this must be declared and cited in the text. Authors should not list AI-writing tools and other AI-assisted technologies as an author or co-author and should acknowledge that they are fully responsible for text generated or refined by AI-writing tools.<p><br><br>A summary of use (particularly in the introduction or among methods) needs to be included at the end of the paper, and the outputs should also be included as a supplementary file hosted in GigaDB or other open repositories. Please read our guidelines for more information. <p><br><br>By submitting to GigaScience, you are aware of the journal's AI-writing tools policy, and if you have declared use of such tools below, you have acknowledged this where appropriate in your manuscript and have made a summary of use and outputs available. <p><br><b>AI-assisted writing tools have been used in the preparation of this manuscript?	Yes

Powered by Editorial Manager® and ProduXion Manager® from Aries Systems Corporation

Click here to access/download;Manuscript;GigaScience_BVSim.pdf

(GIGA)[n] SCIENCE

GigaScience, 2024, 1–5

doi: xx.xxxx/xxxx
Manuscript in Preparation
TECHNICAL NOTE

TECHNICAL NOTE

BVSim: A Benchmarking Variation Simulator Mimicking Human Variation Spectrum

Yongyi Luo[1,†], Zhen Zhang[2,†], Jiandong Shi[2], Jingyu Hao[2], Sheng Lian[2], Taobo Hu[3], Toyotaka Ishibashi[4], Depeng Wang[5], Shu Wang[3,*], Weichuan Yu[2,*] and Xiaodan Fan[1,*]

[1]Department of Statistics, The Chinese University of Hong Kong, Hong Kong SAR, China and [2]Department of Electronic and Computer Engineering, The Hong Kong University of Science and Technology, Hong Kong SAR, China and [3]Department of Breast Surgery, Peking University People's Hospital, Beijing, China and [4]Division of Life Science, Hong Kong University of Science and Technology, Hong Kong SAR, China and [5]GrandOmics Inc, Beijing, China

[*]eeyu@ust.hk; shuwang@pkuph.edu.cn; xfan@cuhk.edu.hk
[†]Contributed equally.

Abstract

Background: Genomic variations, including single nucleotide polymorphisms, small insertions and deletions, and structural variations, are crucial for understanding evolution and disease. However, comprehensive simulation tools for benchmarking genomic analysis methods are lacking. Existing simulators do not accurately represent the non-uniform distribution and length patterns of SVs in human genomes, and simulating complex structural variations remains challenging.
Results: We present BVSim, a flexible tool that provides probabilistic simulations of genomic variations, primarily focusing on human patterns while accommodating diverse species. BVSim effectively simulates both simple and complex structural variations and small variants by mimicking real-life variation distributions, which often exhibit higher frequencies near telomeres and within tandem repeat regions. Notably, BVSim allows users to input single or multiple benchmark samples from any reference genome, enabling the tool to summarize and represent the unique distribution patterns of structural variation positions and lengths specific to those species. Its compatibility with standard file formats facilitates seamless integration into various genomic research workflows, making it a very useful resource for benchmarking downstream tools such as variant callers. With numerical experiments, we show that BVSim generated more realistic sequences significantly different from other simulators' outputs.
Conclusions: BVSim is written in Python and freely available to non-commercial users under the GPL3 license. Source code, application guide, and toy examples are provided on the GitHub page at https://github.com/YongyiLuo98/BVSim.

Key words: genomic variations; sequence simulation; benchmarking

Background

Genomic variations, including single nucleotide polymorphisms (SNPs), small insertions and deletions (indels) under 50 base pairs (bps), and structural variations (SVs), are of vital importance due to their close relation with evolution, disease, and so on [1]. There is a huge amount of research about SNPs and small indels with the development of the next-generation (short-read) sequencing technologies [2, 3, 4]. However, due to the short read length limitation, SVs (usually defined as longer than 50 bps) cannot be detected accurately. Thus third-generation (long-read) sequencing technologies have emerged in the last decade, greatly facilitating the understanding of SVs [5]. Importantly, several studies have reported that SV positions in the human genome may not follow a simple uniform

Compiled on: October 29, 2024.
Draft manuscript prepared by the author.

distribution. More specifically, SVs tend to occur at higher rates near telomeres and within tandem repeat (TR) regions [6, 7]. Additionally, the length distributions of these SVs exhibit non-uniform patterns. A large-scale study of Icelandic individuals [7] revealed prominent peaks in the SV length distribution at approximately 300 bps, 2,500 bps, and 6,000 bps. Further analysis of publicly available SV data from comprehensive characterizations using 15 representative samples [6] and the benchmark dataset HG002 [8, 9] also demonstrated similar non-uniform patterns for both SV locations and lengths (Supplementary Fig. S1-S4, Table S1 and S2).

To carry out further studies on SVs as well as SNPs and small indels, it is important to build a realistic and comprehensive simulator of these variations to benchmark the related methods and tools, such as alignment, variation calling, and consensus inference. Existing genome simulators, while able to simulate SVs as well as SNPs and small indels, have limitations in accurately representing SV features. VarSim [10] was among the first tools with SV simulation function. It is primarily tailored for human cancer genome simulation and samples SV positions from fixed regions, limiting its ability to simulate diverse SV distributions. Simulome [11] introduced random variations with different options, but it was primarily designed for prokaryotic genomes. simuG [12] can simulate some SVs more randomly as compared with VarSim, but its parameter tuning capabilities are limited to adjusting overall characteristics, such as the proportion of insertions and deletions, and it can only generate variations uniformly, rather than allowing direct manipulation of specific variation probabilities or counts. VISOR [13] allowed users to manually input SV positions, but it is overly complicated to simulate a large number of SVs. Mutation-Simulator [14] cannot accommodate non-uniform deletions or SV length distributions.

Furthermore, complex SVs (CSVs) have been identified in the human genome, particularly in individuals with autism spectrum disorder and other developmental abnormalities [15]. CSVs exhibit more complex genomic rearrangements beyond simple insertions, deletions, duplications, and inversions. VISOR, used in the SVision study [16], serves as a CSV simulator that employs a multi-step curation process to generate simulated data for evaluating the detection of CSVs. In the simulation of CSVs with VISOR, not only the position is fixed but also the steps are complicated, limiting both the types and generality of CSVs, which highlights the need for more comprehensive and automated CSV simulation capabilities.

To address these limitations, we present BVSim, a command-line tool that automatically simulates non-uniform and haplotype-resolved variations with randomness. The patterns of simulated variations are derived from benchmark datasets of the human genome, mimicking the human variation spectrum. This tool highlights the importance of simulating a wide range of SVs, from simple to complex, as well as micro variants.

Importantly, BVSim allows users to input browser extensible data (BED) files containing empirical variations derived from benchmark datasets of various species or specific human subpopulations. By summarizing SV patterns from these user-supplied datasets, BVSim generates vectors representing the local SV probabilities, supporting realistic simulations of variations across diverse reference genomes and enhancing its utility for researchers studying a wide range of organisms. Furthermore, BVSim enables users to specify parameters for simulating various scenarios for benchmarking purposes, including configurations that control the rate, length, and distribution patterns of genomic variations.

Findings

BVSim, a command-line package written in Python, is designed to randomly simulate realistic and comprehensive variations and create human pseudo-genomes sequences by default, with options to learn from input empirical variations of other species. It enables the integration of simple SVs, including deletions, insertions, inversions, tandem duplications, as well as intrachromosomal translocations, both balanced and unbalanced. In addition, it offers parameters to control rates or numbers of micro-variations, including small indels and SNPs. Furthermore, it can emulate 18 types of CSVs illustrated in Fig. 1(a) including [15, 16]:

- ID1: Tandem Inverted Duplication (TanInvDup)
- ID2: Dispersed Inverted Duplication (DisInvDup)
- ID3: Dispersed Duplication (DisDup)
- ID4: Inversion with 5’ or 3’ Flanking Deletion (DEL+INV/INV+DEL)
- ID5: 5’ Deletion and Dispersed Inverted Duplication (DEL+DisInvDup)
- ID6: 5’ Deletion and Dispersed Duplication (DEL+DisDup)
- ID7: Tandem Duplication and 3’ Deletion (TanDup+DEL)
- ID8: Tandem Inverted Duplication and 3’ Deletion (TanInvDup+DEL)
- ID9: Tandem Duplication, Deletion and Inversion (TanDup+DEL+INV)
- ID10: Tandem Inverted Duplication, Deletion and Inversion (TanInvDup+DEL+INV)
- ID11: Paired-Deletion Inversion (DEL+INV+DEL)
- ID12: Inversion with 5’ Flanking Duplication (DUP+INV)
- ID13: Inversion with 3’ Flanking Duplication (INV+DUP)
- ID14: Paired-Duplication Inversion (DUP+INV+DUP)
- ID15: Inversion with 5’ Flanking Duplication and 3’ Flanking Deletion (DUP+INV+DEL)
- ID16: Inversion with 5’ Flanking Deletion and 3’ Flanking Duplication (DEL+INV+DUP)
- ID17: Inverted Duplication with Flanking Triplication (DupTripDup-INV)
- ID18: Insertion with Deletion (INSdel)

Human reference genome

For the human genome, we derive the length distributions of SVs from HG002 [8] and the 15 samples published in *Cell* [6]. For SNPs, we embed a learned substitution transition matrix from the dbSNP database [17]. With a user-specified bin size, BVSim learns the distribution of SV positions per interval. It can model the SVs per interval as a multinomial distribution parameterized by the observed frequencies in HG002 (GRCh37/hg19 as reference) or sample the SV numbers per interval from a Gaussian distribution with the mean and standard deviation computed across the 15 samples (GRCh38/hg38 as reference). Calling ‘-hg19’ or ‘-hg38’ and specifying the chromosome name can activate the above procedures automatically for the human genome.

General usage

BVSim supports both uniform and non-uniform SV generation. Users can input any reference genome sequence, and BVSim can summarize the SV position distribution from user-provided BED files. Besides, BVSim provides five distinct modes for genomic sequence simulation: uniform mode, uniform-parallel mode, CSV mode, wave mode, and wave-region mode. Each mode supports blocking gap structures or user-defined regions in the sequence, preventing any variation in those blocked areas.

Uniform mode and uniform-parallel mode

The uniform mode introduces variations uniformly across the sequence. This mode is recommended when no additional information is available about the species or when the simulation scale is small. For long references or when simulating numerous variations, the uniform-parallel mode is recommended. This mode distributes the uniform variation simulation across multiple processes, signif-

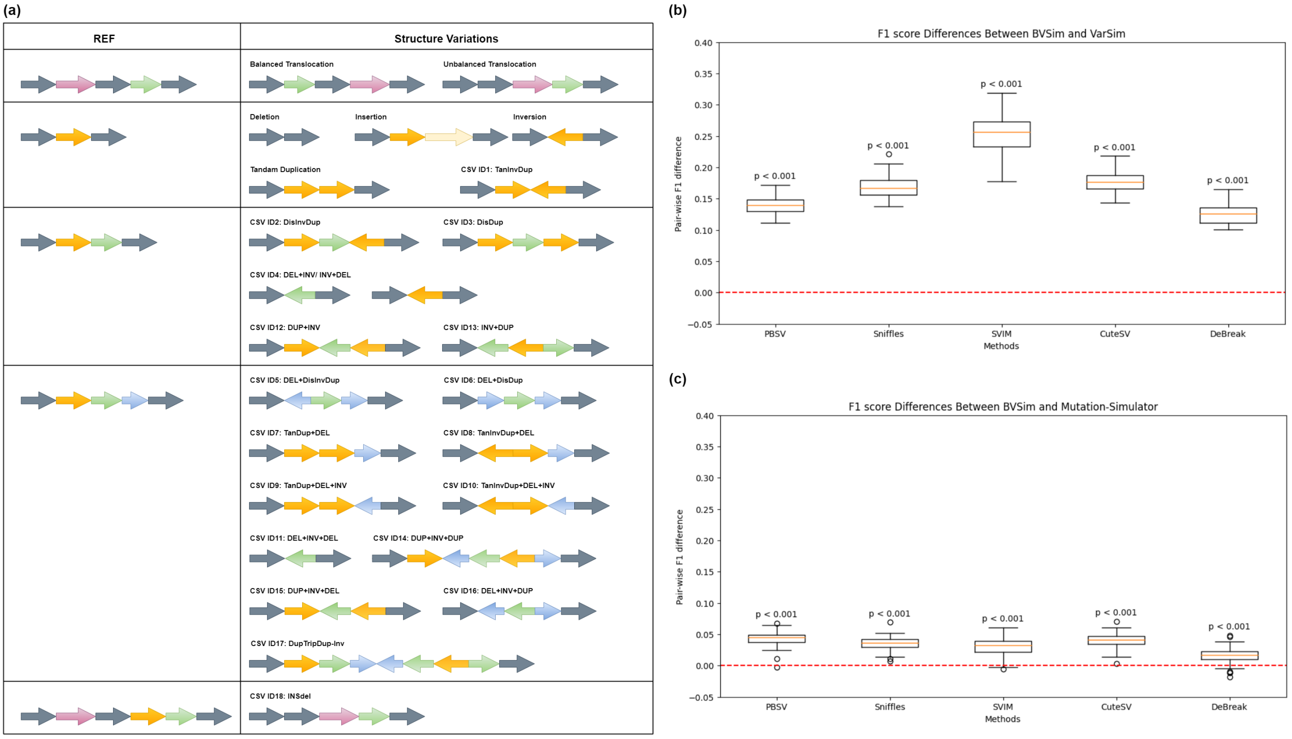

Figure 1. (a) Definitions of the SV types supported by BVSim. (b) A boxplot showing the pairwise differences in F1 scores for variation detection algorithms on 50 datasets, calculated as the F1 scores from BVSim datasets minus those from VarSim. (c) A boxplot illustrating the pairwise differences in F1 scores for variation detection algorithms on 50 datasets, calculated as the F1 scores from BVSim datasets minus those from Mutation-Simulator.

icantly accelerating the simulation process.

CSV mode

In CSV mode, BVSim generates 18 CSV types with user-defined quantities or distributed based on empirical rates. Users can customize CSV length distributions and introduce or avoid other variations as needed.

Wave mode and wave-region mode

The wave mode utilizes a BED file to model SV distributions based on location and length, generating non-uniform profiles that reflect learned distributions. Notably, if multiple benchmark datasets are available, the wave mode allows for the expansion of the population. For instance, it can simulate the genomes of patients with breast cancer.

The wave-region mode further enhances this capability by enabling customized SV probabilities for specific genomic regions, such as TR regions, allowing for varied SV densities compared to the overall genomic sequence. Both modes enhance simulation efficiency by parallelizing variant generation through sequence segmentation.

Output format

BVSim outputs the simulated pseudo-genomic sequence in a standard FASTA format, compatible with reads generators like PBSIM2 [18]. It also generates a table detailing variation positions, aiding in distinguishing true variations from sequencing errors. For introduced mutations in the variant call format (VCF) file, BVSim includes CSV-TYPE and CSV-INDEX fields in the INFO column, outlining CSV types and components.
Fig. 2 illustrates the workflow of BVSim, encapsulated within a dashed box, and demonstrates how the output files interact with read simulators, the alignment tool Minimap2[19], Samtools[20], and evaluation tools such as Truvari[21].

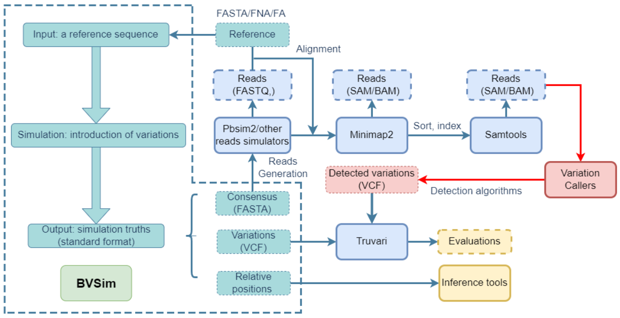

Figure 2. Workflow of BVSim and interactions with downstream analysis tools.

Comparison with existing simulators

Existing SV simulators can be broadly categorized into two main approaches. One approach involves sampling SV positions from empirical datasets, such as VarSim and simuG (inversions and translocations), which restricts the probability space to a finite number of fixed positions and specific lengths (Supplementary Fig. S5(a)). This confinement limits the randomness introduced during benchmarking and may affect the representativeness of simulated variations. The other approach attempts to generate SVs in a more general manner, as exemplified by the Mutation-Simulator. It tries to address the limitations of empirical sampling by modeling SV rates using a uniform rate (Supplementary Fig. S5(b)) or a learned rate per segment. However, these flexible simulation approaches may still be overly simplified, as they do not include non-uniform deletions or assume a uniform length distribution for all simulated variations (Supplementary Fig. S5(c)). Our BVSim aims to provide a more comprehensive and representative simulation of SV landscapes. It samples SV positions and lengths by interpolating both the empirical SV positions and length distributions (Supplementary Fig. S5(d)), resulting in a diverse and realistic sampling

distribution that captures the complex patterns observed in real SV data, thereby enhancing the fidelity and representativeness of the simulated variations.

To evaluate the impact of variation simulators on the performance of variant callers, we conducted two numerical comparisons. The first experiment involved a comparison between BVSim and VarSim, utilizing the hg19 reference genome, with a focus on sampling from fixed regions. The second experiment compared BVSim with Mutation-Simulator, employing the hg38 reference genome to contrast our approach with a random but uniform simulator. Each experiment generated an average of 1,000 SVs across 50 sequence pairs (chromosome 21), ensuring that the number of each variant type and their respective length ranges were consistent across all methodologies. The results, illustrated in Fig. 1(b) and Fig. 1(c), demonstrate significant pairwise differences in the F1 scores of five variation detection algorithms when assessed on datasets generated by BVSim, VarSim, and Mutation-Simulator. The five variation detection algorithms evaluated were PBSV (version 2.9.0)[22], Sniffles [23], SVIM [24], CuteSV [25] and DeBreak [26]. Notably, the pairwise differences were significantly greater than zero, indicating that sequences produced by BVSim and previous simulators are significantly different. Thus, we shall choose a simulator that produces more realistic sequences, which is exactly what BVSim is designed for.
In support of this, we provide a visualization of ten realizations of the simulations for benchmark datasets in Fig. S6, where BVSim aligns with real genomic complexities, in contrast to the uniform distribution produced by the Mutation-Simulator.

Discussion

BVSim is a Python-based tool that offers a comprehensive solution for simulating realistic genomic variations, accommodating SNPs, small indels as well as simple and complex SVs. It provides flexibility in the distribution of variations, supports parallel processing for efficiency, and allows for user-defined parameters, making it a versatile tool for evaluating variation calling tools and facilitating genomic research.

Nevertheless, BVSim can be further improved. Firstly, it can currently only simulate one genomic sequence at a time. In case needed, a modified version of BVSim to generate multiple conditionally independent and identically distributed sequences will be more efficient to run BVSim multiple times independently. Secondly, BVSim may be extended to simulate the genomic sequences of a whole family. Thirdly, the speed of BVSim may be improved by coding in C/C++ with a careful parallel computing scheme.

Availability of Source Code and Requirements

- Project name: BVSim
- Project home page: `https://github.com/YongyiLuo98/BVSim`
- Operating system(s): Linux
- Programming language: Python
- Other requirements: Conda package manager
- License: GPL3

Additional Files

Supplementary Figure S1. SV distribution across chromosomes (HG002) for chromosomes 1 to 22.
Supplementary Figure S2. Mean SV counts and 95% confidence intervals across chromosomes (15 Cell Samples) for chromosomes 1 to 22.
Supplementary Figure S3. Distribution of insertions and deletions in tandem repeat and non-tandem repeat regions in 15 Cell samples and HG002 for chromosomes 1 to 22.
Supplementary Figure S4. The empirical length distribution of HG002 and 15 Cell samples.
Supplementary Figure S5. The illustration of sampling probability spaces for different simulators using chromosome 10 as an example.
Supplementary Figure S6. Comparison of simulated SVs generated by Mutation-Simulator and BVSim against benchmark datasets.
Supplementary Table S1. Counts and proportions of deletions in tandem repeat regions across 15 Cell samples.
Supplementary Table S2. Counts and proportions of insertions in tandem repeat regions across 15 Cell samples.

Data availability

The GRCh37 (hg19) and GRCh38 (hg38) human reference genomes can be found in the NCBI Assembly database [27] with accession numbers GCA_000001405.1 and GCA_000001405.29, respectively. The dbSNP dataset is accessible at dbSNP data. Variant calling results for HG002 are available at HG002_NA24385_son.

Additionally, details about the 15 *Cell* samples can be found in Table S1 of the accompanying paper [6]. The tandem repeat regions are published by NCBI and can be retrieved from the following files: hg19.simpleRepeat.bed.gz for GRCh37 and hg38.repeats.bed.gz for GRCh38.

Declarations

List of abbreviations

BED: browser extensible data; bps: base pairs; CSVs: complex structural variations; indels: insertions and deletions; SNPs: single nucleotide polymorphisms; SVs: structural variations; TR: tandem repeat; VCF: variant call format.

Competing Interests

The authors declare that they have no competing interests.

Funding

This work was supported by the internal grants 3030_009, Z_1056, and BGF.001.2023 from HKUST, 3136017 from CUHK, T12-101/23-N (RGC), R4012-18 (RGC), C7015-23G (RGC), and MHP/033/20 (ITC) from the Hong Kong SAR Government of China.

Author's Contributions

Y.L. and Z.Z.: software coding and design, manuscript writing; J.S., T.I., and S.L.: literature review; J.H.: numerical experiments; T.H., D.W.: data organization; S.W., W.Y., and X.F.: project conception, proposal writing, and funding application; J.S., X.F.: manuscript drafting, review, and editing.

References

1. Stange M, Barrett RD, Hendry AP. The importance of genomic variation for biodiversity, ecosystems and people. Nat Rev Genet 2021;22(2):89–105. https://doi.org/10.1038/s41576-020-00288-7.
2. Jarvie T. Next generation sequencing technologies. Drug Discov Today Technol 2005;2(3):255–60. https://doi.org/10.1016/j.ddtec.2005.08.003.
3. Mullaney JM, Mills RE, Pittard WS, et al. Small insertions

and deletions (INDELs) in human genomes. Hum Mol Genet 2010;19(R2):R131–6. https://doi.org/10.1093/hmg/ddq400.
4. Mielczarek M, Szyda J. Review of alignment and SNP calling algorithms for next-generation sequencing data. J Appl Genet 2016;57(1):71–9. https://doi.org/10.1007/s13353-015-0292-7.
5. Schadt EE, Turner S, Kasarskis A. A window into third-generation sequencing. Hum Mol Genet 2010;19(R2):R227–40. https://doi.org/10.1093/hmg/ddq416.
6. Audano PA, Sulovari A, Graves-Lindsay, et al. Characterizing the major structural variant alleles of the human genome. Cell 2019;176(3):663–75. https://doi.org/10.1016/j.cell.2018.12.019.
7. Beyter D, Ingimundardottir H, Oddsson A, et al. Long-read sequencing of 3,622 Icelanders provides insight into the role of structural variants in human diseases and other traits. Nat Genet 2021;53(6):779–86. https://doi.org/10.1038/s41588-021-00865-4.
8. Zook JM, Catoe D, McDaniel J, et al. Extensive sequencing of seven human genomes to characterize benchmark reference materials. Sci Data 2016;3(1):1–26. https://doi.org/10.1038/sdata.2016.25.
9. Zook JM, Hansen NF, Olson ND, et al. A robust benchmark for detection of germline large deletions and insertions. Nat Biotechnol 2020;38(11):1347–55. https://doi.org/10.1038/s41587-020-0538-8.
10. Mu JC, Mohiyuddin M, Li J, et al. VarSim: a high-fidelity simulation and validation framework for high-throughput genome sequencing with cancer applications. Bioinformatics 2015;31(9):1469–71. https://doi.org/10.1093/bioinformatics/btu828.
11. Price A, Gibas C. Simulome: a genome sequence and variant simulator. Bioinformatics 2017;33(12):1876–8. https://doi.org/10.1093/bioinformatics/btx091.
12. Yue JX, Liti G. simuG: a general-purpose genome simulator. Bioinformatics 2019;35(21):4442–4. https://doi.org/10.1093/bioinformatics/btz424.
13. Bolognini D, Sanders A, Korbel JO, et al. VISOR: a versatile haplotype-aware structural variant simulator for short-and long-read sequencing. Bioinformatics 2020;36(4):1267–9. https://doi.org/10.1093/bioinformatics/btz719.
14. Kühl M, Stich B, Ries D. Mutation-Simulator: fine-grained simulation of random mutations in any genome. Bioinformatics 2021;37(4):568–9. https://doi.org/10.1093/bioinformatics/btaa716.
15. Collins RL, Brand H, Redin CE, et al. Defining the diverse spectrum of inversions, complex structural variation, and chromothripsis in the morbid human genome. Genome Biol 2017;18:1–21. https://doi.org/10.1186/s13059-017-1158-6.
16. Lin J, Wang S, Audano PA, et al. SVision: a deep learning approach to resolve complex structural variants. Nat Methods 2022;19(10):1230–3. https://doi.org/10.1038/s41592-022-01609-w.
17. Sherry ST, Ward MH, Kholodov M, et al. dbSNP: the NCBI database of genetic variation. Nucleic Acids Res 2001;29(1):308–11. https://doi.org/10.1093/nar/29.1.308.
18. Ono Y, Asai K, Hamada M. PBSIM2: a simulator for long-read sequencers with a novel generative model of quality scores. Bioinformatics 2021;37(5):589–95. https://doi.org/10.1093/bioinformatics/btaa835.
19. Li H. Minimap2: pairwise alignment for nucleotide sequences. Bioinformatics 2018;34(18):3094–3100. https://doi.org/10.1093/bioinformatics/bty191.
20. Danecek P, Bonfield JK, Liddle J, et al. Twelve years of SAMtools and BCFtools. Gigascience 2021;10(2):giab008. https://doi.org/10.1093/gigascience/giab008.
21. English AC, Menon VK, Gibbs RA, et al. Truvari: refined structural variant comparison preserves allelic diversity. Genome Biol 2022;23(1):271. https://doi.org/10.1186/s13059-022-02840-6.
22. PBSV, PacBio structural variant (SV) calling and analysis tools (version 2.9.0); 2024. https://github.com/PacificBiosciences/pbsv/releases/tag/v2.9.0, accessed 26 Oct 2024.
23. Sedlazeck FJ, Rescheneder P, Smolka M, et al. Accurate detection of complex structural variations using single-molecule sequencing. Nat Methods 2018;15(6):461–8. https://doi.org/10.1038/s41592-018-0001-7.
24. Heller D, Vingron M. SVIM: structural variant identification using mapped long reads. Bioinformatics 2019;35(17):2907–15. https://doi.org/10.1093/bioinformatics/btz041.
25. Jiang T, Liu Y, Jiang Y, et al. Long-read-based human genomic structural variation detection with cuteSV. Genome Biol 2020;21:1–24. https://doi.org/10.1186/s13059-020-02107-y.
26. Chen Y, Wang AY, Barkley CA, et al. Deciphering the exact breakpoints of structural variations using long sequencing reads with DeBreak. Nat Commun 2023;14(1):283. https://doi.org/10.1038/s41467-023-35996-1.
27. National Center for Biotechnology Information (NCBI). Assembly database; 2024. https://www.ncbi.nlm.nih.gov/assembly, accessed: 2024-10-26.

Click here to access/download
Supplementary Material
BVSim_supplement_file.pdf
