# Supplementary material for: BVSim: A benchmarking variation simulator mimicking human variation spectrum
Source: Gigascience. 2025 Aug 30;14:giaf095. doi: 10.1093/gigascience/giaf095 (PMC12398280; doi:10.1093/gigascience/giaf095)
Supplement: giaf095_GIGA-D-24-00483_Revision_1 [file giaf095_giga-d-24-00483_revision_1.pdf]

GigaScience

BVSim: A Benchmarking Variation Simulator Mimicking Human Variation Spectrum

--Manuscript Draft--

**Manuscript Number:**	GIGA-D-24-00483R1
**Full Title:**	BVSim: A Benchmarking Variation Simulator Mimicking Human Variation Spectrum
**Article Type:**	Technical Note
**Funding Information:**	Chinese University of Hong Kong (3136017) — Prof Xiaodan Fan<br>Hong Kong University of Science and Technology (3030_009, Z_1056, and BGF.001.2023) — Not applicable<br>Innovation and Technology Commission - Hong Kong (MHP/033/20) — Not applicable<br>Research Grants Council, University Grants Committee (T12-101/23-N, R4012-18 and C7015-23G) — Not applicable

Funder	Grant recipient
Chinese University of Hong Kong (3136017)	Prof Xiaodan Fan
Hong Kong University of Science and Technology (3030_009, Z_1056, and BGF.001.2023)	Not applicable
Innovation and Technology Commission - Hong Kong (MHP/033/20)	Not applicable
Research Grants Council, University Grants Committee (T12-101/23-N, R4012-18 and C7015-23G)	Not applicable

Abstract:

Background: Genomic variations, including single nucleotide polymorphisms, small insertions and deletions, and structural variations, are crucial for understanding evolution and disease. However, comprehensive simulation tools for benchmarking genomic analysis methods are lacking. Existing simulators do not accurately represent the non-uniform distribution and length patterns of SVs in human genomes, and simulating complex structural variations remains challenging.
Results: We present BVSim, a flexible tool that provides probabilistic simulations of genomic variations, primarily focusing on human patterns while accommodating diverse species. BVSim effectively simulates both simple and complex structural variations and small variants by mimicking real-life variation distributions, which often exhibit higher frequencies near telomeres and within tandem repeat regions. Notably, BVSim allows users to input single or multiple benchmark samples from any reference genome, enabling the tool to summarize and represent the unique distribution patterns of structural variation positions and lengths specific to those species. Its compatibility with standard file formats facilitates seamless integration into various genomic research workflows, making it a very useful resource for benchmarking downstream tools such as variant callers. With numerical experiments, we show that BVSim generated more realistic sequences significantly different from other simulators' outputs.
Conclusions: BVSim is written in Python and freely available to non-commercial users under the GPL3 license. Source code, application guide, and toy examples are provided on the GitHub page at https://github.com/YongyiLuo98/BVSim. The tool is registered in SciCrunch (RRID:SCR_026926), bio.tools (biotools:BVSim), and WorkflowHub (doi:10.48546/WORKFLOWHUB.WORKFLOW.1361.1).

**Corresponding Author:**	YONGYI LUO, Msc<br>The Chinese University of Hong Kong Faculty of Science<br>Hong Kong, CHINA
**Corresponding Author Secondary Information:**	
**Corresponding Author's Institution:**	The Chinese University of Hong Kong Faculty of Science
**Corresponding Author's Secondary Institution:**	
**First Author:**	Yongyi Luo, Msc
**First Author Secondary Information:**	
**Order of Authors:**	Yongyi Luo, Msc<br>Zhen Zhang, PhD

Powered by Editorial Manager® and ProduXion Manager® from Aries Systems Corporation

	Jiandong Shi, PhD
	Jingyu Hao
	Sheng Lian, PhD
	Taobo Hu
	Toyotaka Ishibashi, PhD
	Depeng Wang
	Shu Wang, PhD
	Weichuan Yu, PhD
	Xiaodan Fan, PhD
**Order of Authors Secondary Information:**	
**Response to Reviewers:**	The responses are uploaded as a separated pdf file since some figures and tables are included.
**Additional Information:**	
**Question**	**Response**
Are you submitting this manuscript to a special series or article collection?	No
**Experimental design and statistics**<br><br>Full details of the experimental design and statistical methods used should be given in the Methods section, as detailed in our Minimum Standards Reporting Checklist. Information essential to interpreting the data presented should be made available in the figure legends.<br><br>Have you included all the information requested in your manuscript?	Yes
**Resources**<br><br>A description of all resources used, including antibodies, cell lines, animals and software tools, with enough information to allow them to be uniquely identified, should be included in the Methods section. Authors are strongly encouraged to cite Research Resource Identifiers (RRIDs) for antibodies, model organisms and tools, where possible.<br><br>Have you included the information requested as detailed in our Minimum	Yes

Standards Reporting Checklist?	
**Availability of data and materials**<br><br>All datasets and code on which the conclusions of the paper rely must be either included in your submission or deposited in publicly available repositories (where available and ethically appropriate), referencing such data using a unique identifier in the references and in the “Availability of Data and Materials” section of your manuscript.<br><br>Have you have met the above requirement as detailed in our Minimum Standards Reporting Checklist?	Yes
GigaScience has policies and guidelines in place for the use of generative AI-writing tools such as ChatGPT. If you have used such writing tools to assist with writing the manuscript this must be declared and cited in the text. Authors should not list AI-writing tools and other AI-assisted technologies as an author or co-author and should acknowledge that they are fully responsible for text generated or refined by AI-writing tools.<p><br><br>A summary of use (particularly in the introduction or among methods) needs to be included at the end of the paper, and the outputs should also be included as a supplementary file hosted in GigaDB or other open repositories. Please read our guidelines for more information. <p><br><br>By submitting to GigaScience, you are aware of the journal's AI-writing tools policy, and if you have declared use of such tools below, you have acknowledged this where appropriate in your manuscript and have made a summary of use and outputs available. <p><br><b>AI-assisted writing tools have been used in the preparation of this manuscript?	Yes

Revised manuscript with changes highlighted
Click here to access/download;Manuscript;GigaScience_BVSim_revised.pdf

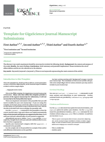

GigaScience, 2025, 1–8

doi: xx.xxxx/xxxx
Manuscript in Preparation
TECHNICAL NOTE

TECHNICAL NOTE

BVSim: A Benchmarking Variation Simulator Mimicking Human Variation Spectrum

Yongyi Luo[1,†], Zhen Zhang[2,†], Jiandong Shi[2], Jingyu Hao[2], Sheng Lian[2], Taobo Hu[3], Toyotaka Ishibashi[4], Depeng Wang[5], Shu Wang[3,*], Weichuan Yu[2,*] and Xiaodan Fan[1,*]

[1]Department of Statistics, The Chinese University of Hong Kong, Hong Kong SAR, China and [2]Department of Electronic and Computer Engineering, The Hong Kong University of Science and Technology, Hong Kong SAR, China and [3]Department of Breast Surgery, Peking University People's Hospital, Beijing, China and [4]Division of Life Science, Hong Kong University of Science and Technology, Hong Kong SAR, China and [5]GrandOmics Inc, Beijing, China

[*]eeyu@ust.hk; shuwang@pkuph.edu.cn; xfan@cuhk.edu.hk
[†]Contributed equally.

Abstract

Background: Genomic variations, including single nucleotide polymorphisms, small insertions and deletions, and structural variations, are crucial for understanding evolution and disease. However, comprehensive simulation tools for benchmarking genomic analysis methods are lacking. Existing simulators do not accurately represent the non-uniform distribution and length patterns of SVs in human genomes. And simulating complex structural variations remains challenging.
Results: We present BVSim, a flexible tool that provides probabilistic simulations of genomic variations, primarily focusing on human patterns while accommodating diverse species. BVSim effectively simulates both simple and complex structural variations and small variants by mimicking real-life variation distributions, which often exhibit higher frequencies near telomeres and within tandem repeat regions. Notably, BVSim allows users to input single or multiple benchmark samples from any reference genome, enabling the tool to summarize and represent the unique distribution patterns of structural variation positions and lengths specific to those species. Its compatibility with standard file formats facilitates seamless integration into various genomic research workflows, making it a very useful resource for benchmarking downstream tools such as variant callers. With numerical experiments, we show that BVSim generated more realistic sequences significantly different from other simulators' outputs.
Conclusions: BVSim is written in Python and freely available to non-commercial users under the GPL3 license. Source code, application guide, and toy examples are provided on the GitHub page at https://github.com/YongyiLuo98/BVSim. The tool is registered in SciCrunch (RRID:SCR_026926), bio.tools (biotools:BVSim), and WorkflowHub (doi:10.48546/WORKFLOWHUB.WORKFLOW.1361.1).

Key words: genomic variations; sequence simulation; benchmarking

Background

Genomic variations, including single nucleotide polymorphisms (SNPs), small insertions and deletions (indels) under 50 base pairs (bps), and structural variations (SVs), are of vital importance due to their close relation with evolution, disease, and so on [1]. There is a huge amount of research about SNPs and small indels with the development of the next-generation (short-read) sequencing technologies [2, 3, 4]. However, due to the short read length limitation, SVs (usually defined as longer than 50 bps) cannot be detected accu-

Compiled on: May 17, 2025.
Draft manuscript prepared by the author.

rately. Thus third-generation (long-read) sequencing technologies have emerged in the last decade, greatly facilitating the understanding of SVs [5]. Importantly, several studies have reported that SV positions in the human genome may not follow a simple uniform distribution. More specifically, SVs tend to occur at higher rates near telomeres and within tandem repeat (TR) regions [6, 7]. Additionally, the length distributions of these SVs exhibit non-uniform patterns. A large-scale study of Icelandic individuals [7] revealed prominent peaks in the SV length distribution at approximately 300 bps, 2,500 bps, and 6,000 bps. Further analysis of publicly available SV data from comprehensive characterizations using 15 representative samples [6], the benchmark dataset HG002 called by 19 SV callers from the Genome in a Bottle (GIAB) study [8, 9] as well as the Human Genome Structural Variation Consortium (HGSVC) samples [10] demonstrated similar non-uniform patterns for both SV locations and lengths (Supplementary Figures S1-S6, Tables S1-S4).

To carry out further studies on SVs as well as SNPs and small indels, it is important to build a realistic and comprehensive simulator of these variations to benchmark the related methods and tools, such as alignment, variation calling, and consensus inference. Existing genome simulators, while able to simulate SVs as well as SNPs and small indels, have limitations in accurately representing SV features. VarSim [11] was among the first tools with SV simulation function. It is primarily tailored for human cancer genome simulation and samples SV positions from fixed regions, limiting its ability to simulate diverse SV distributions. Simulome [12] introduced random variations with different options, but it was primarily designed for prokaryotic genomes. simuG [13] can simulate some SVs more randomly as compared with VarSim, but its parameter tuning capabilities are limited to adjusting overall characteristics, such as the proportion of insertions and deletions, and it can only generate variations uniformly, rather than allowing direct manipulation of specific variation probabilities or counts. SURVIVOR [14] simulates variants only randomly with limited fine-tuning parameters, although it can also evaluate the SV callers. RSVSim [15] applied a parametric model for the SV length distribution, limiting its flexibility to reflect more complex patterns in real data. Although RSVSim simulates breakpoints near some overlapped points from the empirical datasets for the human genome, it relies on pre-processed repeat annotations from hg19 [16] and cannot incorporate newer empirical SV datasets or adapt to custom genomic annotations. The package enforces fixed mechanistic biases through hardcoded repeat associations [17][18][19][20], whereas modern approaches require dynamic learning from diverse input data. While Sim-it [21] can simulate customized SVs and long reads, its utility is limited by both the inability to model small indels and SNPs concurrently and by an opaque telomeric bias mechanism lacking adjustable parameters for comparative studies. VISOR [22] allowed users to manually input SV positions, but it is overly complicated to simulate a large number of SVs. Mutation-Simulator (MTS) [23] simulates SVs and small variants with more fine-tuning options, but cannot accommodate non-uniform deletions or SV length distributions.

Furthermore, complex SVs (CSVs) have been identified in the human genome, particularly in individuals with autism spectrum disorder and other developmental abnormalities [24]. CSVs exhibit more complex genomic rearrangements beyond simple insertions, deletions, tandem duplications, and inversions. VISOR, used in the SVision study [25], serves as a CSV simulator that employs a multi-step curation process to generate simulated data for evaluating the detection of CSVs. In the simulation of CSVs with VISOR, not only the position is fixed but also the steps are complicated, limiting both the types and generality of CSVs, which highlights the need for more comprehensive and automated CSV simulation capabilities.

To address these limitations, we present BVSim, a command-line tool that automatically simulates non-uniform and haplotype-resolved variations with randomness. The patterns of simulated variations are derived from benchmark datasets of the human genome, mimicking the human variation spectrum. This tool highlights the importance of simulating a wide range of SVs, from simple to complex, as well as small variants.

Importantly, BVSim allows users to input browser extensible data (BED) files containing empirical variations derived from benchmark datasets of various species or specific human subpopulations. By summarizing SV patterns from these user-supplied datasets, BVSim generates vectors representing the local SV probabilities, supporting realistic simulations of variations across diverse reference genomes and enhancing its utility for researchers studying a wide range of organisms. Furthermore, BVSim enables users to specify parameters for simulating various scenarios for benchmarking purposes, including configurations that control the rate, length, and distribution patterns of genomic variations.

Findings

BVSim, a command-line package written in Python, is designed to randomly simulate realistic and comprehensive variations and create human-like genomic sequences by default, with options to learn from input empirical variations of other species. It enables the integration of simple SVs, including deletions, insertions, inversions, tandem duplications, as well as intrachromosomal translocations, both balanced and unbalanced. In addition, it offers parameters to control rates or numbers of small variations, including small indels and SNPs. Furthermore, it can emulate 18 types of CSVs illustrated in Figure 1(a)[24, 25].

Methodology

BVSim offers eight operational modes designed to accommodate diverse simulation requirements. In the default mimic mode, the tool simulates realistic variations by mimicking the variations on the human reference genomes hg19 or hg38. At the same time, it maintains the flexibility to accept any reference genome sequence as input. Six other genomic sequence simulation modes (wave mode, wave-region mode, CSV mode, uniform mode, uniform-parallel mode, and exact mode) are provided for diverse application scenarios. The remaining VCF mode provides a pre-processing function when users would like to simulate population-specific empirical SVs.

The tool first learns empirical distributions from the observed data in a non-parametric way, then generates different types of variants sequentially following the order: translocation, inversion, tandem duplications, CSV ID1-ID18, deletions, insertions, small deletions, small insertions, SNPs. Later variants will avoid overlapping with previous ones. The sampling method may vary for different variants and different sequence simulation modes.

Distribution learning from empirical data

For each variant, if the observation data amount allows, BVSim extracts the variants' empirical length distribution by counting and spatial distribution along chromosomes by adjustable binning. These empirical distributions will be later used to synthesize such variant by random sampling from them.

Specifically, for a variant type t, its length distribution is computed as:

$$P_t(l) = \frac{n_{t,l}}{\sum_{l'} n_{t,l'}}, \tag{1}$$

where $n_{t,l}$ is the number of observed occurrences of length-l variants for type t. The denominator normalizes the distribution with the totals across N individual input samples.

To learn the spatial distribution of a variant type, the genome

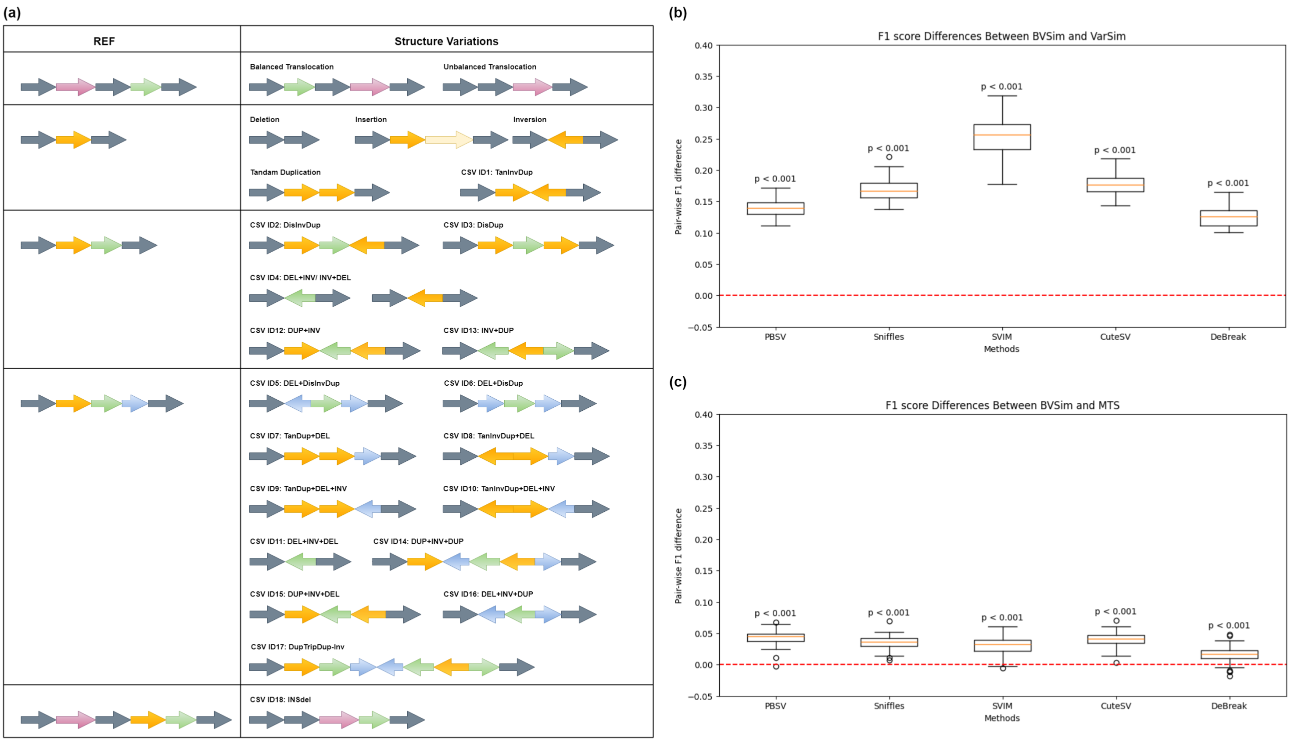

Figure 1. (a) Definitions of the SV types supported by BVSim. (b) A boxplot showing the pairwise differences in F1 scores for variation detection algorithms on 50 datasets, calculated as the F1 scores from BVSim datasets minus those from VarSim. (c) A boxplot illustrating the pairwise differences in F1 scores for variation detection algorithms on 50 datasets, calculated as the F1 scores from BVSim datasets minus those from MTS.

is partitioned into M bins $\{S_1, ..., S_M\}$ of size L (customizable with default value=500kbp), with the terminal bin accommodating residual bases:

$$S_j = \begin{cases} [(j-1)L, jL) & \text{for } j = 1, ..., M-1 \\ [(j-1)L, G] & \text{for } j = M \end{cases}, \tag{2}$$

where G is the sequence length and M is the integer part of G/L.

For the case of inputting a single empirical sample (e.g., HG002), we observe a count c_j of a same variant type in the bin S_j. For the case of inputting multiple samples (e.g., the Cell dataset or HGSVC dataset), we observe $c_{j,i}$ for the jth bin of the ith sample, where $i = 1, \cdots, N$ and $j = 1, \cdots, M$. Then we calculate a mean μ_j and a standard deviation value σ_j^2 from these $c_{j,i}$'s for the jth bin.

General sampling methods

Different variant types are generated one after the other. For a specific variant type, we first decide the number of variants within each bin, then determine their location within the corresponding bin.

Let v_{total} be the total number of a specific variant type to be simulated. It is either directly specified by the user (e.g., '-sv_del 100' sets the total of deletions as 100), or determined from the input observed sample(s) if the sum-preserving feature is activated by the '-sum' flag (i.e., v_{total} is either equal to $\sum_{j=1}^{M} c_j$ for single-sample case and $\sum_{j=1}^{M} \mu_j$ for multi-sample case).

The simulated count k_j for the bin S_j ($j = 1, \ldots, M$) satisfies $\sum_{j=1}^{M} k_j = v_{\text{total}}$. If the user chooses the fixed option to preserve observed distributions, we set k_j as c_j for the single-sample case and the integer part of μ_j for the multi-sample case. If the user chooses the random option, k_j's will be sampled from the corresponding distribution learned from the empirical data. More specifically, for the multi-sample case, we sample c_j from a non-negative discrete normal distribution with mean μ_j and variance σ_j^2; for the single-sample case, c_j is observed. Then for both cases, we sample $(k_1, ..., k_M) \sim \text{Multinomial}(v_{\text{total}}, (p_1, ..., p_M))$, where $p_j = c_j / \sum_k c_k$.

The start position of each variant within its corresponding bin is sampled uniformly from all available positions within the bin. For the mimic mode and the wave-region mode, we allow users to further specify different variant showing-up probabilities for TR and non-TR regions. A visualization of the wave-region mode is given in Figure S8.

The length of a small indel (1-5 bp) is independently sampled from an empirical distribution calculated from the Database of Genomic Variants (DGV) [26]. More specifically, the probability of a small indel's length equal to 1, 2, 3, 4, 5 is equal to $\frac{6}{8}, \frac{1}{8}, \frac{1}{16}, \frac{1}{32}, \frac{1}{32}$, respectively.

To simulate a translocation, BVSim samples two regions (A and B). A translocation is either balanced (A and B are exchanged) or unbalanced (A is lost and B is inserted into A's start point) in the sequence. To simulate a duplication, one copied region and the inserted position need to be sampled. To simulate an inversion/insertion/deletion, BVSim samples a start point and a length. To simulate a SNP, BVSim samples a location and a replacement base from a learned substitution transition matrix from the dbSNP database [27]. We also ensure that the SVs and small variants will not be simulated from the regions related to other variants. Complex SVs combine these simple variants with spatial proximity. All SVs and CSVs can be generated together with small indels and SNPs.

Default mode - mimic human

BVSim's default mode generates variants by mimicking the real human variant spectrum. Specifically, we derive the empirical distributions of variants from individual-level benchmark HG002 (GRCh37/hg19 as reference) [8], the 15 samples published in *Cell* [6], and the 32 *HGSVC* samples (both GRCh38/hg38 as reference)

[10]. Calling '-mimic' with '-hg19' or '-hg38' and specifying the chromosome name can activate the procedures to mimic the above built-in empirical distributions. For the hg38 reference, BVSim combines both hg38 datasets (*Cell* and *HGSVC*) by default. Note that their overlapping sample (NA12878) is automatically deduplicated during processing. Users can restrict empirical distribution learning to a specific dataset by specifying either '-cell' or '-hgsvc' flag with '-hg38' flag.

Wave mode and wave-region mode

The wave mode triggered by the '-wave' flag utilizes BED file(s) to model variant distributions based on location and length, generating non-uniform profiles that reflect learned distributions. Notably, if multiple benchmark datasets are available, the wave mode allows for the expansion of the population. For instance, it can simulate the genomes of patients with breast cancer.

The wave-region mode triggered by the '-wave_region' flag further enhances this capability by enabling customized variant probabilities for specific genomic regions, such as TR regions, allowing for varied variant densities compared to the overall genomic sequence. Both modes enhance simulation efficiency by parallelizing variant generation through sequence segmentation.

CSV mode

The CSV mode triggered by the '-csv' flag can generate the 18 CSV types and other SVs defined in Figure 1(a), together with small variants following user-defined quantities or rates. Users can customize CSV length distributions and introduce or avoid other variations.

Uniform mode and uniform-parallel mode

The uniform mode is triggered by the '-uniform' flag, while the uniform-parallel mode is triggered by adding the '-cores' parameter. Both modes distribute variants uniformly across the input sequence while respecting non-overlapping constraints and blocked regions. Uniform mode is recommended when no additional information is available about the species or when the simulation scale is small. For long references or when simulating numerous variations, the uniform-parallel mode is recommended. This mode distributes the uniform variation simulation across multiple processes, significantly accelerating the simulation process.

Exact mode

Calling '-exact' will activate the exact mode which enables precise simulation of variants at user-specified genomic positions through a structured table format. The input requires three key pieces of information for each variant: genomic location, variant type, and length, with additional fields required for specific variant classes. Balanced translocations must include coordinates for both exchanged regions, while tandem duplications require the source and destination positions. Complex variants can be constructed by specifying multiple simple variants that collectively form the composite event.

VCF mode

The VCF mode serves as a preprocessing step for population-level variant filtering prior to simulation. Activating this mode requires calling '-vcf' and specifying a VCF file path after '-vcf_file'. BVSim builds this mode to receive population-level SVs files such as gnomAD v4 [28]. Users can input selection criteria to obtain a table

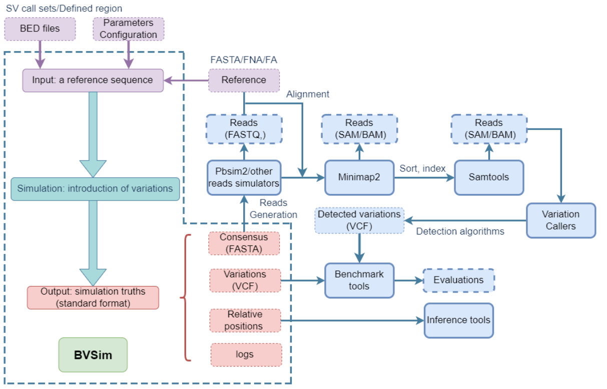

Figure 2. Workflow of BVSim's sequence simulation and interactions with downstream analysis tools. Files in purple boxes are user-provided inputs, while those in red boxes are outputs. Blue boxes show downstream tools.

recording population-specific variants (e.g. "AF_fin >0.001" for Finnish allele frequency larger than 0.001). See Supplementary Tables S5–S7 for a summary of population codes and annotation rules of the gnomAD VCF file. The resulting SV table can be input into the exact mode for sequence generation after excluding overlapping SVs.

Input validation

To ensure data integrity, BVSim automatically validates all input tables against format requirements before simulation. This validation process checks for proper field completion, coordinate validity, and logical consistency between variant types and their required parameters. Example tables demonstrating the correct format are provided in the GitHub repository, showing both basic and complex variant configurations.

Output format for sequence simulation modes

BVSim outputs the simulated pseudo-genomic sequence in a standard FASTA format, compatible with reads generators like PBSIM2 [29]. It also generates a table detailing variation positions, aiding in distinguishing true variations from sequencing errors. For introduced mutations in the variant call format (VCF) file (version 4.3), BVSim includes CSV-TYPE and CSV-INDEX fields in the INFO column, outlining CSV types and components.

Figure 2 illustrates the workflow of BVSim, encapsulated within a dashed box, and demonstrates how the output files interact with read simulators, the alignment tool Minimap2[30], SAMtools and BCFtools [31], and benchmark tools such as Truvari [32], SURVIVOR [14], vcfdist [33].

Extension to polyploid and non-human genomes

BVSim's framework supports simulations beyond diploid human genomes through flexible parameterization. For haploid references (e.g., microbial or viral genomes), users may directly simulate reads from any input sequence without modification. Polyploid systems (e.g., triploid or tetraploid organisms) can be modeled by processing homologous chromosomes sequentially: users may supply distinct variant profiles for each chromosome set and aggregate the results. The tool also accommodates somatic heterogeneity through user-defined variant tables with specified allele frequencies.

Comparison with existing simulators

The primary goal is to develop an automated realistic variant simulator with controlled randomness to generate diverse synthesis data. Existing genome simulators demonstrate the following primary constraints. First, CSV simulation remains particularly underdeveloped, with current solutions like VISOR requiring laborious manual curation processes. Except for lacking a CSV simulation function, many existing variant simulators either only simulate SVs (e.g., Sim-it) or only simulate small variants, instead of trying to cover all types of genome variants. For those with both SVs and small variant simulation capability, empirical tools such as VarSim and RSVSim sample variants only from predetermined genomic regions. Lastly, parametric tools including Simulome, simuG, SURVIVOR, and MTS rely on oversimplified uniform distributions. BVSim overcomes these constraints through its innovative distribution learning framework, yielding a non-uniform sampling space as shown in Supplementary Figure S9(d), which maintains biological fidelity while permitting users' adjustments.

To illustrate the influence of different simulation mechanisms on downstream analyses, BVSim was compared against two representative simulation paradigms: the purely empirical approach VarSim and the purely random approach MTS. The selection of MTS as the parametric representative was based on its relative flexibility compared to Simulome, simuG and SURVIVOR, which share the same fundamental uniform sampling methodology but offer fewer user-configurable parameters. Several tools were excluded from quantitative comparison due to fundamental incompatibilities with automated benchmarking requirements. Sim-it's inability to model small variants renders it unsuitable for comprehensive genomic simulation. VISOR's manual curation requirements and RSVSim's outdated annotation dependencies similarly preclude meaningful performance evaluation against BVSim's automated framework.

We designed two benchmarking experiments to evaluate the effect of these methodological differences. The first experiment compared BVSim against VarSim using the hg19 reference genome, focusing on fixed region sampling. The second experiment contrasted BVSim with MTS using the hg38 reference genome to examine differences between realistic and uniform simulation approaches. Both experiments generated consistent variant sets comprising 1,000 simple SVs distributed across 50 chromosome 21 sequence pairs, with carefully matched variant type distributions and length ranges.

For downstream effect evaluation, we employed five established SV detection tools: PBSV (version 2.9.0) [34], Sniffles (version 2.2) [35], SVIM (version 2.0.0) [36], CuteSV (version 2.0.3) [37], and DeBreak (version 1.0.2) [38]. The 'truvari bench' command from Truvari (version 4.1.0) [32] was used to benchmark the detection results and calculate F1 scores.

As shown in Supplementary Figure S11 and Table S8-S9, VarSim and MTS exhibit systematic biases in their length distributions: VarSim produces right-skewed distributions with extreme outliers, while MTS generates uniformly distributed variants within fixed size ranges. In contrast, BVSim maintains naturally distributed variant lengths that agree better with biological observations. This systematic bias of VarSim and MTS may mislead performance evaluation for variant callers. This claim is supported by the observed F1 score differences summarized in Figure 1b-c, which are significantly different from zero. The original F1 scores are shown in Supplementary Figure S10.

Our controlled experiments reveal two key advantages of BVSim's approach:

- **Precision in length distribution**: While maintaining the same potential length ranges as other simulators, BVSim naturally generates shorter SVs with left-skewed distributions that better match empirical length distributions as presented in Supplementary Figure S6.
- **Flexible control**: The framework has a more realistic default setting, but it can also provide simulations according to users' requests.

These results collectively validate that BVSim's probabilistic sampling framework produces more realistic variant profiles than existing simulation approaches, while maintaining greater flexibility in parameter control. This property is important for correct downstream method evaluation.

The comparison of downstream results shows that the above distribution difference between VarSim/MTS simulation and the real distribution will bias downstream conclusions. It is to say that the unrealistically distributed variants generated from VarSim or MTS will lead to misleading variant caller performance, because the variant caller's performance on the simulated data from VarSim or MTS will be different from its performance on the real human genome, which is successfully mimicked by our BVSim. Thus, we shall choose a simulator that produces more realistic sequences, which is exactly what BVSim is designed for. In support of this, we provide a visualization of ten realizations of the simulations for benchmark datasets in Supplementary Figure S12, where BVSim aligns with real genomic complexities, in contrast to the uniform distribution produced by the MTS.

Discussion

BVSim is a Python-based tool that offers a comprehensive solution for simulating realistic genomic variations, accommodating SNPs, small indels as well as simple and complex SVs. It provides flexibility in the distribution of variations, supports parallel processing for efficiency, and allows user-defined parameters, making it a versatile tool for evaluating variation calling tools and facilitating genomic research. The performance characteristics of the tool, including runtime metrics on different genomic scales, are detailed in Supplementary Table S10-S12.

The tool's reference genome compatibility extends beyond standard human assemblies to include the complete telomere-to-telomere (T2T) CHM13 genome [39]. While the benchmarking SV calling datasets for T2T CHM13 remain under development, BVSim enables forward-looking research by supporting both random and empirically modeled variation generation on this complete genomic template.

While offering these capabilities, BVSim does have several limitations that guide future development priorities. The current single-sequence processing architecture, while enabling efficient parallel computation and polyploid simulation, prevents modeling of interchromosomal events like translocations. The tool's variant representation is also constrained by the completeness of input benchmark sets, where reliance on resources like GIAB v0.6 [9] may perpetuate biases against complex or repeat-associated variants. Additionally, while BVSim can model various ploidy levels through manual configuration, its utility for population-scale studies would benefit from specialized optimization.

These limitations directly inform BVSim's development roadmap. We plan to enhance computational efficiency through optimized C/C++ implementation while expanding biological scope to include family-based simulations and automated species-specific parameterization. Particular attention will be given to agriculturally important polyploids and the integration of emerging community resources like the testHG008curation. By improving variant spectrum coverage through more comprehensive benchmark sets, future versions will address current gaps in SV representation while maintaining the tool's modularity and cross-species applicability.

Availability of Source Code and Requirements

- Project name: BVSim version 1.0.0
- Project home page: `https://github.com/YongyiLuo98/BVSim`
- License: GPL-3.0
- SciCrunch RRID: SCR_026926
- bio.tools ID: BVSim
- WorkflowHub DOI: `https://doi.org/10.48546/WORKFLOWHUB.WORKFLOW.1361.1`
- Configuration templates:
  - Default parameters: bvsim_config.yaml
  - User-configurable parameters: custom_config.yaml
  - Pre-configured scenarios: example shell scripts

System Requirements:

- Operating system: Linux
- Programming language: Python 3.6 or higher
- Package management: Conda (environment.yml provided)
- Hardware Requirements:
  - Minimum 4GB RAM (8GB+ recommended for large datasets)
  - Multi-core CPU recommended for parallel processing
  - Adequate disk space for reference genomes and output files

Additional Files

Supplementary Figure S1. SV distribution across chromosomes (HG002) for chromosomes 1 to 22.
Supplementary Figure S2. Mean SV counts and 95% confidence intervals across chromosomes (15 Cell Samples) for chromosomes 1 to 22.
Supplementary Figure S3. Mean SV counts and 95% confidence intervals across chromosomes (32 HGSVC Samples) for chromosomes 1 to 22.
Supplementary Figure S4. Distribution of insertions and deletions in tandem repeat and non-tandem repeat regions in 15 Cell samples and HG002 for chromosomes 1 to 22.
Supplementary Figure S5. Distribution of insertions and deletions in tandem repeat and non-tandem repeat regions in 32 HGSVC samples for chromosome 1 to 22.
Supplementary Figure S6. The empirical length distribution of HG002, 15 Cell samples and 32 HGSVC samples.
Supplementary Figure S7. Comparison of deletion distribution generation methods on chromosome 21. The fixed option (a,c) directly uses observed counts from input samples, while the random option (b,d) redistributes variants according to bin-specific probabilities. Single-sample versus multi-sample comparisons demonstrate method consistency across datasets.
Supplementary Figure S8. Simulated SV distributions in wave-region mode showing TR enrichment (chr21/hg38). Deletions and insertions demonstrate increased density in user-defined tandem repeat regions with 500kbp bin size.
Supplementary Figure S9. The illustration of sampling probability spaces for different simulators using chromosome 10 as an example.
Supplementary Figure S10. Boxplots showing the original F1 scores for variation detection algorithms on 50 datasets.(a) the F1 scores from BVSim datasets and those from VarSim (hg19). (b) the F1 scores from BVSim datasets and those from MTS (hg38).
Supplementary Figure S11. Comparison of simulated SV length distributions on chr21. (a) BVSim vs VarSim (hg19). (b) BVSim vs MTS (hg38).
Supplementary Figure S12. Comparison of simulated SVs generated by MTS and BVSim against benchmark datasets.
Supplementary Figure S13. Dotplot comparison between the reference sequence (hg19 chr21:25,000,001-25,100,000) and simulated query sequence, annotated with SVs. Red dashed lines indicate SV positions labeled with their types (DEL/INS/DUP/INV).
Supplementary Table S1. Counts and proportions of deletions in tandem repeat regions across 15 Cell samples.
Supplementary Table S2. Counts and proportions of insertions in tandem repeat regions across 15 Cell samples.
Supplementary Table S3. Counts and proportions of deletions in tandem repeat regions across 32 HGSVC samples (transposed).
Supplementary Table S4. Counts and proportions of insertions in tandem repeat regions across 32 HGSVC samples (transposed).
Supplementary Table S5. Population codes and descriptions in gnomAD SV v4.1.
Supplementary Table S6. Key population-level SV annotation fields in gnomAD v4.1.
Supplementary Table S7. Genotype frequency metrics in gnomAD SV v4.1.
Supplementary Table S8. BVSim vs VarSiM: simulated SV Length Statistics (hg19).
Supplementary Table S9. BVSim vs MTS: simulated SV Length Statistics (hg38).
Supplementary Table S10. System hardware configuration for all replicates.
Supplementary Table S11. Runtime and footprint results for BVSim modes with 10 replicates.
Supplementary Table S12. Whole-Genome Runtime Performance (hg38 and hg19).

Data Availability

The GRCh37 (hg19) and GRCh38 (hg38) human reference genomes can be found in the NCBI Assembly database [40] with accession numbers GCA_000001405.1 and GCA_000001405.29, respectively. The T2T-CHM13 v2.0 genome is also available at NCBI (GCA_009914755.4) [39].

The dbSNP dataset is accessible at dbSNP data. The DGV dataset is available at DGV homepage. Variant calling results for HG002 are available at HG002_NA24385_son. The HGSVC dataset is available at HGSVC2 v1.0. The official documentation and download access of gnomAD are at gnomAD v4.

Additionally, details about the 15 *Cell* samples can be found in Table S1 of the accompanying paper [6]. The tandem repeat regions are published by NCBI and can be retrieved from the following files: hg19.simpleRepeat.bed.gz for GRCh37 and hg38.repeats.bed.gz for GRCh38.

The community reported SVs and CSVs can be found at its GitHub homepage: testHG008curation.

Declarations

List of abbreviations

BED: browser extensible data; bps: base pairs; CSVs: complex structural variations; GIAB: genome in a bottle; HGSVC: human genome structural variation consortium; DGV: database of genomic variants; indels: insertions and deletions; MTS: Mutation-Simulator; SNPs: single nucleotide polymorphisms; SVs: structural variations; TR: tandem repeat; T2T: telomere-to-telomere; VCF: variant call format.

Competing Interests

The authors declare that they have no competing interests.

Funding

This work was supported by the internal grants 3030_009, Z_1056, and BGF.001.2023 from HKUST, 3136017 from CUHK, T12-101/23-N (RGC), R4012-18 (RGC), C7015-23G (RGC), and MHP/033/20 (ITC) from the Hong Kong SAR Government of China.

Author's Contributions

Y.L. and Z.Z.: software coding and design, manuscript writing; J.S., T.I., and S.L.: literature review; J.H.: numerical experiments; T.H., D.W.: data organization; S.W., W.Y., and X.F.: project conception, proposal writing, and funding application; J.S., X.F.: manuscript drafting, review, and editing.

References

1. Stange M, Barrett RD, Hendry AP. The importance of genomic variation for biodiversity, ecosystems and people. Nat Rev Genet 2021;22(2):89–105. https://doi.org/10.1038/s41576-020-00288-7.
2. Jarvie T. Next generation sequencing technologies. Drug Discov Today Technol 2005;2(3):255–60. https://doi.org/10.1016/j.ddtec.2005.08.003.
3. Mullaney JM, Mills RE, Pittard WS, et al. Small insertions and deletions (INDELs) in human genomes. Hum Mol Genet 2010;19(R2):R131–6. https://doi.org/10.1093/hmg/ddq400.
4. Mielczarek M, Szyda J. Review of alignment and SNP calling algorithms for next-generation sequencing data. J Appl Genet 2016;57(1):71–9. https://doi.org/10.1007/s13353-015-0292-7.
5. Schadt EE, Turner S, Kasarskis A. A window into third-generation sequencing. Hum Mol Genet 2010;19(R2):R227–40. https://doi.org/10.1093/hmg/ddq416.
6. Audano PA, Sulovari A, Graves-Lindsay, et al. Characterizing the major structural variant alleles of the human genome. Cell 2019;176(3):663–75. https://doi.org/10.1016/j.cell.2018.12.019.
7. Beyter D, Ingimundardottir H, Oddsson A, et al. Long-read sequencing of 3,622 Icelanders provides insight into the role of structural variants in human diseases and other traits. Nat Genet 2021;53(6):779–86. https://doi.org/10.1038/s41588-021-00865-4.
8. Zook JM, Catoe D, McDaniel J, et al. Extensive sequencing of seven human genomes to characterize benchmark reference materials. Sci Data 2016;3(1):1–26. https://doi.org/10.1038/sdata.2016.25.
9. Zook JM, Hansen NF, Olson ND, et al. A robust benchmark for detection of germline large deletions and insertions. Nat Biotechnol 2020;38(11):1347–55. https://doi.org/10.1038/s41587-020-0538-8.
10. Tan JHJ, Li Z, Porta MG, et al. A catalogue of structural variation across ancestrally giverse asian genomes. Nat Commun 2024;15(1):9507. https://doi.org/10.1038/s41467-024-53620-8.
11. Mu JC, Mohiyuddin M, Li J, et al. VarSim: a high-fidelity simulation and validation framework for high-throughput genome sequencing with cancer applications. Bioinformatics 2015;31(9):1469–71. https://doi.org/10.1093/bioinformatics/btu828.
12. Price A, Gibas C. Simulome: a genome sequence and variant simulator. Bioinformatics 2017;33(12):1876–8. https://doi.org/10.1093/bioinformatics/btx091.
13. Yue JX, Liti G. simuG: a general-purpose genome simulator. Bioinformatics 2019;35(21):4442–4. https://doi.org/10.1093/bioinformatics/btz424.
14. Jeffares DC, Jolly C, Hoti M, et al. Transient structural variations have strong effects on quantitative traits and reproductive isolation in fission yeast. Nat Commun 2017;8(1):14061. https://doi.org/10.1038/ncomms14061.
15. Bartenhagen C, Dugas M. RSVSim: an R/Bioconductor package for the simulation of structural variations. Bioinformatics 2013;29(13):1679–1681. https://doi.org/10.1093/bioinformatics/btt198.
16. Meyer LR, Zweig AS, Hinrichs AS, et al. The UCSC Genome Browser database: extensions and updates 2013. Nucleic Acids Res 2012;41(D1):D64–D69. https://doi.org/10.1093/nar/gks1048.
17. Chen W, Kalscheuer V, Tzschach A, et al. Mapping translocation breakpoints by next-generation sequencing. Genome Res 2008;18(7):1143–1149. http://www.genome.org/cgi/doi/10.1101/gr.076166.108.
18. Mills RE, Walter K, Stewart C, et al. Mapping copy number variation by population-scale genome sequencing. Nature 2011;470(7332):59–65. https://doi.org/10.1038/nature09708.
19. Ou Z, Stankiewicz P, Xia Z, et al. Observation and prediction of recurrent human translocations mediated by NAHR between nonhomologous chromosomes. Genome Res 2011;21(1):33–46. http://www.genome.org/cgi/doi/10.1101/gr.111609.110.
20. Pang AWC, Migita O, MacDonald JR, et al. Mechanisms of formation of structural variation in a fully sequenced human genome. Hum Mutat 2013;34(2):345–354. https://doi.org/10.1002/humu.22240.
21. Dierckxsens N, Li T, Vermeesch JR, Xie Z. A benchmark of structural variation detection by long reads through a realistic simulated model. Genome Biol 2021;22:1–16. https://doi.org/10.1186/s13059-021-02551-4.
22. Bolognini D, Sanders A, Korbel JO, et al. VISOR: a versatile haplotype-aware structural variant simulator for short-and long-read sequencing. Bioinformatics 2020;36(4):1267–9. https://doi.org/10.1093/bioinformatics/btz719.
23. Kühl M, Stich B, Ries D. Mutation-Simulator: fine-grained simulation of random mutations in any genome. Bioinformatics 2021;37(4):568–9. https://doi.org/10.1093/bioinformatics/btaa716.
24. Collins RL, Brand H, Redin CE, et al. Defining the diverse spectrum of inversions, complex structural variation, and chromothripsis in the morbid human genome. Genome Biol 2017;18:1–21. https://doi.org/10.1186/s13059-017-1158-6.
25. Lin J, Wang S, Audano PA, et al. SVision: a deep learning approach to resolve complex structural variants. Nat Methods 2022;19(10):1230–3. https://doi.org/10.1038/s41592-022-01609-w.
26. MacDonald JR, Ziman R, Yuen RK, et al. The Database of Genomic Variants: a curated collection of structural variation in the human genome. Nucleic Acids Res 2014;42(D1):D986–D992. https://doi.org/10.1093/nar/gkt958.
27. Sherry ST, Ward MH, Kholodov M, et al. dbSNP: the NCBI database of genetic variation. Nucleic Acids Res 2001;29(1):308–11. https://doi.org/10.1093/nar/29.1.308.
28. Collins RL, Brand H, Karczewski KL, et al. A structural variation reference for medical and population genetics. Nature 2020;581(7809):444–451. https://doi.org/10.1038/s41586-020-2287-8.
29. Ono Y, Asai K, Hamada M. PBSIM2: a simulator for long-read sequencers with a novel generative model of quality scores. Bioinformatics 2021;37(5):589–95. https://doi.org/10.1093/bioinformatics/btaa835.
30. Li H. Minimap2: pairwise alignment for nucleotide sequences. Bioinformatics 2018;34(18):3094–3100. https://doi.org/10.1093/bioinformatics/bty191.
31. Danecek P, Bonfield JK, Liddle J, et al. Twelve years of SAMtools and BCFtools. Gigascience 2021;10(2):giab008. https://doi.

org/10.1093/gigascience/giab008.

32. English AC, Menon VK, Gibbs RA, et al. Truvari: refined structural variant comparison preserves allelic diversity. Genome Biol 2022;23(1):271. https://doi.org/10.1186/s13059-022-02840-6.
33. Dunn T, Narayanasamy S. vcfdist: Accurately benchmarking phased small variant calls in human genomes. Nat Commun 2023;14(1):8149. https://doi.org/10.1038/s41467-023-43876-x.
34. PBSV, PacBio structural variant (SV) calling and analysis tools (version 2.9.0); 2024. https://github.com/PacificBiosciences/pbsv/releases/tag/v2.9.0, accessed 26 Oct 2024.
35. Sedlazeck FJ, Rescheneder P, Smolka M, et al. Accurate detection of complex structural variations using single-molecule sequencing. Nat Methods 2018;15(6):461–8. https://doi.org/10.1038/s41592-018-0001-7.
36. Heller D, Vingron M. SVIM: structural variant identification using mapped long reads. Bioinformatics 2019;35(17):2907–15. https://doi.org/10.1093/bioinformatics/btz041.
37. Jiang T, Liu Y, Jiang Y, et al. Long-read-based human genomic structural variation detection with cuteSV. Genome Biol 2020;21:1–24. https://doi.org/10.1186/s13059-020-02107-y.
38. Chen Y, Wang AY, Barkley CA, et al. Deciphering the exact breakpoints of structural variations using long sequencing reads with DeBreak. Nat Commun 2023;14(1):283. https://doi.org/10.1038/s41467-023-35996-1.
39. Nurk S, Koren S, Rhie A, et al. The complete sequence of a human genome. Science 2022;376(6588):44–53. https://doi.org/10.1126/science.abj6987.
40. National Center for Biotechnology Information (NCBI). Assembly database; 2024. https://www.ncbi.nlm.nih.gov/assembly, accessed: 2024-10-26.

Response letter with figures and tables

Click here to access/download
Supplementary Material
Response_letter_BVSim.pdf

Revised supplementary file

Click here to access/download
Supplementary Material
BVSim_supplement_revised.pdf
