# Supplementary material for: BVSim: A benchmarking variation simulator mimicking human variation spectrum
Source: Gigascience. 2025 Aug 30;14:giaf095. doi: 10.1093/gigascience/giaf095 (PMC12398280; doi:10.1093/gigascience/giaf095)
Supplement: giaf095_Reviewer_1_Report_Original_Submission — Nicolas Dierckxsens -- 11/29/2024 [file giaf095_reviewer_1_report_original_submission.pdf]

Reviewer Report

Title: BVSim: A Benchmarking Variation Simulator Mimicking Human Variation Spectrum

Version: Original Submission **Date:** 11/29/2024

Reviewer name: Nicolas Dierckxsens

Reviewer Comments to Author:

* I like the fact that both SNPs and a wide gamma of structural variations that can be simulated. It is also good you provide different methods of simulation. I was wondering if it is possible to simulate the exact positions of a given BED file in the wave mode?* The text is also well written, didn't notice any grammatical issues.* In the abstract you write 'we show that BVSim generated more realistic sequences significantly different from other simulators' outputs', but in the benchmark you just compare to 2 simulators, while there are many other available. You could have added one or two more or at least justify why you picked just these two…* Have you looked into RSVSim, SURVIVOR, Sim-it or SVGen, all claim to provide a more realistic distribution of position and length, probably not the only ones.* Not sure both figure 1a and the complete list of types of SVs one page 2 are needed.* It would have been nice to provide the new T2T reference genome, maybe something you can include in the future.* I did not found any system requirements in the text or SM. How long does it take to simulate a realistic SV distribution across a whole human genome, does it require virtual memory?* Not sure if I understand well, but are you arguing that BVSim is better because the two other simulators produce significantly different sequences compared to BVSim? For that you already assume from the start that BVSim is the most accurate, while that is the thing you want to prove... You don't even compare Varsim with Mutation-Simulator...

Level of Interest

Please indicate how interesting you found the manuscript: Choose an item.

Quality of Written English

Please indicate the quality of language in the manuscript: Choose an item.

Declaration of Competing Interests

Please complete a declaration of competing interests, considering the following questions:

- Have you in the past five years received reimbursements, fees, funding, or salary from an organisation that may in any way gain or lose financially from the publication of this manuscript, either now or in the future?
- Do you hold any stocks or shares in an organisation that may in any way gain or lose financially from the publication of this manuscript, either now or in the future?

- Do you hold or are you currently applying for any patents relating to the content of the manuscript?
- Have you received reimbursements, fees, funding, or salary from an organization that holds or has applied for patents relating to the content of the manuscript?
- Do you have any other financial competing interests?
- Do you have any non-financial competing interests in relation to this paper?

If you can answer no to all of the above, write 'I declare that I have no competing interests' below. If your reply is yes to any, please give details below.

I declare that I have no competing interests

I agree to the open peer review policy of the journal. I understand that my name will be included on my report to the authors and, if the manuscript is accepted for publication, my named report including any attachments I upload will be posted on the website along with the authors' responses. I agree for my report to be made available under an Open Access Creative Commons CC-BY license (http://creativecommons.org/licenses/by/4.0/). I understand that any comments which I do not wish to be included in my named report can be included as confidential comments to the editors, which will not be published.

Choose an item.

To further support our reviewers, we have joined with Publons, where you can gain additional credit to further highlight your hard work (see: https://publons.com/journal/530/gigascience). On publication of this paper, your review will be automatically added to Publons, you can then choose whether or not to claim your Publons credit. I understand this statement.

Yes Choose an item.
