# Supplementary material for: BVSim: A benchmarking variation simulator mimicking human variation spectrum
Source: Gigascience. 2025 Aug 30;14:giaf095. doi: 10.1093/gigascience/giaf095 (PMC12398280; doi:10.1093/gigascience/giaf095)
Supplement: giaf095_Reviewer_2_Report_Original_Submission — Sina Majidian -- 1/22/2025 [file giaf095_reviewer_2_report_original_submission.pdf]

Reviewer Report

Title: BVSim: A Benchmarking Variation Simulator Mimicking Human Variation Spectrum

Version: Original Submission **Date:** 1/22/2025

Reviewer name: Sina Majidian

Reviewer Comments to Author:

The manuscript describes BVSim, a tool for simulating a diverse range of genetic variations, including SNVs and SVs. The main contribution of the software is that the generated variants follow the real SV profile rather than being uniform. The software could potentially be used by software developers. Overall, the manuscript is well-structured and well-written. I was able to run the software, but there are several points that could improve the software and the manuscript. Below are detailed suggestions and points requiring clarification or improvement to enhance the quality and reproducibility of the work.1. The manuscript lacks methodological details on how the distribution is learned and whether a parameter-fitting approach was used. Also, it is unclear how variants are generated. I'm wondering how the variant types other than indels are modeled. Are their parameters/models inferred using the Cell dataset? I'm wondering why one mode of the software is called "wave-mode"?2. I'm wondering whether there is potential circularity in which the SVs are generated based on a specific set of SVs that are inferred by a certain tool, making it easier for this tool to identify the SVs.3. In Figure 1b and 1c, the differences in F1 scores between BVsim and the other simulators are positive for most cases. Does this indicate that it is easier for tools to call SVs generated by BVSim? It would be interesting to see actual values of F1 scores. Also, I'm curious to know whether the performance ranking of SV callers are different across simulators.4. Using 15 samples seems small. Please consider using larger datasets such as gnomAD (gnomad.broadinstitute.org/news/2023-11-v4-structural-variants/), All of Us, HGSVC collection (internationalgenome.org/data-portal/data-collection) or doi.org/10.1038/s41467-024-53620-8. One idea would be to generate population-specific variants and allow users to input population names as an argument.5. Statistics on runtime and memory footprint across SV simulators should be reported, it would be best to average over 5-10 replicates.6. It seems that BVsim performs SV simulation only on the first fasta record (chromosome) when multiple records are present in a FASTA file. Is it a bug?7. There are two other tools SURVIVOR (github.com/fritzsedlazeck/SURVIVOR) and RSVSim (doi.org/10.1093/bioinformatics/btt198) which could be mentioned in the text.8. It would be helpful to add a small test example along with its dotplot (with mummerplot) as supplementary figure where the generated SVs are annotated on the figure.9. The manuscript used hg19 and hg39 reference genomes. Please include results for T2T-CHM13 or discuss the potential differences in performance of BVSim with this reference.10. It would be useful to generate phased SVs specific to each of haplotypes. Phasing the variants of diploids/polyploids is an important problem, and there are tools that can now handle diploid data.11. Can BVsim generate translocations that occur across two chromosomes? Some other examples of complex SVs are discussed here github.com/jzook/testHG008curation/issues12. I'm not sure the list of 18 IDs is needed in the text. All could be mentioned in Figure 1a.13. Thanks for providing the code used for simulation. Please provide the version of SV callers to ensure results can be reproduced.----

Comments on software14. I was able to install the software using conda which was smooth. Please provide the default values for all arguments as output of python -m BVSim -h, and specify which arguments are mandatory. Ensure that generated VCF files are sorted.15. That's great BVsim VCFs work with truvari. Does it work with vcfdist, hap.py, SURVIVOR and rtg vcfeval too?16. Please clarify the compatibility of the output VCF file with VCF versions 4.3 and 4.4.17. Detailed descriptions of command-line arguments (e.g., -times, -rep) and their possible ranges should be provided on github. Also, please make releases on github with named version.18. With the default parameters and seeding, I see two deletions generated at the same position. Is it happening often?19. It would be better if the package could be run from any location without being limited to the BVSim github folder.20. I saw instructions on GitHub for generating BED files. However, it would be more user-friendly for BVSim to have an option that accepts multi-sample VCFs from which the distribution of SVs can be extracted.21. All arguments should be tested thoroughly. I was able to run the package with `python -m BVSim -ref refa.fa -save out` but when I added `-block_region_bed_url out.bed`, I faced this error: uniform.py, line 31, in check_start_end if row['start'] >= row['end']: TypeError: '>=' not supported between instances of 'str' and 'int'.When I added -dupmax 20, I faced this uniform.py, line 605, in main tem_copied_base = np.random.choice(dup_range) File numpy/random/mtrand.pyx, line 970, in numpy.random.mtrand.RandomState.choice.When I added -snv_del 0.4, I faced this uniform.py", line 889, in main r_s = sample(unblock_region_sv,1)[0] File python3.11/random.py, line 456, in sample raise ValueError("Sample larger than population or is negative").

Level of Interest

Please indicate how interesting you found the manuscript: Choose an item.

Quality of Written English

Please indicate the quality of language in the manuscript: Choose an item.

Declaration of Competing Interests

Please complete a declaration of competing interests, considering the following questions:

- Have you in the past five years received reimbursements, fees, funding, or salary from an organisation that may in any way gain or lose financially from the publication of this manuscript, either now or in the future?
- Do you hold any stocks or shares in an organisation that may in any way gain or lose financially from the publication of this manuscript, either now or in the future?
- Do you hold or are you currently applying for any patents relating to the content of the manuscript?
- Have you received reimbursements, fees, funding, or salary from an organization that holds or has applied for patents relating to the content of the manuscript?
- Do you have any other financial competing interests?

- Do you have any non-financial competing interests in relation to this paper?

If you can answer no to all of the above, write 'I declare that I have no competing interests' below. If your reply is yes to any, please give details below.

I declare that I have no competing interests.

I agree to the open peer review policy of the journal. I understand that my name will be included on my report to the authors and, if the manuscript is accepted for publication, my named report including any attachments I upload will be posted on the website along with the authors' responses. I agree for my report to be made available under an Open Access Creative Commons CC-BY license (http://creativecommons.org/licenses/by/4.0/). I understand that any comments which I do not wish to be included in my named report can be included as confidential comments to the editors, which will not be published.

Choose an item.

To further support our reviewers, we have joined with Publons, where you can gain additional credit to further highlight your hard work (see: https://publons.com/journal/530/gigascience). On publication of this paper, your review will be automatically added to Publons, you can then choose whether or not to claim your Publons credit. I understand this statement.

Yes Choose an item.
