# Supplementary material for: BVSim: A benchmarking variation simulator mimicking human variation spectrum
Source: Gigascience. 2025 Aug 30;14:giaf095. doi: 10.1093/gigascience/giaf095 (PMC12398280; doi:10.1093/gigascience/giaf095)
Supplement: giaf095_Reviewer_3_Report_Original_Submission — National Institute Technology -- 2/14/2025 [file giaf095_reviewer_3_report_original_submission.pdf]

Reviewer Report

Title: BVSim: A Benchmarking Variation Simulator Mimicking Human Variation Spectrum

Version: Original Submission **Date:** 2/14/2025

Reviewer name: National Institute Technology

Reviewer Comments to Author:

This manuscript introduces BVSim, a flexible tool for simulating genomic variationsâ€”particularly structural variants (SVs)â€”to facilitate variant calling benchmark studies. BVSim appears promising for modeling both simple and complex genomic rearrangements, with userâ€�friendly modes to specify different distributions and variation parameters. The manuscript is generally well structured and clearly describes the impetus for improved SV simulators. However, there are key points regarding clarity of methods, representativeness of input data, comparison to existing simulators, and data/code organization that should be addressed before publication.Comments1. Clarification of Scope and Terminology: The term "micro variants" is used but not defined. Suggest using the commonly used term "small variants" instead of "micro variants" if appropriate.2. Representativeness of Input Data: The Discussion should explicitly address how the representativeness (and possible biases) of the userâ€�provided SV call sets affects the full spectrum of simulated SVs. If a provided call set underrepresents complex or tandemâ€�repeat regions, the simulator's output might mirror those biases. For example the GIAB v0.6 SV benchmark set only includes isolated insertions and deletions and does not represent the full diversity of SV in a human genome.3. Handling Different Ploidy Levels: The manuscript focuses on diploid human genome references. Please clarify whether BVSim can be applied (without major modifications) to polyploid organisms (e.g., crops), haploid references (e.g., microbial genomes), as well as somatic samples, and if so how. If not, discuss the limitations or future directions for supporting nonâ€�diploid references.4. Comparison with Existing Simulators: Figure 1 shows that BVSim achieves a higher F1 score than certain methods (VarSim, Mutationâ€�Simulator), but the reasons should be explained more thoroughly. If BVSim is able to simulate more realistic and complex variants I would expect the F1 score to be lower for BVSim compared to other methods. Additionally, `truvari bench` does not correctly compare complex variants and `truvari refine` should be use to accurately benchmark complex SVs.5. Comparison with realistic benchmark set: Comparing benchmarking results between simulators and variant size distributions are not real indicators of a realistic simulation. A better evaluation would be comparing benchmarking results for a variant callset generated using simulated data and a SV callset generated from real sequencing data against a SV benchmark set.6. Simulation Modes and Parameters: CSV mode: Clarify more explicitly whether nonâ€�complex SVs (e.g., simple deletions/insertions) are still simulated alongside complex variants when the user employs CSV mode.7. Data and Code Organization: The shared data repository appears to contain many intermediate or temporary files. A more curated data release that omits unnecessary files would reduce confusion and improve accessibility. The data README should focus on directory structures and file naming conventions rather than listing every file individually. For the code shared to reproduce the analysis presented in the manuscript, removing hardâ€�coded system paths in

scriptsâ€"favoring relative pathsâ€"increases portability. Provide yaml or similar requirements files for conda environments used.8. BVSim: Provide a YAML/requirements.txt (or similar) to specify all dependencies (Python libraries, etc.) and ease environment setup and installation, potentially a docker container as well. BVSim includes numerous configurable parameters. Suggest providing a single YAML/JSON config file with wellâ€�chosen defaults for typical human genome simulations. An example template would help new users get started and also ensure reproducible benchmarks.9. Figure 2 (workflow diagram), highlight which files are userâ€�provided inputs (e.g., reference, BED with SV call sets, parameter config) and which files are outputs (e.g., FASTA with embedded variants, VCF, logs) to enhance clarity.

Level of Interest

Please indicate how interesting you found the manuscript: Choose an item.

Quality of Written English

Please indicate the quality of language in the manuscript: Choose an item.

Declaration of Competing Interests

Please complete a declaration of competing interests, considering the following questions:

- Have you in the past five years received reimbursements, fees, funding, or salary from an organisation that may in any way gain or lose financially from the publication of this manuscript, either now or in the future?
- Do you hold any stocks or shares in an organisation that may in any way gain or lose financially from the publication of this manuscript, either now or in the future?
- Do you hold or are you currently applying for any patents relating to the content of the manuscript?
- Have you received reimbursements, fees, funding, or salary from an organization that holds or has applied for patents relating to the content of the manuscript?
- Do you have any other financial competing interests?
- Do you have any non-financial competing interests in relation to this paper?

If you can answer no to all of the above, write 'I declare that I have no competing interests' below. If your reply is yes to any, please give details below.

I declare that I have no competing interests

I agree to the open peer review policy of the journal. I understand that my name will be included on my report to the authors and, if the manuscript is accepted for publication, my named report including any attachments I upload will be posted on the website along with the authors' responses. I agree for my

report to be made available under an Open Access Creative Commons CC-BY license (http://creativecommons.org/licenses/by/4.0/). I understand that any comments which I do not wish to be included in my named report can be included as confidential comments to the editors, which will not be published.

Choose an item.

To further support our reviewers, we have joined with Publons, where you can gain additional credit to further highlight your hard work (see: https://publons.com/journal/530/gigascience). On publication of this paper, your review will be automatically added to Publons, you can then choose whether or not to claim your Publons credit. I understand this statement.

Yes Choose an item.
